# Responses of the carotid artery to acute, fractionated or chronic ionizing irradiation, and differences from the aorta

**DOI:** 10.1038/s41598-025-92710-5

**Published:** 2025-03-05

**Authors:** Nobuyuki Hamada, Ki-ichiro Kawano, Seiko Hirota, Farina Mohamad Yusoff, Takaharu Nomura, Yusuke Saito, Ayumu Nakashima, Shinji Yoshinaga, Yukihito Higashi

**Affiliations:** 1 Biology and Environmental Chemistry Division, Sustainable System Research Laboratory, Central Research Institute of Electric Power Industry (CRIEPI), 1646 Abiko, Chiba, 270-1194 Japan; 2 Department of Regenerative Medicine, Division of Radiation Medical Science, Research Institute for Radiation Biology and Medicine, Hiroshima University, Kasumi 1-2-3, Minamiku, Hiroshima, Hiroshima 734-8553 Japan; 3 Department of Environmetrics and Biometrics, Division of Radiation Basic Science, Research Institute for Radiation Biology and Medicine, Hiroshima University, Kasumi 1-2-3, Minamiku, Hiroshima, Hiroshima 734-8553 Japan; 4 Hiroshima University School of Medicine, Kasumi 1-2-3, Minamiku, Hiroshima, Hiroshima 734-8551 Japan; 5 Department of Stem Cell Biology and Medicine, Graduate School of Biomedical and Health Sciences, Hiroshima University, Kasumi 1-2-3, Minamiku, Hiroshima, Hiroshima 734-8551 Japan; 6 Division of Regeneration and Medicine, Medical Center for Translational and Clinical Research, Hiroshima University Hospital, Kasumi 1-2-3, Minamiku, Hiroshima, Hiroshima 734-8551 Japan

**Keywords:** Ionizing radiation, Dose rate, Fractionation, Carotid artery, Aorta, Biophysics, Environmental sciences, Cardiology, Risk factors

## Abstract

The circulatory system receives ionizing radiation at various dose rates. Given mounting epidemiological evidence of elevated radiation risks for diseases of the circulatory system (DCS), the International Commission on Radiological Protection recently recommended the first ever dose threshold for DCS. However, very little knowledge exists about whether radiation effects differ with dose rates and among tissues of the circulatory system. Here, we investigated the impact of dose rates in the carotid artery (CA) and compared it with the aorta. CA was obtained from mice irradiated with the same total dose that was delivered either acutely, 25 fractions, 100 fractions or chronically. CA underwent immunofluorescence and histochemistry staining. Irradiation led to vascular damage, inflammation and fibrosis in CA. The integrative analysis for 14 prelesional endpoints revealed that the magnitude of carotid changes was greater in 25 fractions, smaller in 100 fractions, and much smaller in chronic irradiation, compared with acute irradiation. Radiation responses of the aorta were qualitatively similar to, but quantitatively greater than those of CA. Irradiation causes sparing and enhancing dose protraction effects in a manner that is not a simple function of dose rate, and that radiosensitivity varies within the circulatory system.

**Supplementary Information:**

The online version contains supplementary material available at 10.1038/s41598-025-92710-5.

## Introduction

Sensitivity to ionizing radiation exposure varies among parts of the body, and a priority in radiation risk management is protection of the radiosensitive parts. The circulatory system had long been considered radioresistant, because complications after fractionated radiotherapy for cancer were known to occur at high dose (e.g., 40–60 Gy for pericarditis)^1^. Reports on elevated risks for diseases of the circulatory system (DCS) at much lower dose, however, began to appear in the 1990s. In particular, the study that reported in 2010 an increased DCS mortality risk above 0.5 Gy in acutely exposed Japanese atomic bomb survivors^2^ was most influential in leading the International Commission on Radiological Protection (ICRP) in 2011 to recommend the first ever dose threshold for DCS^3^. ICRP recommended the same dose threshold for DCS independent of dose rate (radiation dose absorbed per unit time), while recognizing notable uncertainties due to limited scientific evidence to judge that dose rate does not change the magnitude of risk^3^.

Epidemiological evidence for DCS has accumulated since 2011, and the meta-analysis of the 93 studies confirmed radiation risks above 0.5 Gy (e.g., 2–4 deaths from DCS estimated among 100 people exposed to 1 Gy)^4^. Questions critical for radiation risk management, nevertheless, remain unanswered, such as regarding how dose rate modifies radiation effects on the circulatory system, how radiosensitivity differs within the circulatory system, and which tissues/organs are responsible targets whose radiation exposure causes DCS. Consideration of dose rate is pivotal, because the circulatory system receives radiation at wide-ranging dose rates, e.g., acutely (e.g., as a single dose during radiotherapy for refractory ventricular tachycardia^5^) or intermittently (e.g., for multiple diagnostic imaging procedures, or in 20–40 daily fractions during radiotherapy for cancer) in patients, and more protractedly or chronically in radiation workers (e.g., interventional cardiologists and nuclear workers).

Targets for radiogenic DCS remain unidentified, but potential candidates include large blood vessels (e.g., the carotid artery and the aorta) in addition to the heart and kidneys^6^. The role of the carotid artery has been suggested epidemiologically, because cerebrovascular disease has been associated with radiation dose to the thyroid or salivary gland (a surrogate for dose to the carotid artery) rather than that to the brain^4,7^. Carotid artery stenosis also represents a typical complication of fractionated radiotherapy for head and neck cancer^8^. It is nonetheless evident from Table S1 that there is little knowledge about radiation responses of the carotid artery, and that in most studies, atherosclerosis-prone models (e.g., via hypercholesterolemia or partial carotid ligation) were irradiated acutely with a single dose. Of these, Hoving et al. conducted acute irradiation and fractionated irradiation of apolipoprotein E (ApoE)-deficient mice, albeit at different doses and in different sexes^9^, making comparisons between irradiation regimens difficult: therefore, the impact of dose rate remains unknown. In a later study, they further found that radiation-induced atherogenesis varies between the carotid artery and the brachiocephalic artery in ApoE^–/–^ mice^10^, suggesting that radiation responses differ among large vessels; however, no such comparison has been made in wildtype animals. For investigating radiation responses at various dose rates, it is important to use wildtype animals, because disease models tend to exhibit hormetic responses to chronic irradiation especially at low or moderate dose^11,12^.

In the present study, we evaluated 14 endpoints in the carotid artery of wildtype mice, which was obtained at two timepoints after acute, intermittent or chronic irradiation of the total body with the same total dose of X- or γ-rays, or obtained from nonirradiated young or aged control mice. We further compared the carotid artery and the aorta. With the robust datasets obtained from 22 groups of mice, we demonstrate that irradiation causes sparing and enhancing dose protraction effects in a fashion that is not a simple function of dose rate and the number of fractions, that the aorta is more responsive to irradiation than the carotid artery, and that changes observed in the irradiated tissues are qualitatively similar to those in the aged tissues. These findings contribute to ongoing discussions to develop international recommendations for management of radiation risk and optimization of radiation protection in various exposure scenarios.

## Materials and methods

### Mice, irradiation and sampling

**Fig. 1:**
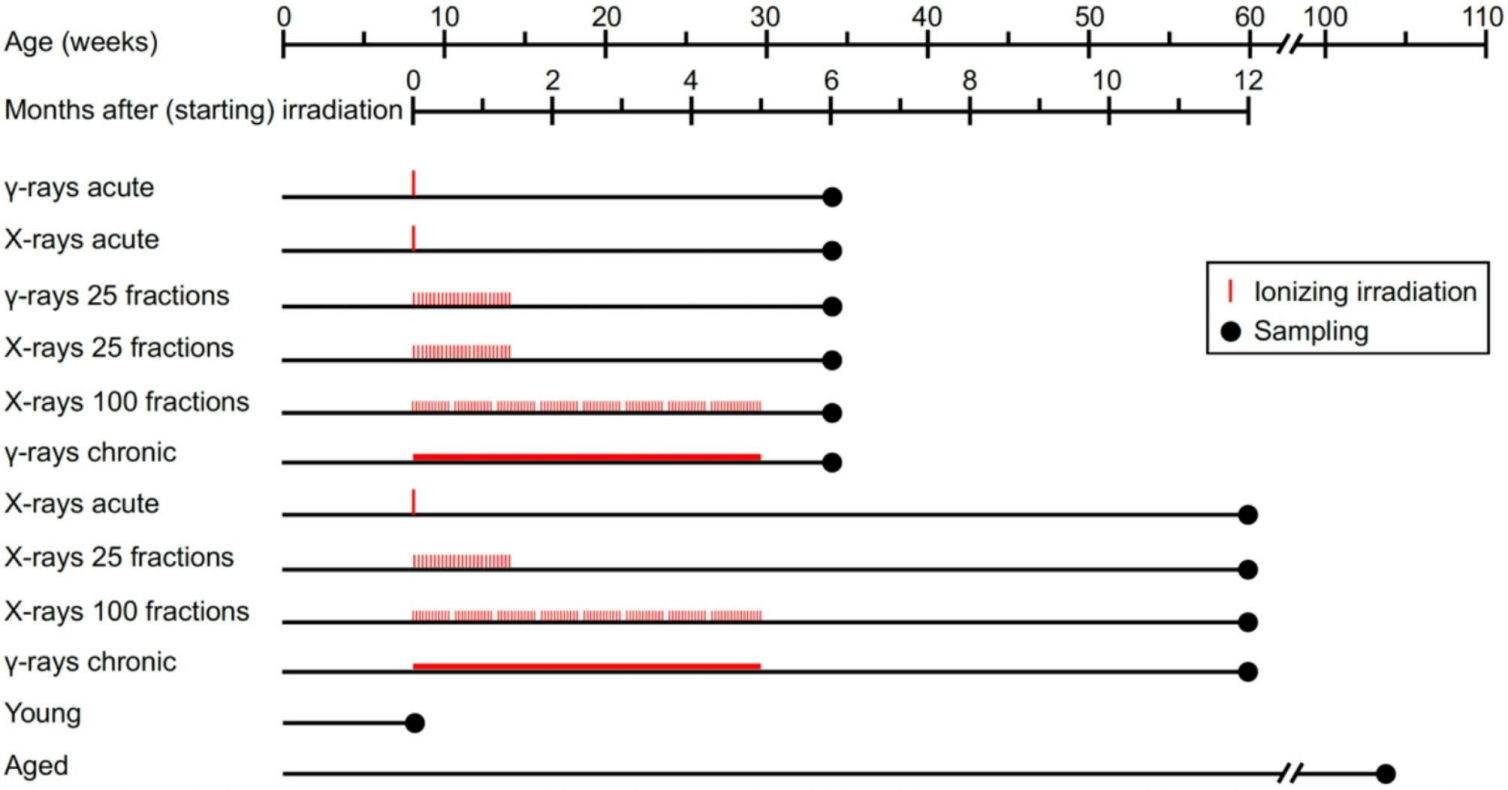
Experimental timelines. The present study is composed of 22 groups of male C57BL/6J mice. Mice aged 8 weeks were irradiated with 0–5 Gy of X-rays or γ-rays, either acutely (in 10 min), intermittently (in 25 fractions over 42 days, in 100 fractions over 153 days) or chronically (over 153 days). Tissues were sampled at 6 or 12 months after starting irradiation, and also sampled from two nonirradiated control groups (“young” aged 8 weeks and “aged” aged 104 weeks). For more details, see the main text, Supplemental Methods and Fig. S1.

Figure 1 schematizes 22 groups of male C57BL/6J mice (totaling 215 mice) that composed the study. Unanesthetized mice aged 8 weeks were total body irradiated with 5 Gy of X- or γ-rays acutely as a single dose (referred hereinafter to as “acute X-rays” or “acute γ-rays”, delivered continuously in 10 min at 30 Gy/h) or intermittently in 25 fractions spread over 42 days (“X-rays in 25 fractions” or “γ-rays in 25 fractions”, delivered as 0.2 Gy/fraction in 24 s at 30 Gy/h, 4.2 fractions/week, virtually 5.0 mGy/h), X-rays intermittently in 100 fractions over 153 days (“X-rays in 100 fractions”, delivered as 0.05 Gy/fraction in 6 s at 30 Gy/h, 4.6 fractions/week, virtually 1.4 mGy/h), or γ-rays chronically over 153 days (“chronic γ-rays”, delivered continuously at 1.4 mGy/h). As such, the study used the same dose rate (30 Gy/h) for acute irradiation and in each fraction for fractionated irradiation, but used a different dose rate (1.4 mGy/h) for chronic irradiation. If we define total dose resulting from multiple fractions divided by the entire period of time for dose delivery (i.e., inclusive of inter-fraction intervals besides the net irradiation time) as an average dose rate^13,14^, then the study used the same average dose rate (1.4 mGy/h) for irradiation in 100 fractions and chronic irradiation, but used the different average dose rate (5.0 mGy/h) for irradiation in 25 fractions. To avoid confusion for different temporal dose distributions among irradiation regimens, we hereinafter use the dose rate for continuous irradiation (i.e., acute irradiation and chronic irradiation) and the number of fractions for intermittent irradiation in 25 fractions and in 100 fractions.

For the “acute X-rays”, “X-rays in 25 fractions” and “X-rays in 100 fractions” regimens, mice were irradiated at CRIEPI with X-rays (260 kVp and 4.5 mA with a 0.5 mm Al and 0.3 mm Cu filter, a source-surface distance of 52.7 cm) from a Faxitron MultiRad350 irradiator. For the “chronic γ-rays” regimen, mice were irradiated at CRIEPI with ^137^Cs γ-rays (662 keV, a source-surface distance of 371 cm). For the “acute γ-rays” and “γ-rays in 25 fractions” regimens, mice were irradiated at Hiroshima University with ^137^Cs γ-rays (662 keV) from Gammacell 40 Exactor. Dosimetry was performed with a Dose Ace system consisting of radiophotoluminescent glass rods of GD-300 series (GD-351 for X-rays and GD-301 for γ-rays at CRIEPI, and GD-302 M for γ-rays at Hiroshima University) and an FDG-1000 reader, all from Asahi Techno Glass Co. Ltd. (Shizuoka, Japan), as described^15–17^.

For each irradiation regimen, control mice were sham-irradiated in parallel with the test mice. Prior to tissue sampling, animals were anesthetized with 1.5% isoflurane by inhalation at a flow rate of 2 L/min. The carotid artery and the aorta were sampled from the same mice at 6 months (at age 34 weeks) after starting irradiation with six regimens (12 groups), and at 12 months (at age 60 weeks) after starting irradiation with four (all regimens used for the 6-month timepoint, save “acute γ-rays” and “γ-rays in 25 fractions”) regimens (8 groups). These tissues were also sampled from nonirradiated mice (2 groups) at age 8 (“young”) or 104 (“aged”) weeks. Mice that experience a severe body weight reduction (≥ 20% in 3 days) are supposed to undergo euthanasia by cervical dislocation; however, no mice were actually subjected to euthanasia because no mice showed such severe weight loss. All mice were purchased from Jackson Laboratory Japan (Kanagawa or Shiga, Japan). All animal experiments were approved by the Animal Research and Ethics Committee of CRIEPI (approval number 2016–08) and the Institutional Animal Care and Use Committee of Hiroshima University (approval number A16–139), and performed in compliance with the Japanese guidelines of animal care and the ARRIVE guidelines. See Supplementary Methods and Fig. S1 for further details.

### Immunofluorescence and histochemistry staining

The frozen samples were transversally cryosectioned at a 5-µm thickness (Leica CM1950), and sections were mounted onto glass slides for staining. For dual immunofluorescence, sections were fixed in 4% paraformaldehyde and blocked. CD31 was stained green (Alexa Flour 488), one of the ten other markers (α-SMA, eNOS, VE-cadherin, TNF-α, CD68, F4/80, CD3, TGF-β1, cleaved caspase-3, cleaved N-terminal GSDMD) being stained red (Cy3 for α-SMA, Alexa Flour 594 for other markers), with antibodies listed in Table S2 and cell nuclei counterstained with 4’,6-diamidino-2-phenylindole (DAPI). For histochemistry, Masson’s trichrome staining (where aniline blue stains collagen fibers in the tunica media blue: Muto Pure Chemicals, Tokyo, Japan), Oil Red O staining (to stain neutral lipids in the atherosclerotic plaques red: ScyTek Laboratories, Logan, UT, USA) with cell nuclei counterstained with Mayer’s hematoxylin (Lillie’s modification), and TUNEL staining (to stain cells with DNA fragmentation: Takara, Shiga, Japan) were conducted according to the manufacturer’s instructions. As a positive control for TUNEL staining, sections were treated for 30 min at 37˚C with RNase-free recombinant DNase I (Takara, Shiga, Japan) at 0.05 U/µl. The intima-media thickness (IMT) measured from immunofluorescence and histochemistry images were designated IMT-IF and IMT-HC, respectively. Images were captured and quantitatively analyzed, as detailed in Supplementary Methods.

### Statistical analysis

The data for the carotid artery were newly obtained in the present study. The data for the aorta taken from previous studies^18–21^ were reanalyzed. Each data was obtained from 8 to 10 mice and is presented as means and standard deviations, unless otherwise described. Statistical analyses were conducted using R statistical software (version 4.2.2, R Foundation, https://www.r-project.org/). Normality was assessed by the Shapilo-Wilk test. *P* values were determined by parametric tests (e.g., Welch’s two-sample t-test, paired-samples t-test), non-parametric tests (e.g., Wilcoxon rank sum test, Wilcoxon signed rank test), the analysis of deviance, Wald test (logistic regression), or the linear regression analysis, as specified in figure legends or table footnotes. To compare the magnitude of effects among various irradiation regimens, the integrative analysis was performed with the Kolmogorov–Smirnov test (see Supplementary Methods for details). *P* values of < 0.05 were considered significant (*P* < 0.001 presented as **, 0.001 ≤ *P* < 0.05 as *), 0.05 ≤ *P* < 0.1 as marginally significant (presented as #) and *P* ≥ 0.1 as nonsignificant (presented as ns).

## Results

### Irradiation induces vascular damage, inflammation and fibrosis in the carotid artery

To evaluate radiation-induced changes, the carotid artery obtained from 10 irradiated or 10 sham-irradiated groups of mice (Fig. 1) was subjected to immunofluorescence and histochemistry staining.

**Fig. 2:**
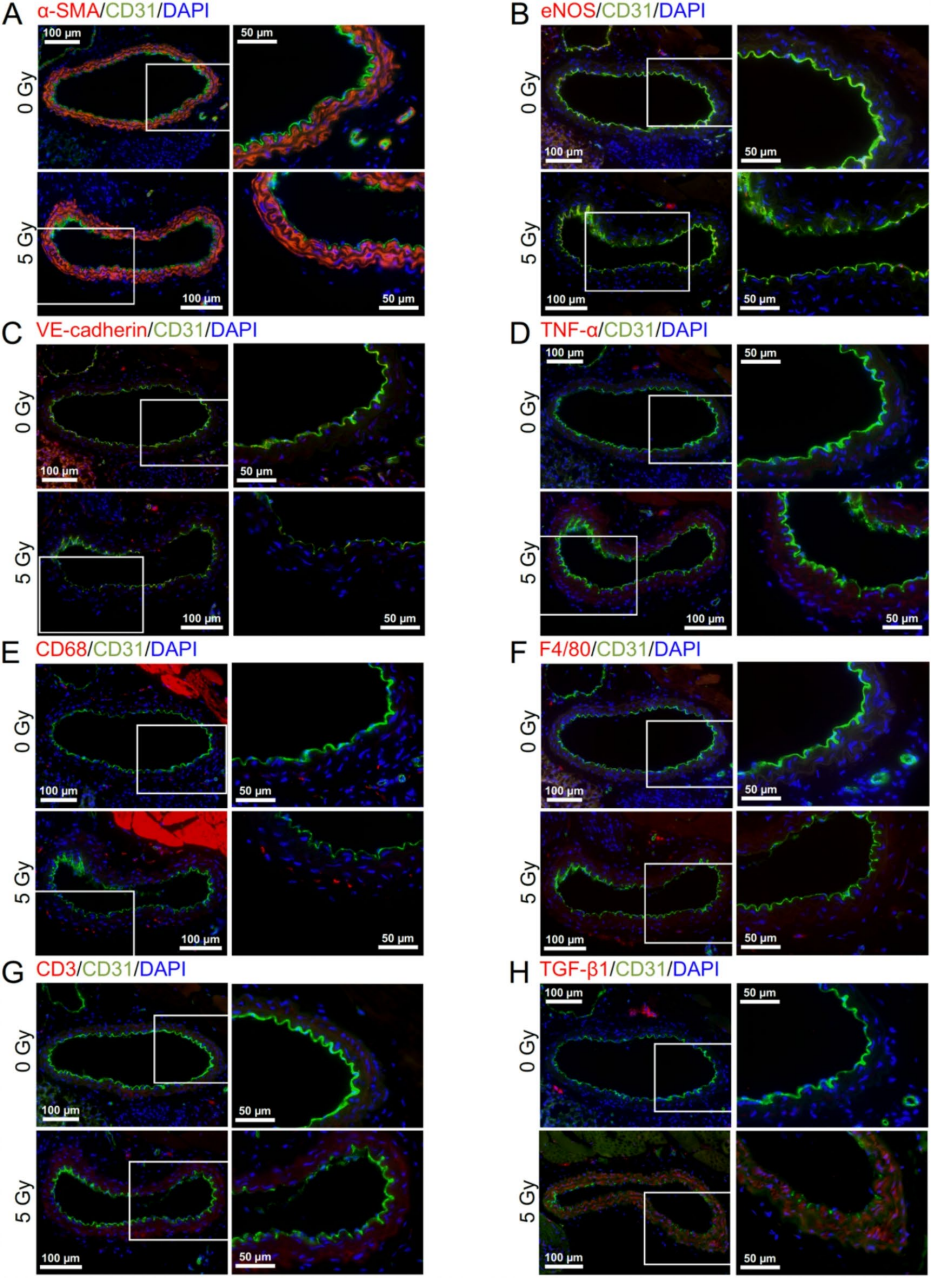
Representative immunofluorescence images in the carotid artery. The carotid artery underwent dual immunofluorescence of CD31 (green) with (**A**) α-SMA, (**B**) eNOS, (**C**) VE-cadherin, (**D**) TNF-α, (**E**) CD68, (**F**) F4/80, (**G**) CD3 or (**H**) TGF-β1 (red), with cell nuclei counterstained with DAPI (blue). Images were taken at 6 months after starting irradiation with 0 Gy (upper panels) or 5 Gy (lower panels) of γ-rays in 25 fractions. Boxed areas in the left panels (tiled images) are shown at higher magnification in the right panels. Scale bars are as indicated. For clarity, the image at much higher magnification is shown for α-SMA in Fig. S2.

**Fig. 3:**
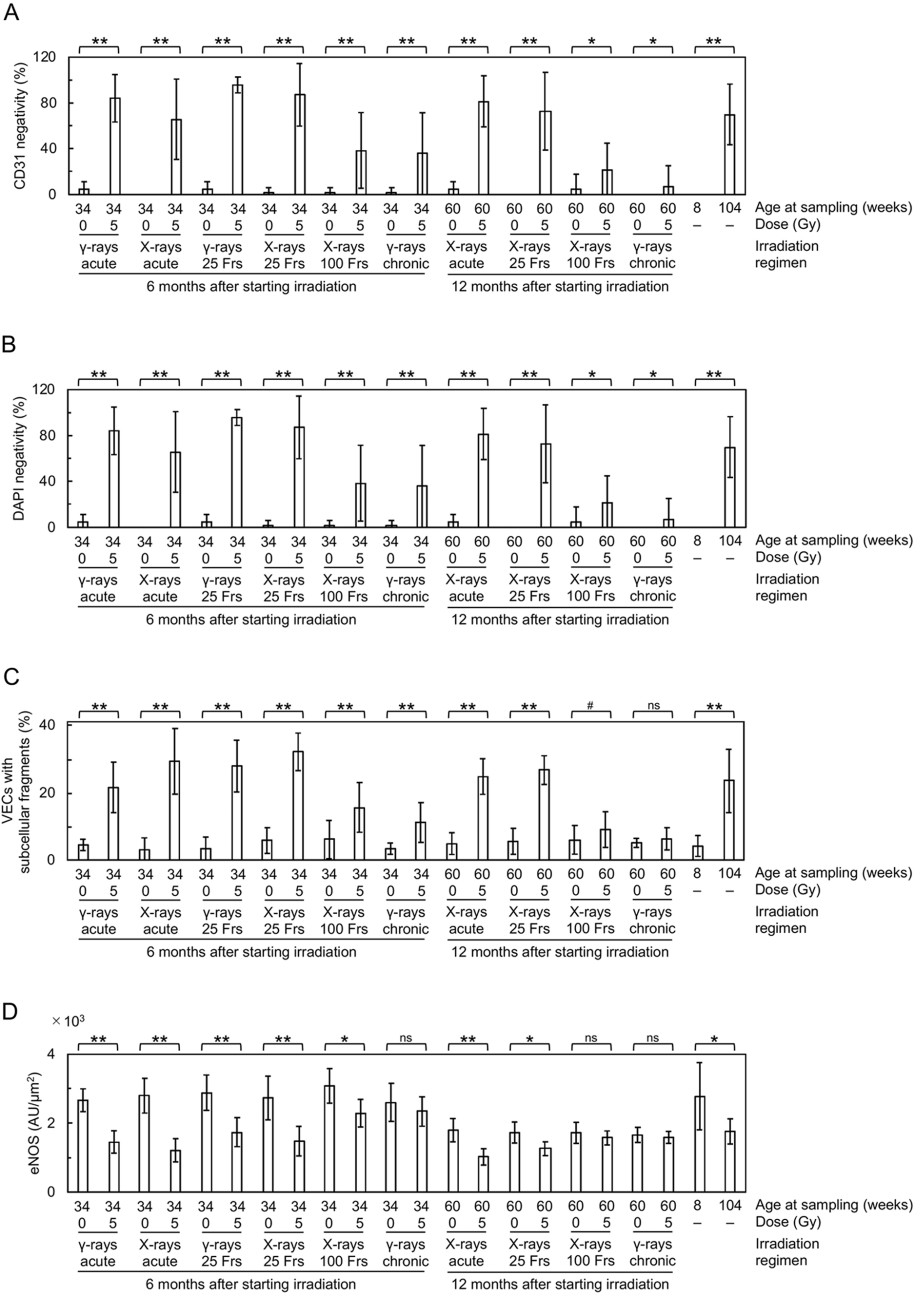

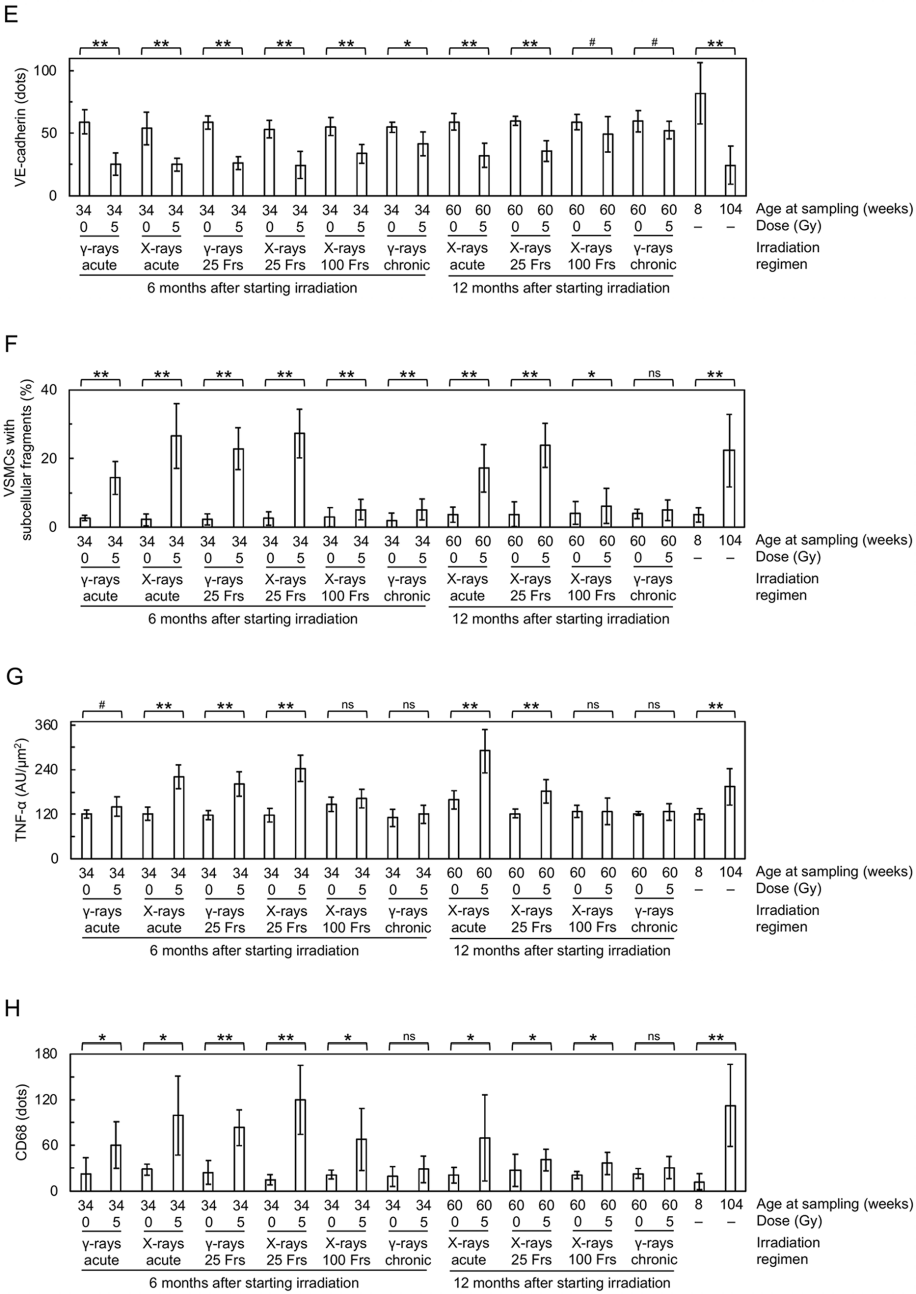

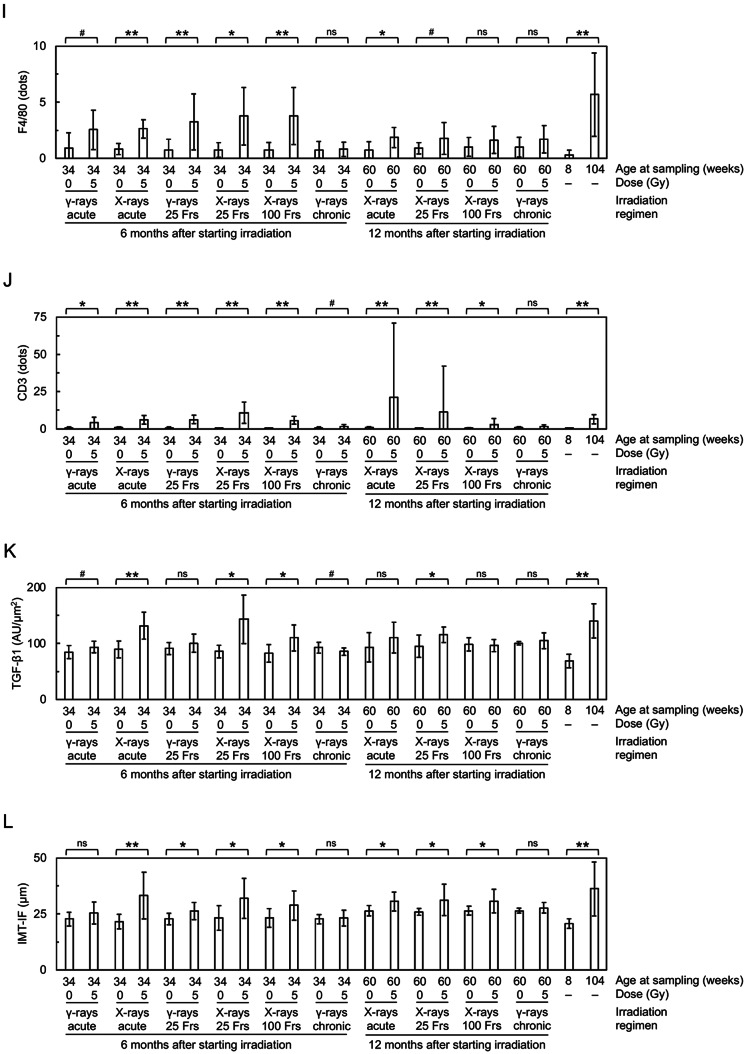
Quantitative analysis of immunofluorescence images in the carotid artery. Comparisons between the two groups (sham-irradiated vs. irradiated in each irradiation regimen, young vs. aged) were made for (**A**) CD31 negativity, (**B**) DAPI negativity, (**C**) VECs with subcellular fragments, (**D**) eNOS, (**E**) VE-cadherin, (**F**) VSMCs with subcellular fragments, (**G**) TNF-α, (**H**) CD68, (**I**) F4/80, (**J**) CD3, (**K**) TGF-β1, and (**L**) IMT-IF. *n* = 8–10 mice/group totaling 215 mice in 22 groups. Frs, fractions. AU, arbitrary units. *P* by the Welch’s t-test, Wilcoxon rank sum test, or Wald test. **P < 0.001. *0.001 ≤ *P* < 0.05. #, 0.05 ≤ *P* < 0.1 (marginally significant), ns, *P* ≥ 0.1 (nonsignificant). See Fig. 2 and S3A for representative images. For clarity, a graph for CD3 is replotted in Fig. S4 for the lower y-axis values. See Fig. S5 for corresponding data in the aorta and Fig. S6 for comparisons between IMT-IF and IMT-HC.

**Fig. 4:**
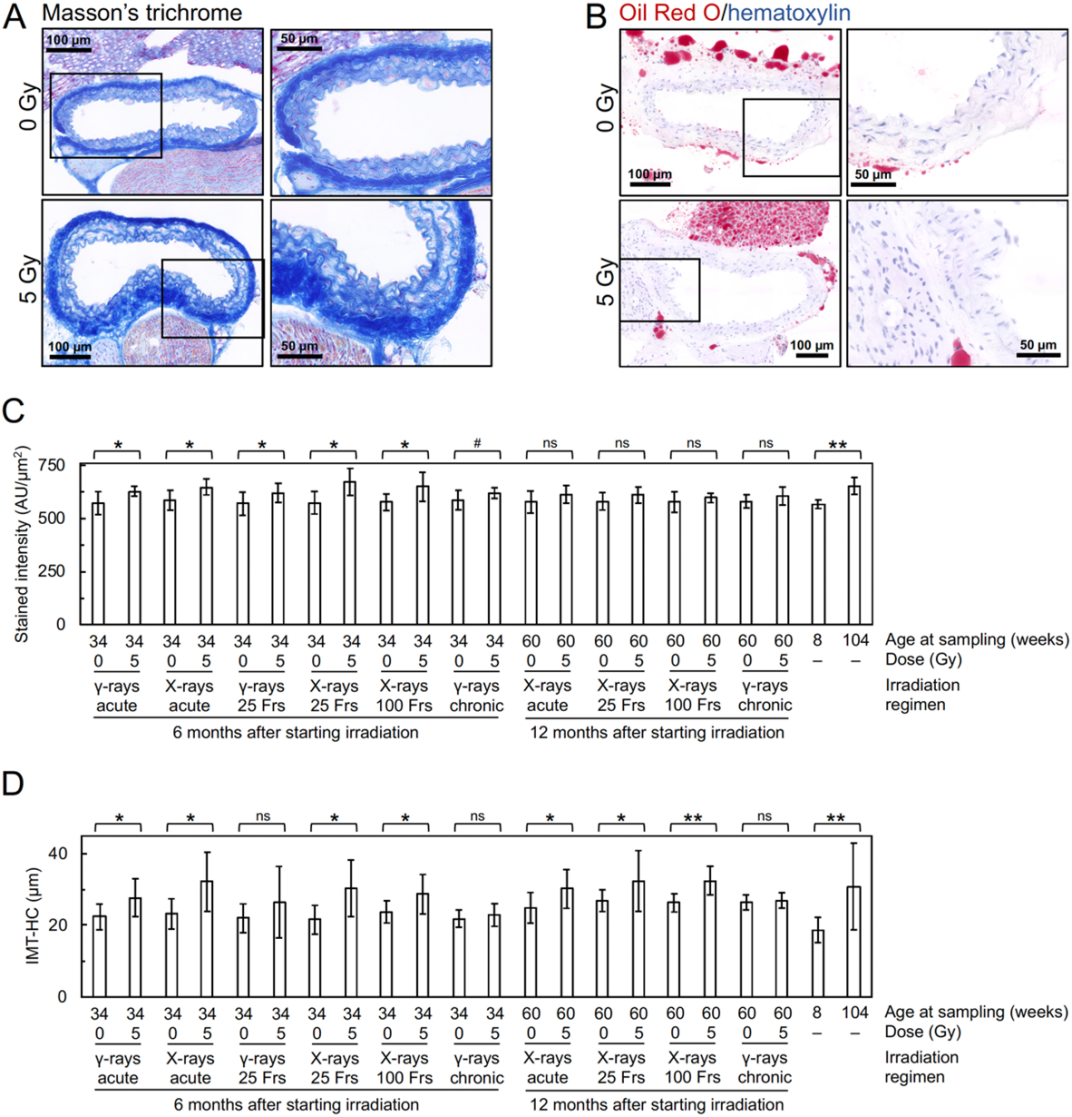
Representative histochemistry images and its quantitative analysis in the carotid artery. The carotid artery underwent (**A**) Masson’s trichrome staining and (**B**) Oil Red O staining. Images were taken at 6 months (**A**) or 12 months (**B**) after starting irradiation with 0 Gy (upper panels) or 5 Gy (lower panels) of acute γ-rays. Boxed areas in the left panels (tiled images) are shown at higher magnification in the right panels. Scale bars are as indicated. Comparisons between the two groups (sham-irradiated vs. irradiated in each irradiation regimen, young vs. aged) were made for (**C**) intensity of aniline blue per unit carotid wall area, and (**D**) IMT-HC. *n* = 8–10 mice/group totaling 215 mice in 22 groups. Frs, fractions. AU, arbitrary units. *P* by the Welch’s t-test or Wilcoxon rank sum test. **P < 0.001. *0.001 ≤ *P* < 0.05. #, 0.05 ≤ *P* < 0.1 (marginally significant), ns, *P* ≥ 0.1 (nonsignificant). See Fig. S5 for corresponding data in the aorta and Fig. S6 for comparisons between IMT-IF and IMT-HC.

At 6 months after starting irradiation, in the vascular endothelial cells (VECs) of the irradiated tunica intima, DAPI negativity (indicating loss of VECs), CD31 (a VEC adhesion molecule) negativity (indicating loss of VECs or CD31) and VECs with subcellular fragments (indicative of cell death) increased (Figs. 2A, 3A–C, S2 and S3A), whereas eNOS (nitric oxide synthase) and VE-cadherin (an adherence junction component) decreased (Figs. 2B,C and 3D,E). This suggests that irradiation causes partial loss of the endothelium involving VEC cell death. In the vascular smooth muscle cells (VSMCs) of the irradiated tunica media, α-SMA (smooth muscle actin) almost remained (Fig. 2A and S2), but VSMCs with subcellular fragments (indicative of cell death) increased (Fig. 3F and S3A). In the irradiated tunica media, there were also increases in TNF-α (a proinflammation marker), CD68 (a macrophage marker), F4/80 (a macrophage marker) and CD3 (a T-cell marker) (Figs. 2D,G and 3G,J and S4). This suggests that irradiation induces VSMC cell death and inflammation. These cells (i.e., VECs and VSMCs) with subcellular fragments were negative with the TUNEL staining (Fig. S3B), cleaved caspase-3 (Fig. S3C) and cleaved N-terminal GSDMD (Fig. S3D), suggesting the involvement of the modes of cell death other than apoptosis and pyroptosis. In the irradiated tunica media, TGF-β1 (a profibrosis marker) and alanine blue stain (a collagen fiber marker) were increased (Figs. 2H, 3K and 4A,C), concomitant with intima media thickening (Figs. 3L and 4D and S6), suggesting that irradiation promotes fibrosis. Radiation-induced changes were qualitatively similar at 6 and 12 months, but its magnitude was overall smaller at 12 months than at 6 months after starting irradiation (Figs. 3 and 4), suggesting recovery post-irradiation.

Altogether, these data show that irradiation leads to vascular damage, inflammation and fibrosis in the carotid artery, all of which are involved in the early stage of atherogenesis.

### Irradiation generates enhancing and sparing effects of dose protraction in the carotid artery

It is evident from Figs. 3 and 4 that the magnitude of radiation-induced changes differs among irradiation regimens and among the carotid endpoints. We therefore conducted the integrative analysis for quantitative inter-regimen comparisons of 14 carotid endpoints (Table 1 and S3–S11). The magnitude of effects among six irradiation regimens was in descending order of “X-rays in 25 fractions ≥ Acute X-rays ≥ γ-rays in 25 fractions > Acute γ-rays > X-rays in 100 fractions > Chronic γ-rays” at 6 months after starting irradiation (Table 1). Likewise, the magnitude of effects among four irradiation regimens was “X-rays in 25 fractions > Acute X-rays > > X-rays in 100 fractions > Chronic γ-rays” at 6 months, “Acute X-rays ≥ X-rays in 25 fractions ≥ X-rays in 100 fractions > Chronic γ-rays” at 12 months, and “X-rays in 25 fractions ≥ Acute X-rays > > X-rays in 100 fractions > Chronic γ-rays” at 6 and 12 months after starting irradiation (Table 1). These data demonstrate that compared with acute irradiation, enhancing effects are produced by intermittent irradiation in 25 fractions, sparing effects being produced by intermittent irradiation in 100 fractions and chronic irradiation (with the latter producing greater sparing effects), and that X-rays are more effective than γ-rays when irradiated acutely or intermittently in 25 fractions.

**Table 1:** Comparisons among irradiation regimens.

At 6 months after starting irradiation (6 irradiation regimens)	
Carotid:	X-rays in 25 Frs ≥ Acute X-rays ≥ γ-rays in 25 Frs > Acute γ-rays > X-rays in 100 Frs > Chronic γ-rays
Aorta:	X-rays in 25 Frs > Acute X-rays > γ-rays in 25 Frs > Acute γ-rays > X-rays in 100 Frs > > Chronic γ-rays
Carotid and aorta:	X-rays in 25 Frs > Acute X-rays > γ-rays in 25 Frs > Acute γ-rays > X-rays in 100 Frs > > Chronic γ-rays

At 6 months after starting irradiation (4 irradiation regimens)	
Carotid:	X-rays in 25 Frs > Acute X-rays > > X-rays in 100 Frs > Chronic γ-rays
Aorta:	X-rays in 25 Frs > Acute X-rays > X-rays in 100 Frs > > Chronic γ-rays
Carotid and aorta:	X-rays in 25 Frs > Acute X-rays > > X-rays in 100 Frs > > Chronic γ-rays

At 12 months after starting irradiation (4 irradiation regimens)	
Carotid:	Acute X-rays ≥ X-rays in 25 Frs ≥ X-rays in 100 Frs > Chronic γ-rays
Aorta:	X-rays in 25 Frs ≥ Acute X-rays > > X-rays in 100 Frs ≥ Chronic γ-rays
Carotid and aorta:	X-rays in 25 Frs = Acute X-rays > > X-rays in 100 Frs > Chronic γ-rays

At 6 and 12 months after starting irradiation (4 irradiation regimens)	
Carotid:	X-rays in 25 Frs ≥ Acute X-rays > > X-rays in 100 Frs > Chronic γ-rays
Aorta:	X-rays in 25 Frs > Acute X-rays > > X-rays in 100 Frs > Chronic γ-rays
Carotid and aorta:	X-rays in 25 Frs > Acute X-rays > > X-rays in 100 Frs > > Chronic γ-rays

Frs, fractions. This table summarizes the results of a series of the Kolmogorov–Smirnov tests to compare effects of various irradiation regimens. See Table S3 for more details.

### The aorta is more responsive to irradiation than the carotid artery

To compare radiation responses of the carotid artery (a potential target tissue for cerebrovascular effects of radiation) and the other large vessel, the corresponding data were analyzed in the aorta (a potential target tissue for cardiovascular effects of radiation). The data in the aorta showed that irradiation induced vascular damage, inflammation and fibrosis, that the magnitude of radiation-induced changes was overall smaller at 12 months than at 6 months after starting irradiation (Fig. S5), and that irradiation caused enhancing and sparing dose protraction effects (Table 1, S3, S12–S19), all of which were reminiscent of the case for the carotid artery. Chronic irradiation tended to be less effective than irradiation in 100 fractions in both the carotid artery and the aorta, and such a tendency was more evident in the aorta than the carotid artery particularly at 6 months after starting irradiation (Table 1). The overall magnitude of effects among irradiation regimens, nevertheless, did not change much when carotid changes, aortic changes or both were taken into account (Table 1 and S3–S27).

Next, comparisons were made between the aorta and the carotid artery (Figs. S11 and S12). Among irradiation regimens at each timepoint, the aorta was more responsive to irradiation, in 8 of 10 comparisons when regimens were considered each separately, and both at 6 and 12 months after starting irradiation when considered altogether (Table S28). Among endpoints tested at each timepoint, and when all regimens were considered together, the aorta exhibited greater radioresponses for multiple endpoints (e.g., TGF-β1 at 6 and 12 months), whilst the carotid artery exhibited greater radioresponses for limited endpoints (i.e., CD3 and CD68 at 6 months) (Table S29). These data show that the aorta is more radioresponsive than the carotid artery.

### Radiation-induced changes are similar to age-related changes

To compare radiation-induced changes and age-related changes, the carotid artery and the aorta obtained from nonirradiated young (age 8 weeks) or aged (age 104 weeks) mice were evaluated for 14 endpoints, along with those from sham-irradiated mice (Figs. S13 and S14). Various endpoints altered as a function of age (e.g., quadratic for VE-cadherin, power for TNF-α, exponential for TGF-β1, and linear for IMT-HC in the carotid artery, logistic for CD3 in the aorta) (Table S30).

All endpoints (14 each for the carotid artery and the aorta) showed significant changes when nonirradiated young and aged mice were compared: without exception, such changes in aged mice were all in the same direction (in terms of increases or decreases) as in irradiated mice, although the level of changes was different (Figs. 3 and 4 and S5). These data demonstrate that changes observed in irradiated mice are qualitatively similar to those in aged mice, albeit quantitatively different.

## Discussion

Here, we discuss our major findings, specifically focusing on sparing and enhancing effects of dose protraction, greater effects of X-rays than γ-rays, greater radioresponses of the aorta than the carotid artery, similarity of radiation-induced and age-related changes, and the strengths and limitations of the study.

First, we showed that irradiation causes sparing and enhancing effects of dose protraction in the carotid artery and the aorta (e.g., “25 fractions > acute > > 100 fractions > > chronic”, Table 1). This suggests that dose protraction effects are not a simple function of dose rate and the number of fractions, for which a minimum of three inflection points should exist: i.e., the first point lying between 2 and 25 fractions where the direction of the tissue response changes over from no dose protraction effect to an enhancing effect, the second between 25 and 100 fractions from an enhancing effect to no dose protraction effect, and the last between 25 and 100 fractions from no dose protraction effect to a sparing effect. In cases where the level of damage resulting from a repair of pre-existing damage produced by preceding irradiation equals the level of newly formed damage produced by subsequent irradiation, dose protraction does not change the total level of residual damage within the tissue: therefore, there is no dose protraction effect. If damage repair outweighs damage production, then a sparing effect occurs. The observation that intermittent irradiation in 100 fractions elicited a sparing effect, chronic irradiation at 1.4 mGy/h eliciting a greater sparing effect, despite the same irradiation period (i.e., 153 days), underlines the importance of temporal dose distribution in radiation effects. Whether chronic irradiation at lower dose rate (i.e., < 1.4 mGy/h) is even less effective remains unclear. Sparing dose protraction effects coincide with a long-standing radiobiological tenet^22^, and several epidemiological studies are supportive^23,24^. On the other hand, if damage production outweighs damage repair, then an enhancing effect occurs, as observed following intermittent irradiation in 25 fractions that are frequent in radiotherapy (e.g., for cancer involving thoracic and neck regions). Enhancing dose protraction effects reported in VECs in vitro^25^, in kidneys and for DCS mortality in vivo^26–28^, and in the Canadian cohort^29^ lend support to our observations in large vessels. The underlying mechanisms are unknown^6^, but possible mechanisms involve greater effects per unit radiation dose at lower dose, and production of greater damage posed by interactions between pre-existing damage and newly formed damage. The former possibility is supported epidemiologically by the studies of atomic bomb survivors and other cohorts^4,30,31^. Complicated patterns of dose protraction effects have also recently been observed in the meta-analysis of relevant epidemiological studies^4^, and its implications for radiation protection await further discussion^32,33^.

Second, we found that X-rays (260 kVp) are more effective than ^137^Cs γ-rays (662 keV) when irradiated acutely or in 25 fractions, and particularly, increases in TGF-β1 and F4/80 were evident after acute X-ray, but not γ-ray irradiation (Fig. 3I,K and S5I,K). Such greater effects of photons (i.e., X-rays and γ-rays) at lower energy are consistent with in vitro evidence^34,35^ and have been attributed to formation of more irreparable, complex clustered DNA damage^36^. Contrarily, there has heretofore been no in vivo study directly comparing normal tissue effects of photons with different energy^36^. Due to lower energy of photons, the actual effects per unit radiation absorbed dose can be greater than observed, particularly for the aorta: this is because given the same entrance skin dose delivered vertically (i.e., 5 Gy in this study), depth dose (e.g., at the aorta) is lower for X-rays than γ-rays, and such differences are more obvious in tissues/organs situated farther away (more deeply) from the skin. Given that the carotid artery is located shallower (closer to the skin) than the aorta, dose to the carotid artery should receive higher dose than the aorta particularly for X-rays, so the actual effects for the carotid artery per unit radiation absorbed dose can be smaller than observed, compared with those for the aorta: this is further supportive of the possibility that the aorta is more radioresponsive than the carotid artery. More studies with various types of radiation with differing quality would be required, as patients and workers receive exposure to photons with wide-ranging energy (three orders of magnitude from keV or kV through MeV or MV) and other types of radiation (e.g., protons and heavy ions in radiotherapy patients, neutrons in nuclear workers, protons, heavy ions and neutrons in astronauts and other space travelers)^37^.

Third, we showed that the aorta is more radioresponsive than the carotid artery. Irradiation has been reported to accelerate the development of atherosclerosis in the carotid artery and the aorta of ApoE^–/–^ mice^9,38,39^. In this regard, the carotid artery and the aorta in any of irradiated, sham- or nonirradiated mice used in this study were all negative with Oil Red O (Fig. 4B), suggesting that irradiation and aging produce no mature atherosclerotic lesion in wildtype mice. It hence remains unclear whether greater radioresponsiveness evaluated in this study as greater prelesional changes can be translated into greater lesional changes, epidemiological evidence of higher radiation risk per unit dose for cerebrovascular disease than ischemic heart diseases^4^ being taken into account. Nevertheless, our finding adds to existing knowledge on the potential difference in radioresponses of the blood vessels which were previously reported only in ApoE^–/–^ mice (comparing the carotid artery with the brachiocephalic artery)^10^. For radiation risk management, further studies would be needed to identify the radiosensitive targets within the circulatory system that underpin radiation-induced DCS, first at the tissue/organ level (e.g., lens epithelium for the eye dose and the epidermis for the skin dose) and then at the cellular level (e.g., lens epithelial cells for the eye dose and basal cells for the skin dose)^3^.

Fourth, we found that in the carotid artery and the aorta, radiation-induced changes and age-related changes were quantitatively different but qualitatively similar. For example, CD3 and CD68 were the endpoints for which the carotid artery exhibited greater responses than the aorta, not only in irradiated mice (Table S29), but also in aged mice (Fig. S12). This raises the possibility that irradiation accelerates development of the age-related changes in the circulatory system, as proposed for some types of radiation-induced cataracts and cancer^40,41^. This is supported by the in vivo observations of increased activity of senescence-associated β-galactosidase in the rat carotid artery and the murine aorta following irradiation^42,43^. The mechanisms behind radiation-induced atherogenesis and age-related atherogenesis are not necessarily identical^10^. Nevertheless, if accelerated aging contributes, at least in part, to radiation-induced atherogenesis, then senolytic and senomorphic approaches to eliminating or modulating senescent cells^44,45^ may be useful for its therapeutic interventions.

Last, our study had strengths on the one hand. In particular, comparisons were based on rigorous statistical analyses of the robust datasets that consisted of 14 endpoints each in the carotid artery and the aorta obtained from 22 groups of mice, with 12 sham- or nonirradiated control groups included. On the other hand, this study had limitations as well. Besides radiation quality, several differences existed among irradiation regimens: e.g., husbandry conditions (different diets and beddings used at two institutes as detailed in Supplemental Methods), cages rotated during X- but not γ-irradiation, irradiation geometries (X-irradiation unidirectionally in the top-down direction, acute and fractionated γ-rays bidirectionally both in the top-down and bottom-up directions, chronic γ-rays isotropically), and X- and chronic γ-irradiation conducted at CRIEPI (and then mice shipped to Hiroshima University) vs. acute and fractionated γ-irradiation at Hiroshima University. Taken together, there were lack of irradiation with acute γ-rays and γ-rays in 25 fractions for the 12 months post-irradiation timepoint, lack of γ-rays in 100 fractions and chronic X-rays (technically infeasible though), inconsistent duration of irradiation (42 days for 25 fractions vs. 153 days for 100 fractions and chronic irradiation), lack of acute irradiation at age 14 weeks (when delivery of 25 fractions ends) and 30 weeks (when delivery of 100 fractions and chronic irradiation end), and lack of multiple dosepoints for total dose (i.e., a single dosepoint of 5 Gy used in this study). We employed total body irradiation, so radiation effects observed in the carotid artery and the aorta should result from effects arising not only in these vessels, but also in other potential target tissues/organs (e.g., heart, kidneys). Targeted local irradiation (e.g., neck irradiation for the carotid artery) may be more preferable to identify radiosensitive target tissues/organs, but is impractical for highly fractionated irradiation and technically infeasible for chronic irradiation.

## Conclusions

We demonstrated that irradiation causes vascular damage, inflammation and fibrosis in the carotid artery of wildtype mice. These prelesional changes have all been implicated in the early stage of atherogenesis, although no mice actually developed mature atherosclerotic lesions. The integrative analysis for a series of prelesional endpoints in the carotid artery revealed that effects vary with radiation quality, and that dose protraction effects are not a simple function of dose rate and the number of fractions. Qualitatively, radiation effects in the aorta were similar to those in the carotid artery, but quantitatively, the aorta was more radioresponsive than the carotid artery. Moreover, radiation-induced changes in the carotid artery and the aorta were qualitatively similar to age-related changes. Further studies would be needed to better understand manifestations and mechanisms of radiation-induced DCS, which along with this study, contribute to ongoing discussions in ICRP and elsewhere to develop international recommendations for management of radiation risk and optimization of radiation protection.

## Electronic supplementary material

Below is the link to the electronic supplementary material.

Supplementary Material 1

## Data Availability

All data generated or analyzed during this study are included in this published article and its supplementary information files.

## References

[1] Emami, B. et al. Tolerance of normal tissue to therapeutic irradiation. *Int. J. Radiat. Oncol. Biol. Phys.* **21**, 109–122. 10.1016/0360-3016(91)90171-y (1991). 2032882 10.1016/0360-3016(91)90171-y

[2] Shimizu, Y. et al. Radiation exposure and circulatory disease risk: Hiroshima and Nagasaki atomic bomb survivor data, 1950–2003. *BMJ* **340**, b5349. 10.1136/bmj.b5349 (2010). 20075151 10.1136/bmj.b5349 PMC2806940

[3] ICRP. ICRP Publication 118. ICRP statement on tissue reactions and early and late effects of radiation in normal tissues and organs: Threshold doses for tissue reactions in a radiation protection context. *Ann. ICRP* **41**(1–2), 1–322. 10.1016/j.icrp.2012.02.001 (2012). 10.1016/j.icrp.2012.02.001 22925378

[4] Little, M. P. et al. Ionising radiation and cardiovascular disease: Systematic review and meta-analysis. *BMJ* **380**, e072924. 10.1136/bmj-2022-072924 (2023). 36889791 10.1136/bmj-2022-072924 PMC10535030

[5] Benali, K. et al. One-year mortality and causes of death after stereotactic radiation therapy for refractory ventricular arrhythmias: A systematic review and pooled analysis. *Trends Cardiovasc. Med.* **34**, 488–496. 10.1016/j.tcm.2023.12.008 (2024). 38191005 10.1016/j.tcm.2023.12.008

[6] Tapio, S. et al. Ionizing radiation-induced circulatory and metabolic diseases. *Environ. Int.* **146**, 106235. 10.1016/j.envint.2020.106235 (2021). 33157375 10.1016/j.envint.2020.106235 PMC10686049

[7] Little, M. P. et al. Analysis of dose response for circulatory disease after radiotherapy for benign disease. *Int. J. Radiat. Oncol. Biol. Phys.* **84**, 1101–1109. 10.1016/j.ijrobp.2012.01.053 (2012). 22494591 10.1016/j.ijrobp.2012.01.053 PMC3396750

[8] Leboucher, A. et al. Head and neck radiotherapy-induced carotid toxicity: Pathophysiological concepts and clinical syndromes. *Oral Oncol.* **129**, 105868. 10.1016/j.oraloncology.2022.105868 (2022). 35512488 10.1016/j.oraloncology.2022.105868

[9] Hoving, S. et al. Single-dose and fractionated irradiation promote initiation and progression of atherosclerosis and induce an inflammatory plaque phenotype in ApoE^–/–^ mice. *Int. J. Radiat. Oncol. Biol. Phys.* **71**, 848–857. 10.1016/j.ijrobp.2008.02.031 (2008). 18514779 10.1016/j.ijrobp.2008.02.031

[10] Hoving, S. et al. NO-donating aspirin and aspirin partially inhibit age-related atherosclerosis but not radiation-induced atherosclerosis in ApoE null mice. *PLoS One*. **5**, e12874. 10.1371/journal.pone.0012874 (2010). 20877628 10.1371/journal.pone.0012874 PMC2943480

[11] Ebrahimian, T. G. et al. Chronic exposure to external low-dose gamma radiation induces an increase in anti-inflammatory and anti-oxidative parameters resulting in atherosclerotic plaque size reduction in ApoE^–/–^ mice. *Radiat. Res.* **189**, 187–196. 10.1667/RR14823.1 (2018). 29227739 10.1667/RR14823.1

[12] Takahashi, N. et al. Association between low doses of ionizing radiation, administered acutely or chronically, and time to onset of stroke in a rat model. *J. Radiat. Res.* **61**, 666–673. 10.1093/jrr/rraa050 (2020). 32748938 10.1093/jrr/rraa050 PMC7482173

[13] Hamada, N. et al. Inverse dose protraction effects of low-LET radiation: Evidence and significance. *Mutat. Res.* **795**, 108531. 10.1016/j.mrrev.2025.108531 (2025). 10.1016/j.mrrev.2025.108531 PMC12124966 39814314

[14] Hamada, N. et al. Inverse dose protraction effects of high-LET radiation: Evidence and significance. *Mutat. Res.* **795**, 108530. 10.1016/j.mrrev.2025.108530 (2025). 10.1016/j.mrrev.2025.108530 PMC12124982 39818312

[15] Wesolowska, P. E. et al. Characterization of three solid state dosimetry systems for use in high energy photon dosimetry audits in radiotherapy. *Radiat. Meas.* **106**, 556–562. 10.1016/j.radmeas.2017.04.017 (2017).

[16] Hoshi, Y. et al. Application of a newly developed photoluminescence glass dosimeter for measuring the absorbed dose in individual mice exposed to low-dose rate ^137^Cs γ-rays. *J. Radiat. Res.* **41**, 129–137. 10.1269/jrr.41.129 (2000). 11037580 10.1269/jrr.41.129

[17] Sasatani, M. et al. Morphology dynamics in intestinal crypt during postnatal development affect age-dependent susceptibility to radiation-induced intestinal tumorigenesis in Apc^Min/+^ mice: Possible mechanisms of radiation tumorigenesis. *Carcinogenesis* **44**, 105–118. 10.1093/carcin/bgac100 (2023). 36546734 10.1093/carcin/bgac100 PMC10183640

[18] Hamada, N. et al. Ionizing irradiation induces vascular damage in the aorta of wild-type mice. *Cancers* **12**, 3030. 10.3390/cancers12103030 (2020). 33081026 10.3390/cancers12103030 PMC7603246

[19] Hamada, N. et al. Vascular damage in the aorta of wild-type mice exposed to ionizing radiation: Sparing and enhancing effects of dose Protraction. *Cancers* **13**, 5344. 10.3390/cancers13215344 (2021). 34771507 10.3390/cancers13215344 PMC8582417

[20] Hamada, N. et al. Temporal changes in sparing and enhancing dose protraction effects of ionizing irradiation for aortic damage in wild-type mice. *Cancers* **14**, 3319. 10.3390/cancers14143319 (2022). 35884380 10.3390/cancers14143319 PMC9321929

[21] Hamada, N. et al. Sparing and enhancing dose protraction effects for radiation damage to the aorta of wild-type mice. *Int. J. Radiat. Biol.* **100**, 37–45. 10.1080/09553002.2023.2242939 (2024). 37523500 10.1080/09553002.2023.2242939

[22] Rühm, W. et al. Dose and dose-rate effects of ionizing radiation: A discussion in the light of radiological protection. *Radiat. Environ. Biophys.* **54**, 379–401. 10.1007/s00411-015-0613-6 (2015). 26343037 10.1007/s00411-015-0613-6

[23] Sasaki, M., Kudo, S. & Furuta, H. Effect of radiation dose rate on circulatory disease mortality among nuclear workers: Reanalysis of Hanford data. *Health Phys.* **119**, 280–288. 10.1097/HP.0000000000001230 (2020). 32205716 10.1097/HP.0000000000001230

[24] Azizova, T. V., Grigoryeva, E. S. & Hamada, N. Dose rate effect on mortality from ischemic heart disease in the cohort of Russian Mayak Production Association workers. *Sci. Rep*. **13**, 1926. 10.1038/s41598-023-28954-w (2023). 10.1038/s41598-023-28954-w PMC9895442 36732598

[25] Cervelli, T. et al. Effects of single and fractionated low-dose irradiation on vascular endothelial cells. *Atherosclerosis* **235**, 510–518. 10.1016/j.atherosclerosis.2014.05.932 (2014). 24953491 10.1016/j.atherosclerosis.2014.05.932

[26] Seol, M. A. et al. Prolonged expression of senescence markers in mice exposed to γ-irradiation. *J. Vet. Sci.* **13**, 331–338. 10.4142/jvs.2012.13.4.331 (2012). 23271173 10.4142/jvs.2012.13.4.331 PMC3539117

[27] Hoel, D. G. & Carnes, B. A. Cardiovascular effects of fission neutron or ^60^Co γ exposure in the B6CF_1_ mouse. *Int. J. Radiat. Biol.* **93**, 563–568. 10.1080/09553002.2017.1286051 (2017). 28112567 10.1080/09553002.2017.1286051

[28] Tran, V. & Little, M. P. Dose and dose rate extrapolation factors for malignant and non-malignant health endpoints after exposure to γ and neutron radiation. *Radiat. Environ. Biophys.* **56**, 299–328. 10.1007/s00411-017-0707-4 (2017). 28939964 10.1007/s00411-017-0707-4

[29] Zablotska, L. B., Little, M. P. & Cornett, R. J. Potential increased risk of ischemic heart disease mortality with significant dose fractionation in the Canadian fluoroscopy cohort study. *Am. J. Epidemiol.* **179**, 120–131. 10.1093/aje/kwt244 (2014). 24145888 10.1093/aje/kwt244 PMC3864716

[30] Little, M. P. et al. Lifetime mortality risk from cancer and circulatory disease predicted from the Japanese atomic bomb survivor life span study data taking account of dose measurement error. *Radiat. Res.* **194**, 259–276. 10.1667/RR15571.1 (2020). 32942303 10.1667/RR15571.1 PMC7646983

[31] Little, M. P. & Hamada, N. Low-dose extrapolation factors implied by mortality and incidence data from the Japanese atomic bomb survivor life span study data. *Radiat. Res.* **198**, 582–589. 10.1667/RADE-22-00108.1 (2022). 36161867 10.1667/RADE-22-00108.1 PMC9797579

[32] Hamada, N. Noncancer effects of ionizing radiation exposure on the eye, the circulatory system and beyond: Developments made since the 2011 ICRP statement on tissue reactions. *Radiat. Res.* **200**, 188–216. 10.1667/RADE-23-00030.1 (2023). 37410098 10.1667/RADE-23-00030.1

[33] Zablotska, L. B., Little, M. P. & Hamada, N. Revisiting an inverse dose-fractionation effect of ionizing radiation exposure for ischemic heart disease: Insights from recent studies. *Radiat. Res.* **202**, 80–86. 10.1667/RADE-23-00230.1 (2024). 38772552 10.1667/RADE-00230.1 PMC11260496

[34] Schmid, E., Regulla, D., Kramer, H. M. & Harder, D. The effect of 29 kV X rays on the dose response of chromosome aberrations in human lymphocytes. *Radiat. Res.* **158**, 771–777. 10.1667/0033-7587(2002)158[0771:teokxr]2.0.co;2 (2002). 12452780 10.1667/0033-7587(2002)158[0771:teokxr]2.0.co;2

[35] Korns, J. et al. Varied photon radiation sources produce differences in cellular response. *Radiat. Res.* **199**, 422–428. 10.1667/RADE-22-00210.1 (2023). 37039678 10.1667/RADE-22-00210.1 PMC12306632

[36] NCRP. Evaluation of the relative effectiveness of low-energy photons and electrons in inducing cancer in humans. NCRP Report No.181. Maryland, NCRP. (2018). 10.1097/HP.0000000000001011 30889098

[37] Saigusa, Y. et al. Biological effects of high-LET irradiation on the circulatory system. *Int. J. Radiat. Biol.* 101, in press. 10.1080/09553002.2025.2470947 (2025). 10.1080/09553002.2025.2470947 PMC12011529 40063776

[38] Stewart, F. A. et al. Ionizing radiation accelerates the development of atherosclerotic lesions in ApoE^–/–^ mice and predisposes to an inflammatory plaque phenotype prone to hemorrhage. *Am. J. Pathol.* **168**, 649–658. 10.2353/ajpath.2006.050409 (2006). 16436678 10.2353/ajpath.2006.050409 PMC1606487

[39] Mancuso, M. et al. Acceleration of atherogenesis in ApoE^–/–^ mice exposed to acute or low-dose-rate ionizing radiation. *Oncotarget* **6**, 31263–31271. 10.18632/oncotarget.5075 (2015). 26359350 10.18632/oncotarget.5075 PMC4741603

[40] Uwineza, A. et al. Cataractogenic load: A concept to study the contribution of ionizing radiation to accelerated aging in the eye lens. *Mutat. Res.* **779**, 68–81. 10.1016/j.mrrev.2019.02.004 (2019). 10.1016/j.mrrev.2019.02.004 31097153

[41] Nakamura, N. Reasons why the Idea that radiation exposures induce cancer needs to be revisited. *Int. J. Radiat. Biol.* **100**, 824–833. 10.1080/09553002.2024.2338516 (2024). 38647670 10.1080/09553002.2024.2338516

[42] Park, J. W. et al. Metformin alleviates ionizing radiation-induced senescence by restoring BARD1-mediated DNA repair in human aortic endothelial cells. *Exp. Gerontol.* **160**, 111706. 10.1016/j.exger.2022.111706 (2022). 35085707 10.1016/j.exger.2022.111706

[43] Zheng, X. et al. Ionizing radiation induces vascular smooth muscle cell senescence through activating NF-κB/CTCF/p16 pathway. *Biochim. Biophys. Acta Mol. Basis Dis.* **1870**, 166994. 10.1016/j.bbadis.2023.166994 (2024). 38141838 10.1016/j.bbadis.2023.166994

[44] Suda, M. et al. Senolytic vaccination improves normal and pathological age-related phenotypes and increases lifespan in progeroid mice. *Nat. Aging*. **1**, 1117–1126. 10.1038/s43587-021-00151-2 (2021). 37117524 10.1038/s43587-021-00151-2

[45] Herman, A. B. et al. DPP4 Inhibition impairs senohemostasis to improve plaque stability in atherosclerotic mice. *J. Clin. Invest.* **133**, e165933. 10.1172/JCI165933 (2023). 37097759 10.1172/JCI165933 PMC10266795

