# Supplementary material for: Responses of the carotid artery to acute, fractionated or chronic ionizing irradiation, and differences from the aorta
Source: Sci Rep. 2025 Mar 5;15:7712. doi: 10.1038/s41598-025-92710-5 (PMC11883035; doi:10.1038/s41598-025-92710-5)
Supplement: Supplementary file 1 — Supplementary Material 1 [file 41598_2025_92710_MOESM1_ESM.pdf]

Supplementary Information

Responses of the carotid artery to acute, fractionated or chronic ionizing irradiation, and differences from the aorta

Nobuyuki Hamada, RT, PhD; Ki-ichiro Kawano, PhD; Seiko Hirota, PhD; Farina Mohamad Yusoff, MBBS, PhD; Takaharu Nomura, PhD; Yusuke Saito; Ayumu Nakashima, MD, PhD; Shinji Yoshinaga, PhD; Yukihito Higashi, MD, PhD

Table of Contents

Supplementary Methods	Pages 2–6
Supplementary Tables S1–S30	Pages 7–28
Supplementary Figures S1–S14	Pages 29–56

Supplementary Methods

Shipping of Mice, Irradiation and Tissue Sampling

For all experiments, male C57BL/6J mice were used, and maintained under a 12 h light/dark cycle (light onset at 8 am) with ad libitum access to food and water. All mice were fed a normal-fat diet: CE-2 (~3.4 kcal/g, ~4.6% of the calorie from crude fat, CLEA Japan) used at Central Research Institute of Electric Power Industry (CRIEPI, Tokyo, Japan) and MF (~3.5 kcal/g, ~13% of the calorie from crude fat, Oriental Yeast, Japan) used at Hiroshima University (Hiroshima, Japan). The beddings used were paper chips (PaperClean, Japan SLC, Shizuoka, Japan) at CRIEPI and wood chips (Beta Chip, Northeastern Products Corp, Warrensburg, NY, USA) at Hiroshima University. Figure S1 depicts detailed experimental timelines for 22 groups (10 irradiated, 10 sham-irradiated and 2 nonirradiated).

For the "acute X-rays", "X-rays in 25 fractions", "X-rays in 100 fractions" and "chronic γ-rays" regimens, 7-week-old mice were shipped by car (~48 km, ~1.2 h) from Jackson Laboratory Japan (Kanagawa, Japan) to CRIEPI, and acclimated for a week prior to irradiation at the age of 8 weeks. For the "acute X-rays", "X-rays in 25 fractions" and "X-rays in 100 fractions" regimens, unanesthetized 8-week-old mice were placed in a 12-compartment pie cage (Natsume Seisakusho, Japan) just before irradiation, and were irradiated at CRIEPI with 5 Gy of X-rays (260 kVp and 4.5 mA with a 0.5 mm Al and 0.3 mm Cu filter, a source-surface distance of 52.7 cm, 0.5 Gy/min) from a Faxitron MultiRad350 irradiator, either acutely as a single dose (in ~10 min), intermittently in 25 daily fractions (0.2 Gy/fraction spread over 42 days) or in 100 daily fractions (0.2 Gy/fraction over 153 days). Mice received X-rays vertically (unidirectionally in the top-down direction). For the "chronic γ-rays" regimen, unanesthetized 8-week-old mice were placed in a conventional cage just before irradiation and were continuously irradiated at CRIEPI with 5 Gy of ^{137}Cs γ-rays (a source-surface distance of 371 cm, 1.4 mGy/h over 153 days) except for a time needed for husbandry (67.6 h in 153 days, 3.1 h/week). Mice received γ-rays isotropically. Irradiated or sham-irradiated mice for the "acute X-rays", "X-rays in 25 fractions", "X-rays in 100 fractions" and "chronic γ-rays" regimens were shipped by car and air (~717 km, 6.4 ± 0.5 h) from CRIEPI to Hiroshima University, followed by a two-week quarantine period.

For the "acute γ-rays" and "γ-rays in 25 fractions" regimens, mice aged 7 weeks were shipped by car (~424 km, ~5.8 h) from Jackson Laboratory Japan (Shiga, Japan) to Hiroshima University, and acclimated for a week prior to irradiation at age 8 weeks. Unanesthetized 8-week-old mice were placed in a pie cage just before irradiation and

were irradiated at Hiroshima University with 5 Gy of ^{137}Cs γ-rays (0.5 Gy/min) from Gammacell 40 Exactor, either acutely as a single dose (in 10 min) or intermittently in 25 daily fractions (0.2 Gy/fraction over 42 days). Mice received γ-rays vertically from two sources (i.e., bidirectionally in both the top-down and bottom-up directions).

All control mice were sham-irradiated in parallel with the test mice. For five (all but "chronic γ-rays") regimens, mice put in a pie cage were placed into an irradiator for the same time needed to complete irradiation, but were not actually irradiated. The pie cage was rotated on a turntable during X- or its sham-irradiation, but not during γ- or its sham-irradiation. For the "chronic γ-rays" regimen, mice put in a conventional cage were placed outside the irradiation field but within the irradiation facility.

For nonirradiated "young" and "aged" control groups, mice at age 5 and 101 weeks, respectively, were shipped by car (~778 km, ~10 h) from Jackson Laboratory Japan (Kanagawa, Japan) to Hiroshima University and were acclimated for three weeks before sampling at age 8 and 104 weeks.

At age 8 weeks ("young"), 34 weeks (at 6 months after starting irradiation), 60 weeks (at 12 months after starting irradiation) or 104 weeks ("aged"), mice were anesthetized with isoflurane, perfused transcardially with phosphate-buffered saline, and underwent tissue sampling. The right common carotid artery and the caudal half of the descending thoracic aorta were embedded in optimal cutting temperature (OCT) compound (Sakura Finetek, Japan) and snap-frozen (first with the cold isopentane and then liquid nitrogen), and stored at −80°C.

All animal experiments were approved by the Animal Research and Ethics Committee of CRIEPI (approval number 2016–08) and the Institutional Animal Care and Use Committee of Hiroshima University (approval number A16–139), and performed in compliance with the Japanese guidelines of animal care.

Except for two nonirradiated control groups (i.e., "young" and "aged" mice), animals purchased at age 7 weeks were randomly allocated to each of the 20 irradiated or sham-irradiated groups. For the two nonirradiated control groups, animals purchased at age 5 and 101 weeks were all used for sampling at age 8 weeks (as "young") and 104 weeks (as "aged"), respectively. The sample size in each study group for the full-scale experiments was determined based on the results of the preliminary experiments performed with 3 mice/group. All mice in the same group (8–10 mice/group) was housed in a single conventional cage, except for a pie cage during acute or fractionated irradiation or corresponding sham-irradiation, and except for the "Acute X-rays, 12 months" group for which 24 mice were housed in three conventional cages. The data for post-irradiation changes in survival, body weight, heart weight, kidney weight and echocardiographic

changes have previously been reported for 20 irradiated or sham-irradiated groups [S1–S3], among which a significant decrease in survival was observed only in one group ("Acute X-rays, 12 months") [S3]. For randomization, irradiation and sampling, two authors (KIK and YS) knew the group allocation for two irradiation regimens ("acute γ-rays" and "γ-rays in 25 fractions" for which irradiation and sampling were carried out at Hiroshima University), and two authors (NH and TN) knew the group allocation for other irradiation regimens (irradiation and sampling were carried out at CRIEPI and Hiroshima University, respectively). Two authors (NH and KIK) had full access to all of the samples and the staining images. Three authors (NH, SH and SY) had full access to all of the numerical data. The analysis was made for the data obtained from all available samples in the full-scale experiments with no exclusions made, and did not include any data from the preliminary experiments.

References

[S1] N. Hamada, K-.I. Kawano, T. Nomura, K. Furukawa, F.M. Yusoff, T. Maruhashi, et al., Vascular damage in the aorta of wild-type mice exposed to ionizing radiation: Sparing and enhancing effects of dose protraction, Cancers 13 (2021) 5344, https://doi.org/10.3390/cancers13215344

[S2] N. Hamada, K-.I. Kawano, T. Nomura, K. Furukawa, F.M. Yusoff, T. Maruhashi, et al., Temporal changes in sparing and enhancing dose protraction effects of ionizing irradiation for aortic damage in wild-type mice, Cancers 14 (2022) 3319, https://doi.org/10.3390/cancers14143319

[S3] N. Hamada, K-.I. Kawano, S. Hirota, Y. Saito, F.M. Yusoff, T. Maruhashi, et al., Sparing and enhancing dose protraction effects for radiation damage to the aorta of wild-type mice, Int. J. Radiat. Biol. 100 (2024) 37–45, https://doi.org/10.1080/09553002.2023.2242939

Quantitative Analysis of Images from Immunofluorescence and Histochemistry Staining

Images of the carotid artery and the aorta were captured using a Keyence BZ-9000 fluorescence microscope (Keyence, Japan), and processed with Keyence BZ-X Analyzer. Tiled images for the carotid artery (including those shown in Figures 2, 4 and S3) were created from images taken at 600 × magnification (60 × objective, 10 × eyepiece) for all markers. Tiled images for the aorta were created from images taken at 600 × magnification (60 × objective, 10 × eyepiece) for endothelial nitric oxide synthase (eNOS) and vascular endothelial cadherin (VE-cadherin), or from those at 200 ×

magnification (20 × objective, 10 × eyepiece) for other markers: i.e., tumor necrosis factor α (TNF-α), cluster of differentiation 68 (CD68), F4/80, CD3, transforming growth factor β1 (TGF-β1) or α-smooth muscle actin (α-SMA). For each mouse, 14 endpoints were quantified as below.

- As endpoints for partial loss of the vascular endothelium, CD31 negativity (defined as loss of green signals in the tunica intima) and 4',6-diamidino-2-phenylindole (DAPI) negativity (loss of blue signals in the tunica intima) were evaluated in 7 tiled immunofluorescence images (stained for CD31 in combination with either eNOS, VE-cadherin, TNF-α, CD68, F4/80, CD3 or TGF-β1), and expressed as a percentage.
- For eNOS, intensity of red signals in the tunica intima was measured in randomly selected five areas (90 μm × 30 μm for the carotid artery, 150 μm × 50 μm for the aorta) in one immunofluorescence image taken at 600 × magnification, and expressed in arbitrary units (AU)/μm^2.
- For VE-cadherin, the number of red dots in the tunica intima was counted in four immunofluorescence images taken at 600 × magnification, and expressed in dots/field.
- For vascular endothelial cells (VECs) and vascular smooth muscle cells (VSMCs) with subcellular fragments, all cells in the tunica intima and in the tunica media, respectively, were counted at 600 × magnification in two sections for immunofluorescence (stained for CD31 simultaneously with either eNOS or VE-cadherin), and expressed as a percentage. The total number of cells counted in each mouse was 56–388 VECs and 114–1056 VSMCs for the carotid artery and 120–387 VECs and 306–1670 VSMCs for the aorta.
- For TNF-α and TGF-β1, intensity of red signals in the tunica media was measured in randomly selected five areas (30 μm × 30 μm for the carotid artery, 50 μm × 50 μm for the aorta) in one immunofluorescence image taken at 200 × magnification, and expressed in AU/μm^2.
- For CD68, F4/80 and CD3, the number of red dots in the tunica media was counted in four immunofluorescence images taken at 200 × magnification, and expressed in dots/field.
- For IMT-IF, the maximal intima-media thickness (IMT) was measured at 200 × magnification in seven sections for immunofluorescence (stained for CD31 in combination with either eNOS, VE-cadherin, TNF-α, CD68, F4/80, CD3 or TGF-β1), and expressed in μm.
- For intensity of aniline blue per unit wall area, intensity of blue signals in the tunica media and wall area were measured in one section for Masson's trichrome staining,

and expressed in AU/μm^2.

- For IMT-HC, the maximal IMT was measured at 200 × magnification in one section for Masson's trichrome staining, and expressed in μm.

Integrative Analysis with the Kolmogorov–Smirnov Test

To compare differences in effects of various irradiation regimens, the integrative analysis was performed with the Kolmogorov–Smirnov test (Tables 1 and S3–S27). For a comparison between groups "A" (e.g., acute X-rays) and "B" (e.g., acute γ-rays), alternative hypotheses "|A| > |B|" and "|A| < |B|" were tested for multiple endpoints (≤14 each for carotid and aortic endpoints), vis-à-vis the null hypothesis that the absolute values of the radiation effects between the given comparison groups are equal for all endpoints (|A| = |B|). For each inter-regimen comparison, a difference in effects was judged based on *P* values (of the one-sided Kolmogorov–Smirnov goodness-of-fit test for the null hypothesis that the distribution of the *P* values from the individual tests is the standard uniform, which is true with no difference in radiation effects for all endpoints between the given comparison groups) according to the criteria for |A| > |B| : >> ($P < 1 \times 10^{-5}$), > ($1 \times 10^{-5} \leq P < 1 \times 10^{-1}$), ≥ ($1 \times 10^{-1} \leq P < 3 \times 10^{-1}$) and = ($P \geq 3 \times 10^{-1}$). The overall difference among six irradiation regimens at 6 months after starting irradiation were judged based on differences in each of 15 inter-regimen comparisons. The overall difference among four irradiation regimens at 6 and/or 12 months after starting irradiation were judged based on differences in each of six inter-regimen comparisons.

The integrative analyses were carried out, first for the carotid artery (Tables S4–S11), second for the aorta (Tables S12–S19), and the last for the carotid artery and the aorta together (Tables S20–S27). For each vessel, the integrative analyses were performed, first at 6 months (Tables S4–S7, S12–S15 and S20–S23), second at 12 months (Tables S8, S9, S16, S17, S24 and S25), and the last at 6 and 12 months together after starting irradiation (Tables S10, S11, S18, S19, S26 and S27). At each timepoint, the integrative analyses were made, first for all endpoints tested (14 endpoints each for the carotid artery and the aorta) (Tables S4, S6, S8, S10, S12, S14, S16, S18, S20, S22, S24 and S26), and then only for endpoints that showed significant differences at least in one pair among 15 pairs for six irradiation regimens (Tables S5, S13 and S21) or among six pairs for four irradiation regimens (Tables S7, S9, S11, S15, S17, S19, S23, S25 and S27). The overall differences among irradiation regimens shown in Tables S4–S27 are listed altogether in Table S3, which is further summarized in Table 1.

Supplementary Tables

Table S1. Literature Reporting Radiation Effects in the Murine Carotid Artery

References[a]	Radiation type, irradiated area	Dose, irradiation regimen (dose rate)	Mouse strain, sex	Age at irradiation	Time assayed after starting irradiation	Non-radiation treatments
Stewart et al. 2006 [S4]	250 kV X-rays, neck	14 Gy, single acute[b]	ApoE$^{-/-}$ [c], both sexes	10–14 weeks	13–34 weeks	–
Hoving et al. 2008 [S5]	250 kV X-rays, neck	8–14 Gy, single acute[b]	ApoE$^{-/-}$ [c], female	10–15 weeks	1–34 weeks	–
		40 Gy, 20 Frs (2 Gy/Fr[b], 5 Frs/week) spread over 4 weeks	ApoE$^{-/-}$ [c], male	10–16 weeks	5–34 weeks	–
Lawton et al. 2008 [S6]	150 kVp X-rays, neck	25–200 Gy, single acute (3.28–3.53 Gy/min)	C57BL/6J[d]	Not specified	1–24 months	–
Hoving et al. 2010 [S7]	250 kV X-rays, neck	14 Gy, single acute[b]	ApoE$^{-/-}$ [c], male	Not specified	4–30 weeks	Aspirin, NO releasing aspirin
Yu et al. 2011 [S8]	^{56}Fe ions (~190 keV/µm), upper aortic tree	2–5 Gy, single acute[b]	ApoE$^{-/-}$, male	10 weeks	13–40 weeks	–
Hoving et al. 2011 [S9]	250 kV X-rays, neck	14 Gy, single acute[b]	ApoE$^{-/-}$ [c], female	15–16 weeks	4–28 weeks	Atorvastatin, clopidogrel
			CD40L$^{-/-}$/ApoE$^{-/-}$ [c], male	8–11 weeks	4–28 weeks	Atorvastatin, clopidogrel
Hoving et al. 2012 [S10]	250 kV X-rays, neck	8–14 Gy, single acute[b]	C57BL/6J, ApoE$^{-/-}$ [c], both sexes	10–16 weeks	1 day–4 weeks	–
Ko et al. 2018 [S11]	^{137}Cs γ-rays, area not specified	2 Gy, single acute[b]	Ldlr$^{-/-}$, both sexes	Not specified	4 weeks	HFD, PCA
Newman et al. 2018 [S12]	^{137}Cs γ-rays, total body	12 Gy, 2 Frs (6 Gy/Fr[b]) 5 h apart	ApoE$^{-/-}$ [c], ApoE$^{-/-}$/SMC-YFP[c], male	9 weeks	9–24 weeks	HFD, BMT, PCA
Yuan et al. 2021 [S13]	225 kV X-rays, neck	5–20 Gy, single acute[b]	ApoE$^{-/-}$, female	8 weeks	8 weeks	HFD, PCA, chloroquine
Yamamoto et	^{137}Cs γ-	6 Gy, single	ApoE$^{-/-}$ [c, d]	11 weeks	4 weeks	HFD

al. 2021 [S14]	rays, total body	acute[b]				
Ait-Aissa et al. 2022 [S15]	220 kV X-rays, whole brain	12 Gy, single acute[b]	C57BL/6J, endothelial-specific MCU$^{-/-}$, both sexes	12 weeks	1–10 days	MitoTEMPO
Ait-Aissa et al. 2023 [S16]	220 kV X-rays, head and neck	12 Gy, single acute (3.6 Gy/min)	C57BL/6J, both sexes	12–16 weeks	1–10 days	Pravastatin, atorvastatin
Ait-Aissa et al. 2024 [S17]	220 kV X-rays, head and neck	12 Gy, single acute (3.6 Gy/min)	C57BL/6J, both sexes	12–16 weeks	1 year	Pravastatin

ApoE, apolipoprotein E. BMT, bone marrow transplantation. CD40L, cluster of differentiation 40 ligand. Fr, fraction. HFD, high-fat diet. Ldlr, low-density lipoprotein receptor. MCU, mitochondrial Ca^{2+} uniporter. MitoTEMPO, mitochondrial reactive oxygen species scavenger. NO, nitric oxide. PCA, partial carotid artery ligation. SMC-YFP, smooth muscle cell lineage tracing Myh11-ER^{T2}Cre ROSA-STOP-eYFP.

[a] A literature search on PubMed (https://pubmed.ncbi.nlm.nih.gov/?term=carotid+radiation+mouse) was undertaken on 26 February 2025, yielding 139 papers. Of these, 18 papers deemed eligible based on the title/abstract search underwent detailed review of full texts, and the above-listed 14 papers were judged to be relevant.

[b] Dose rate not specified.

[c] On a C57BL/6J background.

[d] Sex not specified.

References

[S4] F.A. Stewart, S. Heeneman, J. Te Poele, J. Kruse, N.S. Russell, M. Gijbels, et al., Ionizing radiation accelerates the development of atherosclerotic lesions in ApoE$^{-/-}$ mice and predisposes to an inflammatory plaque phenotype prone to hemorrhage, Am. J. Pathol. 168 (2006) 649–658, https://doi.org/10.2353/ajpath.2006.050409

[S5] S. Hoving, S. Heeneman, M.J. Gijbels, J.A. te Poele, N.S. Russell, M.J. Daemen, et al., Single-dose and fractionated irradiation promote initiation and progression of atherosclerosis and induce an inflammatory plaque phenotype in ApoE$^{-/-}$ mice, Int. J. Radiat. Oncol. Biol. Phys. 71 (2008) 848–857, https://doi.org/10.1016/j.ijrobp.2008.02.031

[S6] M.T. Lawton, C.M. Arnold, Y.J. Kim, E.A. Bogarin, C.L. Stewart, A.A. Wulfstat, et al., Radiation arteriopathy in the transgenic arteriovenous fistula model, Neurosurgery 62 (2008) 1129–1138, https://doi.org/10.1227/01.neu.0000325875.82999.3c

[S7] S. Hoving, S. Heeneman, M.J. Gijbels, J.A. te Poele, M. Bolla, J.F. Pol, et al., NO-donating aspirin and aspirin partially inhibit age-related atherosclerosis but not radiation-induced atherosclerosis in ApoE null mice, PLoS One 5 (2010) e12874, https://doi.org/10.1371/journal.pone.0012874

[S8] T. Yu, B.W. Parks, S. Yu, R. Srivastava, K. Gupta, X. Wu, et al., Iron-ion radiation accelerates atherosclerosis in apolipoprotein E-deficient mice, Radiat. Res. 175 (2011) 766–773, https://doi.org/10.1667/RR2482.1

[S9] S. Hoving, S. Heeneman, M.J. Gijbels, J.A. te Poele, J.F. Pol, K. Gabriels, et al., Anti-inflammatory and anti-thrombotic intervention strategies using atorvastatin, clopidogrel and knock-down of CD40L do not modify radiation-induced atherosclerosis in ApoE null mice, Radiother. Oncol. 101 (2011) 100–108, https://doi.org/10.1016/j.radonc.2011.09.019

[S10] S. Hoving, S. Heeneman, M.J. Gijbels, J.A. te Poele, N. Visser, J. Cleutjens, et al., Irradiation induces different inflammatory and thrombotic responses in carotid arteries of wildtype C57BL/6J and atherosclerosis-prone ApoE$^{-/-}$ mice, Radiother. Oncol. 105 (2012) 365–370, https://doi.org/10.1016/j.radonc.2012.11.001

[S11] K.A. Ko, Y. Wang, S. Kotla, Y. Fujii, H.T. Vu, B.P. Venkatesulu, et al., Developing a reliable mouse model for cancer therapy-induced cardiovascular toxicity in cancer patients and survivors, Front. Cardiovasc. Med. 5 (2018) 26, https://doi.org/10.3389/fcvm.2018.00026

[S12] A.A. Newman, R.A. Baylis, D.L. Hess, S.D. Griffith, L.S. Shankman, O.A. Cherepanova, et al., Irradiation abolishes smooth muscle investment into vascular lesions in specific vascular beds, JCI Insight 3 (2018) e121017, https://doi.org/10.1172/jci.insight.121017

[S13] R. Yuan, Z. Sun, J. Cai, X. Yang, W. Zhang, C. Wu, et al., A novel anticancer therapeutic strategy to target autophagy accelerates radiation-associated atherosclerosis, Int. J. Radiat. Oncol. Biol. Phys. 109 (2021) 540–552, https://doi.org/10.1016/j.ijrobp.2020.09.007

[S14] Y. Yamamoto, M. Minami, K. Yoshida, M. Nagata, T. Miyata, T. Yang, et al., Irradiation accelerates plaque formation and cellular senescence in flow-altered carotid arteries of apolipoprotein E knock-out mice, J. Am. Heart Assoc. 10 (2021) e020712, https://doi.org/10.1161/JAHA.120.020712

[S15] K. Ait-Aissa, O.M. Koval, N.R. Lindsey, I.M. Grumbach, Mitochondrial Ca^{2+} uptake drives endothelial injury by radiation therapy, Arterioscler. Thromb. Vasc. Biol. 42 (2022) 1121–1136, https://doi.org/10.1161/ATVBAHA.122.317869

[S16] K. Ait-Aissa, L.N. Leng, N.R. Lindsey, X. Guo, D. Juhr, O.M. Koval, et al., Mechanisms by which statins protect endothelial cells from radiation-induced injury in the carotid artery, Front. Cardiovasc. Med. 10 (2023) 1133315, https://doi.org/10.3389/fcvm.2023.1133315

[S17] K. Ait-Aissa, X. Guo, M. Klemmensen, L.N. Leng, O.M. Koval, I.M. Grumbach, Short-term statin treatment reduces, and long-term statin treatment abolishes chronic vascular injury by radiation therapy, J. Am. Heart Assoc. 13 (2024) e033558, https://doi.org/10.1161/JAHA.123.033558

Table S2. List of Antibodies Used in Immunofluorescence Staining

Ab[a]	Dilutions	Reactions	Manufacturer	Catalog number
Primary Ab				
Rat anti CD31 MAb (Clone MEC 13.3)	1:200	O/N at 4˚C	BD Pharmingen	550274
Rabbit anti eNOS MAb (Clone D9A5L)	1:100	O/N at 4˚C	CST	32027
Rabbit anti cleaved caspase-3 MAb (Clone 5A1E)	1:100	O/N at 4˚C	CST	9664
Rabbit anti VE-cadherin PAb	1:200	O/N at 4˚C	abcam	ab33168
Rabbit anti TNF-α PAb	1:100	O/N at 4˚C	abcam	ab6671
Rabbit anti CD68 PAb	1:200	O/N at 4˚C	abcam	ab125212
Rabbit anti F4/80 MAb (Clone SP115)	1:100	O/N at 4˚C	abcam	ab111101
Rabbit anti CD3 PAb	1:100	O/N at 4˚C	abcam	ab5690
Rabbit anti cleaved N-terminal GSDMD MAb (Clone EPR20829-408)	1:100	O/N at 4˚C	abcam	ab215203
Rabbit anti TGF-β1 PAb	1:100	O/N at 4˚C	Sigma	SAB4502954-100UG
Mouse anti α-SMA MAb (Clone 1A4), Cy3-labelled	1:100	O/N at 4˚C	Sigma	C6198-2ML
Secondary Ab				
Alexa Fluor 488 conjugated donkey anti-rat IgG	1:500	4 h at rt	Invitrogen	A-21208
Alexa Fluor 594 conjugated donkey anti-	1:500	4 h at rt	Invitrogen	A-21207

rabbit IgG

Ab, antibody. α-SMA, α-smooth muscle actin. CD, cluster of differentiation. CST, Cell Signaling Technology. DAPI, 4',6-diamidino-2-phenylindole. eNOS, endothelial nitric oxide synthase. GSDMD, gasdermin D. IgG, immunoglobulin G. MAb, monoclonal antibody. O/N, overnight. PAb, polyclonal antibody. rt, room temperature. TGF-β1, transforming growth factor β1. TNF-α, tumor necrosis factor α. VE-cadherin, vascular endothelial cadherin.

[a] Sections reacted with a rat primary antibody were reacted with an Alexa Fluor 488 conjugated secondary antibody. Sections reacted with a rabbit primary antibody were reacted with an Alexa Fluor 594 conjugated secondary antibody. Cell nuclei were counterstained with DAPI (Cat. No. D523, Dojindo, Japan).

Table S3. Comparisons among Irradiation Regimens Based on All Endpoints Tested or Only on Significantly Changed Endpoints

Carotid

- At 6 months after starting irradiation
  - 6 irradiation regimens
    - 14 endpoints (Table S4)[a]
      - X-rays in 25 Frs > Acute X-rays ≥ γ-rays in 25 Frs > Acute γ-rays > X-rays in 100 Frs > Chronic γ-rays
    - 12 endpoints (Table S5)
      - X-rays in 25 Frs ≥ Acute X-rays ≥ γ-rays in 25 Frs > Acute γ-rays > X-rays in 100 Frs > Chronic γ-rays
  - 4 irradiation regimens
    - 14 endpoints (Table S6)[a]
      - X-rays in 25 Frs > Acute X-rays >> X-rays in 100 Frs > Chronic γ-rays
    - 13 endpoints (Table S7)
      - X-rays in 25 Frs > Acute X-rays >> X-rays in 100 Frs > Chronic γ-rays
- At 12 months after starting irradiation
  - 4 irradiation regimens
    - 14 endpoints (Table S8)[a]
      - Acute X-rays ≥ X-rays in 25 Frs > X-rays in 100 Frs > Chronic γ-rays
    - 9 endpoints (Table S9)
      - Acute X-rays = X-rays in 25 Frs >> X-rays in 100 Frs > Chronic γ-rays
- At 6 and 12 months after starting irradiation
  - 4 irradiation regimens
    - 28 endpoints (Table S10)[a]
      - X-rays in 25 Frs ≥ Acute X-rays >> X-rays in 100 Frs > Chronic γ-rays
    - 22 endpoints (Table S11)
      - X-rays in 25 Frs ≥ Acute X-rays >> X-rays in 100 Frs > Chronic γ-rays

Aorta

- At 6 months after starting irradiation
  - 6 irradiation regimens
    - 14 endpoints (Table S12)[a]
      - X-rays in 25 Frs > Acute X-rays > γ-rays in 25 Frs > Acute γ-rays > X-rays in 100 Frs >> Chronic γ-rays
    - 13 endpoints (Table S13)[b]

X-rays in 25 Frs > Acute X-rays > γ-rays in 25 Frs > Acute γ-rays > X-rays in 100 Frs >> Chronic γ-rays

4 irradiation regimens

14 endpoints (Table S14)[a]

X-rays in 25 Frs > Acute X-rays > X-rays in 100 Frs >> Chronic γ-rays

12 endpoints (Table S15)[b]

X-rays in 25 Frs > Acute X-rays > X-rays in 100 Frs >> Chronic γ-rays

At 12 months after starting irradiation

4 irradiation regimens

14 endpoints (Table S16)[a]

X-rays in 25 Frs > Acute X-rays >> X-rays in 100 Frs ≥ Chronic γ-rays

12 endpoints (Table S17)[b]

X-rays in 25 Frs ≥ Acute X-rays >> X-rays in 100 Frs ≥ Chronic γ-rays

At 6 and 12 months after starting irradiation

4 irradiation regimens

28 endpoints (Table S18)[a]

X-rays in 25 Frs > Acute X-rays >> X-rays in 100 Frs > Chronic γ-rays

24 endpoints (Table S19)[b]

X-rays in 25 Frs > Acute X-rays >> X-rays in 100 Frs > Chronic γ-rays

Carotid and aorta

At 6 months after starting irradiation

6 irradiation regimens

28 endpoints (Table S20)[a]

X-rays in 25 Frs > Acute X-rays > γ-rays in 25 Frs > Acute γ-rays > X-rays in 100 Frs >> Chronic γ-rays

25 endpoints (Table S21)[b]

X-rays in 25 Frs > Acute X-rays > γ-rays in 25 Frs > Acute γ-rays > X-rays in 100 Frs >> Chronic γ-rays

4 irradiation regimens

28 endpoints (Table S22)[a]

X-rays in 25 Frs > Acute X-rays >> X-rays in 100 Frs >> Chronic γ-rays

25 endpoints (Table S23)[b]

X-rays in 25 Frs > Acute X-rays >> X-rays in 100 Frs >> Chronic γ-rays

At 12 months after starting irradiation

4 irradiation regimens

28 endpoints (Table S24)[a]

X-rays in 25 Frs = Acute X-rays >> X-rays in 100 Frs > Chronic γ-rays

21 endpoints (Table S25)[b]

X-rays in 25 Frs = Acute X-rays >> X-rays in 100 Frs > Chronic γ-rays

At 6 and 12 months after starting irradiation

4 irradiation regimens

56 endpoints (Table S26)[a]

X-rays in 25 Frs > Acute X-rays >> X-rays in 100 Frs >> Chronic γ-rays

46 endpoints (Table S27)[b]

X-rays in 25 Frs > Acute X-rays >> X-rays in 100 Frs >> Chronic γ-rays

Frs, fractions. This table lists the results of a series of the Kolmogorov–Smirnov tests to compare effects of various irradiation regimens. See Table 1 for further summary, and Tables S4–S27 for more details.

[a] Comparisons were made for all endpoints tested (14 endpoints each for the carotid artery and the aorta).

[b] Comparisons were made only for endpoints that showed significant differences at least in one pair among 15 pairs for six irradiation regimens (Tables S5, S13 and S21) or among six pairs for four irradiation regimens (Tables S7, S9, S11, S15, S17, S19, S23, S25 and S27). Omitted endpoints are listed in the footnote a of each table.

Table S4. Comparisons among Six Irradiation Regimens Based on 14 Carotid Endpoints at 6 Months After Starting Irradiation

Irradiation regimens A vs B[a]	Alternative hypotheses[b]		Difference in each comparison[c]
	|A| > |B|	|A| < |B|	
Acute X-rays vs Acute γ-rays	2×10^{-3}	1×10^{0}	Acute X-rays > Acute γ-rays
γ-rays in 25 Frs vs Acute γ-rays	9×10^{-3}	1×10^{0}	γ-rays in 25 Frs > Acute γ-rays
X-rays in 25 Frs vs Acute γ-rays	2×10^{-4}	1×10^{0}	X-rays in 25 Frs > Acute γ-rays
Acute γ-rays vs X-rays in 100 Frs	2×10^{-2}	7×10^{-1}	Acute γ-rays > X-rays in 100 Frs
Acute γ-rays vs Chronic γ-rays	4×10^{-8}	1×10^{0}	Acute γ-rays >> Chronic γ-rays
Acute X-rays vs γ-rays in 25 Frs	1×10^{-1}	6×10^{-1}	Acute X-rays ≥ γ-rays in 25 Frs
X-rays in 25 Frs vs Acute X-rays	2×10^{-2}	1×10^{0}	X-rays in 25 Frs > Acute X-rays
Acute X-rays vs X-rays in 100 Frs	6×10^{-6}	1×10^{0}	Acute X-rays >> X-rays in 100 Frs
Acute X-rays vs Chronic γ-rays	1×10^{-10}	1×10^{0}	Acute X-rays >> Chronic γ-rays
X-rays in 25 Frs vs γ-rays in 25 Frs	6×10^{-3}	1×10^{0}	X-rays in 25 Frs > γ-rays in 25 Frs
γ-rays in 25 Frs vs X-rays in 100 Frs	6×10^{-3}	1×10^{0}	γ-rays in 25 Frs > X-rays in 100 Frs
γ-rays in 25 Frs vs Chronic γ-rays	2×10^{-8}	1×10^{0}	γ-rays in 25 Frs >> Chronic γ-rays
X-rays in 25 Frs vs X-rays in 100 Frs	2×10^{-6}	1×10^{0}	X-rays in 25 Frs >> X-rays in 100 Frs
X-rays in 25 Frs vs Chronic γ-rays	2×10^{-12}	1×10^{0}	X-rays in 25 Frs >> Chronic γ-rays
X-rays in 100 Frs vs Chronic γ-rays	2×10^{-4}	1×10^{0}	X-rays in 100 Frs > Chronic γ-rays
Overall difference[d]: X-rays in 25 Frs > Acute X-rays ≥ γ-rays in 25 Frs > Acute γ-rays > X-rays in 100 Frs > Chronic γ-rays			

Frs, fractions. See Figure S7 for the corresponding graphs, and Tables 1 and S3 for summary of a series of the Kolmogorov–Smirnov tests.

[a] Comparisons were made for all 14 carotid endpoints tested.

[b] vs null hypothesis that the absolute values of the radiation effects between the given comparison groups are equal for all endpoints (|A| = |B|). *P* value of the one-sided Kolmogorov–Smirnov goodness-of-fit test for the null hypothesis that the distribution of the *P* values from the individual tests is the standard uniform, which is true with no difference in radiation effects for all endpoints between the given comparison groups.

[c] Criteria for *P* values for |A| > |B| : >> ($P < 1 \times 10^{-5}$), > ($1 \times 10^{-5} \le P < 1 \times 10^{-1}$), ≥ ($1 \times 10^{-1} \le P < 3 \times 10^{-1}$), = ($P \ge 3 \times 10^{-1}$).

[d] Judged from differences in each of 15 comparisons shown immediately above.

Table S5. Comparisons among Six Irradiation Regimens Based on 12 Carotid Endpoints at 6 Months After Starting Irradiation

Irradiation regimens A vs B[a]	Alternative hypotheses[b]	Difference in each comparison[c]

	$\lvert A \rvert > \lvert B \rvert$	$\lvert A \rvert < \lvert B \rvert$	
Acute X-rays vs Acute γ-rays	4×10^{-2}	6×10^{-1}	Acute X-rays > Acute γ-rays
γ-rays in 25 Frs vs Acute γ-rays	4×10^{-2}	9×10^{-1}	γ-rays in 25 Frs > Acute γ-rays
X-rays in 25 Frs vs Acute γ-rays	8×10^{-3}	6×10^{-1}	X-rays in 25 Frs > Acute γ-rays
Acute γ-rays vs X-rays in 100 Frs	3×10^{-3}	9×10^{-1}	Acute γ-rays > X-rays in 100 Frs
Acute γ-rays vs Chronic γ-rays	4×10^{-8}	1×10^{0}	Acute γ-rays >> Chronic γ-rays
Acute X-rays vs γ-rays in 25 Frs	1×10^{-1}	3×10^{-1}	Acute X-rays ≥ γ-rays in 25 Frs
X-rays in 25 Frs vs Acute X-rays	1×10^{-1}	1×10^{0}	X-rays in 25 Frs ≥ Acute X-rays
Acute X-rays vs X-rays in 100 Frs	6×10^{-6}	1×10^{0}	Acute X-rays >> X-rays in 100 Frs
Acute X-rays vs Chronic γ-rays	1×10^{-10}	1×10^{0}	Acute X-rays >> Chronic γ-rays
X-rays in 25 Frs vs γ-rays in 25 Frs	1×10^{-1}	6×10^{-1}	X-rays in 25 Frs ≥ γ-rays in 25 Frs
γ-rays in 25 Frs vs X-rays in 100 Frs	3×10^{-3}	1×10^{0}	γ-rays in 25 Frs > X-rays in 100 Frs
γ-rays in 25 Frs vs Chronic γ-rays	2×10^{-8}	1×10^{0}	γ-rays in 25 Frs >> Chronic γ-rays
X-rays in 25 Frs vs X-rays in 100 Frs	2×10^{-6}	1×10^{0}	X-rays in 25 Frs >> X-rays in 100 Frs
X-rays in 25 Frs vs Chronic γ-rays	2×10^{-12}	1×10^{0}	X-rays in 25 Frs >> Chronic γ-rays
X-rays in 100 Frs vs Chronic γ-rays	2×10^{-4}	1×10^{0}	X-rays in 100 Frs > Chronic γ-rays
Overall difference[d]: X-rays in 25 Frs ≥ Acute X-rays ≥ γ-rays in 25 Frs > Acute γ-rays > X-rays in 100 Frs > Chronic γ-rays			

Frs, fractions. See Figure S7 for the corresponding graphs, and Tables 1 and S3 for summary of a series of the Kolmogorov–Smirnov tests.

[a] Comparisons were made for 12 carotid endpoints: 2 of 14 carotid endpoints (stained intensity per unit wall area and IMT-HC) were omitted due to lack of significant differences in any of the 15 pairs among six irradiation regimens.

[b] vs null hypothesis that the absolute values of the radiation effects between the given comparison groups are equal for all endpoints ($\lvert A \rvert = \lvert B \rvert$). *P* value of the one-sided Kolmogorov–Smirnov goodness-of-fit test for the null hypothesis that the distribution of the *P* values from the individual tests is the standard uniform, which is true with no difference in radiation effects for all endpoints between the given comparison groups.

[c] Criteria for *P* values for $\lvert A \rvert > \lvert B \rvert$: >> ($P < 1 \times 10^{-5}$), > ($1 \times 10^{-5} \leq P < 1 \times 10^{-1}$), ≥ ($1 \times 10^{-1} \leq P < 3 \times 10^{-1}$), = ($P \geq 3 \times 10^{-1}$).

[d] Judged from differences in each of 15 comparisons shown immediately above.

Table S6. Comparisons among Four Irradiation Regimens Based on 14 Carotid Endpoints at 6 Months After Starting Irradiation

Irradiation regimens A vs B[a]	Alternative hypotheses[b]		Difference in each comparison[c]
	$\lvert A \rvert > \lvert B \rvert$	$\lvert A \rvert < \lvert B \rvert$	
X-rays in 25 Frs vs Acute X-rays	2×10^{-2}	1×10^{0}	X-rays in 25 Frs > Acute X-rays
Acute X-rays vs X-rays in 100 Frs	6×10^{-6}	1×10^{0}	Acute X-rays >> X-rays in 100 Frs
Acute X-rays vs Chronic γ-rays	1×10^{-10}	1×10^{0}	Acute X-rays >> Chronic γ-rays
X-rays in 25 Frs vs X-rays in 100 Frs	2×10^{-6}	1×10^{0}	X-rays in 25 Frs >> X-rays in 100 Frs
X-rays in 25 Frs vs Chronic γ-rays	2×10^{-12}	1×10^{0}	X-rays in 25 Frs >> Chronic γ-rays
X-rays in 100 Frs vs Chronic γ-rays	2×10^{-4}	1×10^{0}	X-rays in 100 Frs > Chronic γ-rays
Overall difference[d]: X-rays in 25 Frs > Acute X-rays >> X-rays in 100 Frs > Chronic γ-rays			

Frs, fractions. See Figure S9 for the corresponding graphs, and Tables 1 and S3 for summary of a series of the Kolmogorov–Smirnov tests.

[a] Comparisons were made for all 14 carotid endpoints tested.

[b] vs null hypothesis that the absolute values of the radiation effects between the given comparison groups are equal for all endpoints (|A| = |B|). P value of the one-sided Kolmogorov–Smirnov goodness-of-fit test for the null hypothesis that the distribution of the P values from the individual tests is the standard uniform, which is true with no difference in radiation effects for all endpoints between the given comparison groups.

[c] Criteria for P values for |A| > |B| : >> ($P < 1 \times 10^{-5}$), > ($1 \times 10^{-5} \leq P < 1 \times 10^{-1}$), ≥ ($1 \times 10^{-1} \leq P < 3 \times 10^{-1}$), = ($P \geq 3 \times 10^{-1}$).

[d] Judged from differences in each of 6 comparisons shown immediately above.

Table S7. Comparisons among Four Irradiation Regimens Based on 13 Carotid Endpoints at 6 Months After Starting Irradiation

Irradiation regimens A vs B[a]	Alternative hypotheses[b]		Difference in each comparison[c]
	|A| > |B|	|A| < |B|	
X-rays in 25 Frs vs Acute X-rays	3×10^{-2}	1×10^{0}	X-rays in 25 Frs > Acute X-rays
Acute X-rays vs X-rays in 100 Frs	2×10^{-6}	1×10^{0}	Acute X-rays >> X-rays in 100 Frs
Acute X-rays vs Chronic γ-rays	2×10^{-11}	1×10^{0}	Acute X-rays >> Chronic γ-rays
X-rays in 25 Frs vs X-rays in 100 Frs	2×10^{-6}	1×10^{0}	X-rays in 25 Frs >> X-rays in 100 Frs
X-rays in 25 Frs vs Chronic γ-rays	9×10^{-12}	1×10^{0}	X-rays in 25 Frs >> Chronic γ-rays
X-rays in 100 Frs vs Chronic γ-rays	2×10^{-4}	1×10^{0}	X-rays in 100 Frs > Chronic γ-rays
Overall difference[d]: X-rays in 25 Frs > Acute X-rays >> X-rays in 100 Frs > Chronic γ-rays			

Frs, fractions. See Figure S9 for the corresponding graphs, and Tables 1 and S3 for summary of a series of the Kolmogorov–Smirnov tests.

[a] Comparisons were made for 13 carotid endpoints: 1 of 14 carotid endpoints (stained intensity per unit wall area) was omitted due to lack of significant differences in any of the 6 pairs among four irradiation regimens.

[b] vs null hypothesis that the absolute values of the radiation effects between the given comparison groups are equal for all endpoints (|A| = |B|). P value of the one-sided Kolmogorov–Smirnov goodness-of-fit test for the null hypothesis that the distribution of the P values from the individual tests is the standard uniform, which is true with no difference in radiation effects for all endpoints between the given comparison groups.

[c] Criteria for P values for |A| > |B| : >> ($P < 1 \times 10^{-5}$), > ($1 \times 10^{-5} \leq P < 1 \times 10^{-1}$), ≥ ($1 \times 10^{-1} \leq P < 3 \times 10^{-1}$), = ($P \geq 3 \times 10^{-1}$).

[d] Judged from differences in each of 6 comparisons shown immediately above.

Table S8. Comparisons among Four Irradiation Regimens Based on 14 Carotid Endpoints at 12 Months After Starting Irradiation

Irradiation regimens A vs B[a]	Alternative hypotheses[b]		Difference in each comparison[c]
	|A| > |B|	|A| < |B|	
X-rays in 25 Frs vs Acute X-rays	8×10^{-1}	2×10^{-1}	Acute X-rays ≥ X-rays in 25 Frs
Acute X-rays vs X-rays in 100 Frs	7×10^{-5}	1×10^{0}	Acute X-rays > X-rays in 100 Frs
Acute X-rays vs Chronic γ-rays	2×10^{-6}	1×10^{0}	Acute X-rays >> Chronic γ-rays
X-rays in 25 Frs vs X-rays in 100 Frs	2×10^{-4}	1×10^{0}	X-rays in 25 Frs > X-rays in 100 Frs
X-rays in 25 Frs vs Chronic γ-rays	5×10^{-6}	1×10^{0}	X-rays in 25 Frs >> Chronic γ-rays
X-rays in 100 Frs vs Chronic γ-rays	2×10^{-2}	1×10^{0}	X-rays in 100 Frs > Chronic γ-rays

Overall difference[d]: Acute X-rays ≥ X-rays in 25 Frs > X-rays in 100 Frs > Chronic γ-rays

Frs, fractions. See Figure S9 for the corresponding graphs, and Tables 1 and S3 for summary of a series of the Kolmogorov–Smirnov tests.

[a] Comparisons were made for all 14 carotid endpoints tested.

[b] vs null hypothesis that the absolute values of the radiation effects between the given comparison groups are equal for all endpoints (|A| = |B|). *P* value of the one-sided Kolmogorov–Smirnov goodness-of-fit test for the null hypothesis that the distribution of the *P* values from the individual tests is the standard uniform, which is true with no difference in radiation effects for all endpoints between the given comparison groups.

[c] Criteria for *P* values for |A| > |B| : >> ($P < 1 \times 10^{-5}$), > ($1 \times 10^{-5} \leq P < 1 \times 10^{-1}$), ≥ ($1 \times 10^{-1} \leq P < 3 \times 10^{-1}$), = ($P \geq 3 \times 10^{-1}$).

[d] Judged from differences in each of 6 comparisons shown immediately above.

Table S9. Comparisons among Four Irradiation Regimens Based on 9 Carotid Endpoints at 12 Months After Starting Irradiation

Irradiation regimens A vs B[a]	Alternative hypotheses[b]		Difference in each comparison[c]
	|A| > |B|	|A| < |B|	
X-rays in 25 Frs vs Acute X-rays	9×10^{-1}	4×10^{-1}	X-rays in 25 Frs = Acute X-rays
Acute X-rays vs X-rays in 100 Frs	6×10^{-6}	1×10^{0}	Acute X-rays >> X-rays in 100 Frs
Acute X-rays vs Chronic γ-rays	2×10^{-6}	1×10^{0}	Acute X-rays >> Chronic γ-rays
X-rays in 25 Frs vs X-rays in 100 Frs	2×10^{-6}	1×10^{0}	X-rays in 25 Frs >> X-rays in 100 Frs
X-rays in 25 Frs vs Chronic γ-rays	1×10^{-7}	1×10^{0}	X-rays in 25 Frs >> Chronic γ-rays
X-rays in 100 Frs vs Chronic γ-rays	3×10^{-2}	9×10^{-1}	X-rays in 100 Frs > Chronic γ-rays
Overall difference[d]: Acute X-rays = X-rays in 25 Frs >> X-rays in 100 Frs > Chronic γ-rays			

Frs, fractions. See Figure S9 for the corresponding graphs, and Tables 1 and S3 for summary of a series of the Kolmogorov–Smirnov tests.

[a] Comparisons were made for 9 carotid endpoints: 5 of 14 carotid endpoints (CD68, F4/80, CD3, IMT-IF, and stained intensity per unit wall area) were omitted due to lack of significant differences in any of the 6 pairs among four irradiation regimens.

[b] vs null hypothesis that the absolute values of the radiation effects between the given comparison groups are equal for all endpoints (|A| = |B|). *P* value of the one-sided Kolmogorov–Smirnov goodness-of-fit test for the null hypothesis that the distribution of the *P* values from the individual tests is the standard uniform, which is true with no difference in radiation effects for all endpoints between the given comparison groups.

[c] Criteria for *P* values for |A| > |B| : >> ($P < 1 \times 10^{-5}$), > ($1 \times 10^{-5} \leq P < 1 \times 10^{-1}$), ≥ ($1 \times 10^{-1} \leq P < 3 \times 10^{-1}$), = ($P \geq 3 \times 10^{-1}$).

[d] Judged from differences in each of 6 comparisons shown immediately above.

Table S10. Comparisons among Four Irradiation Regimens Based on 28 Carotid Endpoints at 6 and 12 Months After Starting Irradiation

Irradiation regimens A vs B[a]	Alternative hypotheses[b]		Difference in each comparison[c]
	|A| > |B|	|A| < |B|	
X-rays in 25 Frs vs Acute X-rays	3×10^{-1}	8×10^{-1}	X-rays in 25 Frs ≥ Acute X-rays
Acute X-rays vs X-rays in 100 Frs	5×10^{-10}	1×10^{0}	Acute X-rays >> X-rays in 100 Frs

Acute X-rays vs Chronic γ-rays	5×10^{-15}	1×10^{0}	Acute X-rays >> Chronic γ-rays
X-rays in 25 Frs vs X-rays in 100 Frs	4×10^{-8}	1×10^{0}	X-rays in 25 Frs >> X-rays in 100 Frs
X-rays in 25 Frs vs Chronic γ-rays	2×10^{-16}	1×10^{0}	X-rays in 25 Frs >> Chronic γ-rays
X-rays in 100 Frs vs Chronic γ-rays	2×10^{-4}	9×10^{-1}	X-rays in 100 Frs > Chronic γ-rays
Overall difference[d]: X-rays in 25 Frs ≥ Acute X-rays >> X-rays in 100 Frs > Chronic γ-rays			

Frs, fractions. See Figure S9 for the corresponding graphs, and Tables 1 and S3 for summary of a series of the Kolmogorov–Smirnov tests.

[a] Comparisons were made for all 14 carotid endpoints each tested at 6 and 12 months after starting irradiation.

[b] vs null hypothesis that the absolute values of the radiation effects between the given comparison groups are equal for all endpoints (|A| = |B|). *P* value of the one-sided Kolmogorov–Smirnov goodness-of-fit test for the null hypothesis that the distribution of the *P* values from the individual tests is the standard uniform, which is true with no difference in radiation effects for all endpoints between the given comparison groups.

[c] Criteria for *P* values for |A| > |B| : >> ($P < 1 \times 10^{-5}$), > ($1 \times 10^{-5} \leq P < 1 \times 10^{-1}$), ≥ ($1 \times 10^{-1} \leq P < 3 \times 10^{-1}$), = ($P \geq 3 \times 10^{-1}$).

[d] Judged from differences in each of 6 comparisons shown immediately above.

Table S11. Comparisons among Four Irradiation Regimens Based on 22 Carotid Endpoints at 6 and 12 Months After Starting Irradiation

Irradiation regimens A vs B[a]	Alternative hypotheses[b]		Difference in each comparison[c]
	|A| > |B|	|A| < |B|	
X-rays in 25 Frs vs Acute X-rays	1×10^{-1}	9×10^{-1}	X-rays in 25 Frs ≥ Acute X-rays
Acute X-rays vs X-rays in 100 Frs	5×10^{-11}	1×10^{0}	Acute X-rays >> X-rays in 100 Frs
Acute X-rays vs Chronic γ-rays	4×10^{-17}	1×10^{0}	Acute X-rays >> Chronic γ-rays
X-rays in 25 Frs vs X-rays in 100 Frs	5×10^{-11}	1×10^{0}	X-rays in 25 Frs >> X-rays in 100 Frs
X-rays in 25 Frs vs Chronic γ-rays	8×10^{-18}	1×10^{0}	X-rays in 25 Frs >> Chronic γ-rays
X-rays in 100 Frs vs Chronic γ-rays	2×10^{-4}	9×10^{-1}	X-rays in 100 Frs > Chronic γ-rays
Overall difference[d]: X-rays in 25 Frs ≥ Acute X-rays >> X-rays in 100 Frs > Chronic γ-rays			

Frs, fractions. See Figure S9 for the corresponding graphs, and Tables 1 and S3 for summary of a series of the Kolmogorov–Smirnov tests.

[a] Comparisons were made for 22 carotid endpoints tested at 6 and 12 months after starting irradiation: 1 of 14 carotid endpoints (stained intensity per unit wall area) at 6 months after starting irradiation and 5 of 14 aortic endpoints (CD68, F4/80, CD3, IMT-IF, and stained intensity per unit wall area) at 12 months after starting irradiation were omitted due to lack of significant differences in any of the 6 pairs among four irradiation regimens.

[b] vs null hypothesis that the absolute values of the radiation effects between the given comparison groups are equal for all endpoints (|A| = |B|). *P* value of the one-sided Kolmogorov–Smirnov goodness-of-fit test for the null hypothesis that the distribution of the *P* values from the individual tests is the standard uniform, which is true with no difference in radiation effects for all endpoints between the given comparison groups.

[c] Criteria for *P* values for |A| > |B| : >> ($P < 1 \times 10^{-5}$), > ($1 \times 10^{-5} \leq P < 1 \times 10^{-1}$), ≥ ($1 \times 10^{-1} \leq P < 3 \times 10^{-1}$), = ($P \geq 3 \times 10^{-1}$).

[d] Judged from differences in each of 6 comparisons shown immediately above.

Table S12. Comparisons among Six Irradiation Regimens Based on 14 Aortic Endpoints at 6 Months After Starting Irradiation

Irradiation regimens A vs B[a]	Alternative hypotheses[b]		Difference in each comparison[c]
	$\|A\| > \|B\|$	$\|A\| < \|B\|$	
Acute X-rays vs Acute γ-rays	2×10^{-3}	9×10^{-1}	Acute X-rays > Acute γ-rays
γ-rays in 25 Frs vs Acute γ-rays	2×10^{-2}	4×10^{-1}	γ-rays in 25 Frs > Acute γ-rays
X-rays in 25 Frs vs Acute γ-rays	1×10^{-5}	6×10^{-1}	X-rays in 25 Frs > Acute γ-rays
Acute γ-rays vs X-rays in 100 Frs	7×10^{-2}	6×10^{-1}	Acute γ-rays > X-rays in 100 Frs
Acute γ-rays vs Chronic γ-rays	2×10^{-4}	9×10^{-1}	Acute γ-rays > Chronic γ-rays
Acute X-rays vs γ-rays in 25 Frs	3×10^{-3}	7×10^{-1}	Acute X-rays > γ-rays in 25 Frs
X-rays in 25 Frs vs Acute X-rays	9×10^{-2}	8×10^{-1}	X-rays in 25 Frs > Acute X-rays
Acute X-rays vs X-rays in 100 Frs	8×10^{-3}	9×10^{-1}	Acute X-rays > X-rays in 100 Frs
Acute X-rays vs Chronic γ-rays	7×10^{-10}	1×10^{0}	Acute X-rays >> Chronic γ-rays
X-rays in 25 Frs vs γ-rays in 25 Frs	1×10^{-4}	2×10^{-1}	X-rays in 25 Frs > γ-rays in 25 Frs
γ-rays in 25 Frs vs X-rays in 100 Frs	3×10^{-5}	5×10^{-1}	γ-rays in 25 Frs > X-rays in 100 Frs
γ-rays in 25 Frs vs Chronic γ-rays	8×10^{-10}	1×10^{0}	γ-rays in 25 Frs >> Chronic γ-rays
X-rays in 25 Frs vs X-rays in 100 Frs	2×10^{-5}	1×10^{0}	X-rays in 25 Frs > X-rays in 100 Frs
X-rays in 25 Frs vs Chronic γ-rays	3×10^{-10}	1×10^{0}	X-rays in 25 Frs >> Chronic γ-rays
X-rays in 100 Frs vs Chronic γ-rays	1×10^{-6}	1×10^{0}	X-rays in 100 Frs > Chronic γ-rays
Overall difference[d]: X-rays in 25 Frs > Acute X-rays > γ-rays in 25 Frs > Acute γ-rays > X-rays in 100 Frs >> Chronic γ-rays			

Frs, fractions. See Figure S8 for the corresponding graphs, and Tables 1 and S3 for summary of a series of the Kolmogorov–Smirnov tests.

[a] Comparisons were made for all 14 aortic endpoints tested.

[b] vs null hypothesis that the absolute values of the radiation effects between the given comparison groups are equal for all endpoints ($|A| = |B|$). P value of the one-sided Kolmogorov–Smirnov goodness-of-fit test for the null hypothesis that the distribution of the P values from the individual tests is the standard uniform, which is true with no difference in radiation effects for all endpoints between the given comparison groups.

[c] Criteria for P values for $|A| > |B|$: >> ($P < 1 \times 10^{-5}$), > ($1 \times 10^{-5} \leq P < 1 \times 10^{-1}$), ≥ ($1 \times 10^{-1} \leq P < 3 \times 10^{-1}$), = ($P \geq 3 \times 10^{-1}$).

[d] Judged from differences in each of 15 comparisons shown immediately above.

Table S13. Comparisons among Six Irradiation Regimens Based on 13 Aortic Endpoints at 6 Months After Starting Irradiation

Irradiation regimens A vs B[a]	Alternative hypotheses[b]		Difference in each comparison[c]
	$\|A\| > \|B\|$	$\|A\| < \|B\|$	
Acute X-rays vs Acute γ-rays	9×10^{-4}	9×10^{-1}	Acute X-rays > Acute γ-rays
γ-rays in 25 Frs vs Acute γ-rays	1×10^{-2}	3×10^{-1}	γ-rays in 25 Frs > Acute γ-rays
X-rays in 25 Frs vs Acute γ-rays	3×10^{-6}	6×10^{-1}	X-rays in 25 Frs >> Acute γ-rays
Acute γ-rays vs X-rays in 100 Frs	4×10^{-2}	6×10^{-1}	Acute γ-rays > X-rays in 100 Frs
Acute γ-rays vs Chronic γ-rays	8×10^{-5}	9×10^{-1}	Acute γ-rays > Chronic γ-rays
Acute X-rays vs γ-rays in 25 Frs	1×10^{-3}	9×10^{-1}	Acute X-rays > γ-rays in 25 Frs
X-rays in 25 Frs vs Acute X-rays	8×10^{-2}	8×10^{-1}	X-rays in 25 Frs > Acute X-rays
Acute X-rays vs X-rays in 100 Frs	4×10^{-3}	9×10^{-1}	Acute X-rays > X-rays in 100 Frs

Acute X-rays vs Chronic γ-rays	1×10^{-10}	1×10^{0}	Acute X-rays >> Chronic γ-rays
X-rays in 25 Frs vs γ-rays in 25 Frs	4×10^{-5}	5×10^{-1}	X-rays in 25 Frs > γ-rays in 25 Frs
γ-rays in 25 Frs vs X-rays in 100 Frs	1×10^{-4}	5×10^{-1}	γ-rays in 25 Frs > X-rays in 100 Frs
γ-rays in 25 Frs vs Chronic γ-rays	5×10^{-9}	1×10^{0}	γ-rays in 25 Frs >> Chronic γ-rays
X-rays in 25 Frs vs X-rays in 100 Frs	9×10^{-6}	1×10^{0}	X-rays in 25 Frs >> X-rays in 100 Frs
X-rays in 25 Frs vs Chronic γ-rays	5×10^{-11}	1×10^{0}	X-rays in 25 Frs >> Chronic γ-rays
X-rays in 100 Frs vs Chronic γ-rays	4×10^{-7}	1×10^{0}	X-rays in 100 Frs > Chronic γ-rays
Overall difference[d]: X-rays in 25 Frs > Acute X-rays > γ-rays in 25 Frs > Acute γ-rays > X-rays in 100 Frs >> Chronic γ-rays			

Frs, fractions. See Figure S8 for the corresponding graphs, and Tables 1 and S3 for summary of a series of the Kolmogorov–Smirnov tests.

[a] Comparisons were made for 13 aortic endpoints: 1 of 14 aortic endpoints (CD3) was omitted due to lack of significant differences in any of the 15 pairs among six irradiation regimens.

[b] vs null hypothesis that the absolute values of the radiation effects between the given comparison groups are equal for all endpoints (|A| = |B|). *P* value of the one-sided Kolmogorov–Smirnov goodness-of-fit test for the null hypothesis that the distribution of the *P* values from the individual tests is the standard uniform, which is true with no difference in radiation effects for all endpoints between the given comparison groups.

[c] Criteria for *P* values for |A| > |B| : >> ($P < 1 \times 10^{-5}$), > ($1 \times 10^{-5} \leq P < 1 \times 10^{-1}$), ≥ ($1 \times 10^{-1} \leq P < 3 \times 10^{-1}$), = ($P \geq 3 \times 10^{-1}$).

[d] Judged from differences in each of 15 comparisons shown immediately above.

Table S14. Comparisons among Four Irradiation Regimens Based on 14 Aortic Endpoints at 6 Months After Starting Irradiation

Irradiation regimens A vs B[a]	Alternative hypotheses[b]		Difference in each comparison[c]
	|A| > |B|	|A| < |B|	
X-rays in 25 Frs vs Acute X-rays	2×10^{-2}	8×10^{-1}	X-rays in 25 Frs > Acute X-rays
Acute X-rays vs X-rays in 100 Frs	2×10^{-3}	1×10^{0}	Acute X-rays > X-rays in 100 Frs
Acute X-rays vs Chronic γ-rays	7×10^{-10}	1×10^{0}	Acute X-rays >> Chronic γ-rays
X-rays in 25 Frs vs X-rays in 100 Frs	2×10^{-5}	1×10^{0}	X-rays in 25 Frs > X-rays in 100 Frs
X-rays in 25 Frs vs Chronic γ-rays	3×10^{-10}	1×10^{0}	X-rays in 25 Frs >> Chronic γ-rays
X-rays in 100 Frs vs Chronic γ-rays	1×10^{-6}	1×10^{0}	X-rays in 100 Frs >> Chronic γ-rays
Overall difference[d]: X-rays in 25 Frs > Acute X-rays > X-rays in 100 Frs >> Chronic γ-rays			

Frs, fractions. See Figure S10 for the corresponding graphs, and Tables 1 and S3 for summary of a series of the Kolmogorov–Smirnov tests.

[a] Comparisons were made for all 14 aortic endpoints tested.

[b] vs null hypothesis that the absolute values of the radiation effects between the given comparison groups are equal for all endpoints (|A| = |B|). *P* value of the one-sided Kolmogorov–Smirnov goodness-of-fit test for the null hypothesis that the distribution of the *P* values from the individual tests is the standard uniform, which is true with no difference in radiation effects for all endpoints between the given comparison groups.

[c] Criteria for *P* values for |A| > |B| : >> ($P < 1 \times 10^{-5}$), > ($1 \times 10^{-5} \leq P < 1 \times 10^{-1}$), ≥ ($1 \times 10^{-1} \leq P < 3 \times 10^{-1}$), = ($P \geq 3 \times 10^{-1}$).

[d] Judged from differences in each of 6 comparisons shown immediately above.

Table S15. Comparisons among Four Irradiation Regimens Based on 12 Aortic

Endpoints at 6 Months After Starting Irradiation

Irradiation regimens A vs B[a]	Alternative hypotheses[b]		Difference in each comparison[c]
	$\|A\| > \|B\|$	$\|A\| < \|B\|$	
X-rays in 25 Frs vs Acute X-rays	1×10^{-2}	7×10^{-1}	X-rays in 25 Frs > Acute X-rays
Acute X-rays vs X-rays in 100 Frs	2×10^{-3}	1×10^{0}	Acute X-rays > X-rays in 100 Frs
Acute X-rays vs Chronic γ-rays	6×10^{-10}	1×10^{0}	Acute X-rays >> Chronic γ-rays
X-rays in 25 Frs vs X-rays in 100 Frs	3×10^{-6}	1×10^{0}	X-rays in 25 Frs >> X-rays in 100 Frs
X-rays in 25 Frs vs Chronic γ-rays	8×10^{-11}	1×10^{0}	X-rays in 25 Frs >> Chronic γ-rays
X-rays in 100 Frs vs Chronic γ-rays	2×10^{-6}	1×10^{0}	X-rays in 100 Frs >> Chronic γ-rays
Overall difference[d]: X-rays in 25 Frs > Acute X-rays > X-rays in 100 Frs >> Chronic γ-rays			

Frs, fractions. See Figure S10 for the corresponding graphs, and Tables 1 and S3 for summary of a series of the Kolmogorov–Smirnov tests.

[a] Comparisons were made for 12 aortic endpoints: 2 of 14 aortic endpoints (CD3 and IMT-IF) were omitted due to lack of significant differences in any of the 6 pairs among four irradiation regimens.

[b] vs null hypothesis that the absolute values of the radiation effects between the given comparison groups are equal for all endpoints ($|A| = |B|$). *P* value of the one-sided Kolmogorov–Smirnov goodness-of-fit test for the null hypothesis that the distribution of the *P* values from the individual tests is the standard uniform, which is true with no difference in radiation effects for all endpoints between the given comparison groups.

[c] Criteria for *P* values for $|A| > |B|$: >> ($P < 1 \times 10^{-5}$), > ($1 \times 10^{-5} \leq P < 1 \times 10^{-1}$), ≥ ($1 \times 10^{-1} \leq P < 3 \times 10^{-1}$), = ($P \geq 3 \times 10^{-1}$).

[d] Judged from differences in each of 6 comparisons shown immediately above.

Table S16. Comparisons among Four Irradiation Regimens based on 14 Aortic Endpoints at 12 Months After Starting Irradiation

Irradiation regimens A vs B[a]	Alternative hypotheses[b]		Difference in each comparison[c]
	$\|A\| > \|B\|$	$\|A\| < \|B\|$	
X-rays in 25 Frs vs Acute X-rays	7×10^{-2}	1×10^{0}	X-rays in 25 Frs > Acute X-rays
Acute X-rays vs X-rays in 100 Frs	3×10^{-6}	1×10^{0}	Acute X-rays >> X-rays in 100 Frs
Acute X-rays vs Chronic γ-rays	9×10^{-6}	1×10^{0}	Acute X-rays >> Chronic γ-rays
X-rays in 25 Frs vs X-rays in 100 Frs	7×10^{-9}	1×10^{0}	X-rays in 25 Frs >> X-rays in 100 Frs
X-rays in 25 Frs vs Chronic γ-rays	5×10^{-9}	1×10^{0}	X-rays in 25 Frs >> Chronic γ-rays
X-rays in 100 Frs vs Chronic γ-rays	2×10^{-1}	1×10^{0}	X-rays in 100 Frs ≥ Chronic γ-rays
Overall difference[d]: X-rays in 25 Frs > Acute X-rays >> X-rays in 100 Frs ≥ Chronic γ-rays			

Frs, fractions. See Figure S10 for the corresponding graphs, and Tables 1 and S3 for summary of a series of the Kolmogorov–Smirnov tests.

[a] Comparisons were made for all 14 aortic endpoints tested.

[b] vs null hypothesis that the absolute values of the radiation effects between the given comparison groups are equal for all endpoints ($|A| = |B|$). *P* value of the one-sided Kolmogorov–Smirnov goodness-of-fit test for the null hypothesis that the distribution of the *P* values from the individual tests is the standard uniform, which is true with no difference in radiation effects for all endpoints between the given comparison groups.

[c] Criteria for *P* values for $|A| > |B|$: >> ($P < 1 \times 10^{-5}$), > ($1 \times 10^{-5} \leq P < 1 \times 10^{-1}$), ≥ ($1 \times 10^{-1} \leq P < 3 \times 10^{-1}$), = ($P \geq 3 \times 10^{-1}$).

[d] Judged from differences in each of 6 comparisons shown immediately above.

Table S17. Comparisons among Four Irradiation Regimens Based on 12 Aortic Endpoints at 12 Months After Starting Irradiation

Irradiation regimens A vs B[a]	Alternative hypotheses[b]		Difference in each comparison[c]
	|A| > |B|	|A| < |B|	
X-rays in 25 Frs vs Acute X-rays	3×10^{-1}	9×10^{-1}	X-rays in 25 Frs ≥ Acute X-rays
Acute X-rays vs X-rays in 100 Frs	2×10^{-7}	1×10^{0}	Acute X-rays >> X-rays in 100 Frs
Acute X-rays vs Chronic γ-rays	9×10^{-7}	1×10^{0}	Acute X-rays >> Chronic γ-rays
X-rays in 25 Frs vs X-rays in 100 Frs	2×10^{-8}	1×10^{0}	X-rays in 25 Frs >> X-rays in 100 Frs
X-rays in 25 Frs vs Chronic γ-rays	4×10^{-9}	1×10^{0}	X-rays in 25 Frs >> Chronic γ-rays
X-rays in 100 Frs vs Chronic γ-rays	1×10^{-1}	9×10^{-1}	X-rays in 100 Frs ≥ Chronic γ-rays
Overall difference[d]: X-rays in 25 Frs ≥ Acute X-rays >> X-rays in 100 Frs ≥ Chronic γ-rays			

Frs, fractions. See Figure S10 for the corresponding graphs, and Tables 1 and S3 for summary of a series of the Kolmogorov–Smirnov tests.

[a] Comparisons were made for 12 aortic endpoints: 2 of 14 aortic endpoints (TNF-α and F4/80) were omitted due to lack of significant differences in any of the 6 pairs among four irradiation regimens.

[b] vs null hypothesis that the absolute values of the radiation effects between the given comparison groups are equal for all endpoints (|A| = |B|). *P* value of the one-sided Kolmogorov–Smirnov goodness-of-fit test for the null hypothesis that the distribution of the *P* values from the individual tests is the standard uniform, which is true with no difference in radiation effects for all endpoints between the given comparison groups.

[c] Criteria for *P* values for |A| > |B| : >> ($P < 1 \times 10^{-5}$), > ($1 \times 10^{-5} \le P < 1 \times 10^{-1}$), ≥ ($1 \times 10^{-1} \le P < 3 \times 10^{-1}$), = ($P \ge 3 \times 10^{-1}$).

[d] Judged from differences in each of 6 comparisons shown immediately above.

Table S18. Comparisons among Four Irradiation Regimens Based on 28 Aortic Endpoints at 6 and 12 Months After Starting Irradiation

Irradiation regimens A vs B[a]	Alternative hypotheses[b]		Difference in each comparison[c]
	|A| > |B|	|A| < |B|	
X-rays in 25 Frs vs Acute X-rays	6×10^{-3}	7×10^{-1}	X-rays in 25 Frs > Acute X-rays
Acute X-rays vs X-rays in 100 Frs	9×10^{-7}	9×10^{-1}	Acute X-rays >> X-rays in 100 Frs
Acute X-rays vs Chronic γ-rays	2×10^{-14}	1×10^{0}	Acute X-rays >> Chronic γ-rays
X-rays in 25 Frs vs X-rays in 100 Frs	2×10^{-12}	1×10^{0}	X-rays in 25 Frs >> X-rays in 100 Frs
X-rays in 25 Frs vs Chronic γ-rays	5×10^{-18}	1×10^{0}	X-rays in 25 Frs >> Chronic γ-rays
X-rays in 100 Frs vs Chronic γ-rays	4×10^{-5}	1×10^{0}	X-rays in 100 Frs > Chronic γ-rays
Overall difference[d]: X-rays in 25 Frs > Acute X-rays >> X-rays in 100 Frs > Chronic γ-rays			

Frs, fractions. See Figure S10 for the corresponding graphs, and Tables 1 and S3 for summary of a series of the Kolmogorov–Smirnov tests.

[a] Comparisons were made for all 14 aortic endpoints each tested at 6 and 12 months after starting irradiation.

[b] vs null hypothesis that the absolute values of the radiation effects between the given comparison groups are equal for all endpoints (|A| = |B|). *P* value of the one-sided Kolmogorov–Smirnov goodness-of-fit test for the null hypothesis that the distribution of the *P* values from the individual tests is the standard uniform, which is true with no difference

in radiation effects for all endpoints between the given comparison groups.

[c] Criteria for P values for |A| > |B| : >> ($P < 1 \times 10^{-5}$), > ($1 \times 10^{-5} \leq P < 1 \times 10^{-1}$), ≥ ($1 \times 10^{-1} \leq P < 3 \times 10^{-1}$), = ($P \geq 3 \times 10^{-1}$).

[d] Judged from differences in each of 6 comparisons shown immediately above.

Table S19. Comparisons among Four Irradiation Regimens Based on 24 Aortic Endpoints at 6 and 12 Months After Starting Irradiation

Irradiation regimens A vs B[a]	Alternative hypotheses[b]		Difference in each comparison[c]
	|A| > |B|	|A| < |B|	
X-rays in 25 Frs vs Acute X-rays	2×10^{-2}	6×10^{-1}	X-rays in 25 Frs > Acute X-rays
Acute X-rays vs X-rays in 100 Frs	3×10^{-8}	9×10^{-1}	Acute X-rays >> X-rays in 100 Frs
Acute X-rays vs Chronic γ-rays	1×10^{-15}	1×10^{0}	Acute X-rays >> Chronic γ-rays
X-rays in 25 Frs vs X-rays in 100 Frs	2×10^{-13}	1×10^{0}	X-rays in 25 Frs >> X-rays in 100 Frs
X-rays in 25 Frs vs Chronic γ-rays	6×10^{-18}	1×10^{0}	X-rays in 25 Frs >> Chronic γ-rays
X-rays in 100 Frs vs Chronic γ-rays	2×10^{-5}	1×10^{0}	X-rays in 100 Frs > Chronic γ-rays
Overall difference[d]: X-rays in 25 Frs > Acute X-rays >> X-rays in 100 Frs > Chronic γ-rays			

Frs, fractions. See Figure S10 for the corresponding graphs, and Tables 1 and S3 for summary of a series of the Kolmogorov–Smirnov tests.

[a] Comparisons were made for 24 aortic endpoints tested at 6 and 12 months after starting irradiation: 2 of 14 aortic endpoints (CD3 and IMT-IF) at 6 months after starting irradiation and 2 of 14 aortic endpoints (TNF-α and F4/80) at 12 months after starting irradiation were omitted due to lack of significant differences in any of the 6 pairs among four irradiation regimens.

[b] vs null hypothesis that the absolute values of the radiation effects between the given comparison groups are equal for all endpoints (|A| = |B|). P value of the one-sided Kolmogorov–Smirnov goodness-of-fit test for the null hypothesis that the distribution of the P values from the individual tests is the standard uniform, which is true with no difference in radiation effects for all endpoints between the given comparison groups.

[c] Criteria for P values for |A| > |B| : >> ($P < 1 \times 10^{-5}$), > ($1 \times 10^{-5} \leq P < 1 \times 10^{-1}$), ≥ ($1 \times 10^{-1} \leq P < 3 \times 10^{-1}$), = ($P \geq 3 \times 10^{-1}$).

[d] Judged from differences in each of 6 comparisons shown immediately above.

Table S20. Comparisons among Six Irradiation Regimens Based on 14 Carotid and 14 Aortic Endpoints at 6 Months After Starting Irradiation

Irradiation regimens A vs B[a]	Alternative hypotheses[b]		Difference in each comparison[c]
	|A| > |B|	|A| < |B|	
Acute X-rays vs Acute γ-rays	1×10^{-5}	1×10^{0}	Acute X-rays > Acute γ-rays
γ-rays in 25 Frs vs Acute γ-rays	4×10^{-4}	7×10^{-1}	γ-rays in 25 Frs > Acute γ-rays
X-rays in 25 Frs vs Acute γ-rays	2×10^{-7}	9×10^{-1}	X-rays in 25 Frs >> Acute γ-rays
Acute γ-rays vs X-rays in 100 Frs	2×10^{-3}	6×10^{-1}	Acute γ-rays > X-rays in 100 Frs
Acute γ-rays vs Chronic γ-rays	7×10^{-11}	1×10^{0}	Acute γ-rays >> Chronic γ-rays
Acute X-rays vs γ-rays in 25 Frs	3×10^{-3}	4×10^{-1}	Acute X-rays > γ-rays in 25 Frs
X-rays in 25 Frs vs Acute X-rays	8×10^{-3}	1×10^{0}	X-rays in 25 Frs > Acute X-rays
Acute X-rays vs X-rays in 100 Frs	2×10^{-7}	1×10^{0}	Acute X-rays >> X-rays in 100 Frs

Acute X-rays vs Chronic γ-rays	5×10^{-19}	1×10^{0}	Acute X-rays >> Chronic γ-rays
X-rays in 25 Frs vs γ-rays in 25 Frs	3×10^{-5}	6×10^{-1}	X-rays in 25 Frs > γ-rays in 25 Frs
γ-rays in 25 Frs vs X-rays in 100 Frs	6×10^{-7}	8×10^{-1}	γ-rays in 25 Frs >> X-rays in 100 Frs
γ-rays in 25 Frs vs Chronic γ-rays	2×10^{-17}	1×10^{0}	γ-rays in 25 Frs >> Chronic γ-rays
X-rays in 25 Frs vs X-rays in 100 Frs	5×10^{-9}	1×10^{0}	X-rays in 25 Frs >> X-rays in 100 Frs
X-rays in 25 Frs vs Chronic γ-rays	3×10^{-21}	1×10^{0}	X-rays in 25 Frs >> Chronic γ-rays
X-rays in 100 Frs vs Chronic γ-rays	3×10^{-9}	1×10^{0}	X-rays in 100 Frs >> Chronic γ-rays
Overall difference[d]: X-rays in 25 Frs > Acute X-rays > γ-rays in 25 Frs > Acute γ-rays > X-rays in 100 Frs >> Chronic γ-rays			

Frs, fractions. See Figures S7 and S8 for the corresponding graphs, and Tables 1 and S3 for summary of a series of the Kolmogorov–Smirnov tests.

[a] Comparisons were made for all 14 carotid and 14 aortic endpoints tested.

[b] vs null hypothesis that the absolute values of the radiation effects between the given comparison groups are equal for all endpoints (|A| = |B|). *P* value of the one-sided Kolmogorov–Smirnov goodness-of-fit test for the null hypothesis that the distribution of the *P* values from the individual tests is the standard uniform, which is true with no difference in radiation effects for all endpoints between the given comparison groups.

[c] Criteria for *P* values for |A| > |B| : >> ($P < 1 \times 10^{-5}$), > ($1 \times 10^{-5} \leq P < 1 \times 10^{-1}$), ≥ ($1 \times 10^{-1} \leq P < 3 \times 10^{-1}$), = ($P \geq 3 \times 10^{-1}$).

[d] Judged from differences in each of 15 comparisons shown immediately above.

Table S21. Comparisons among Six Irradiation Regimens Based on 12 Carotid and 13 Aortic Endpoints at 6 Months After Starting Irradiation

Irradiation regimens A vs B[a]	Alternative hypotheses[b]		Difference in each comparison[c]
	|A| > |B|	|A| < |B|	
Acute X-rays vs Acute γ-rays	1×10^{-6}	1×10^{0}	Acute X-rays >> Acute γ-rays
γ-rays in 25 Frs vs Acute γ-rays	9×10^{-5}	7×10^{-1}	γ-rays in 25 Frs > Acute γ-rays
X-rays in 25 Frs vs Acute γ-rays	1×10^{-7}	9×10^{-1}	X-rays in 25 Frs >> Acute γ-rays
Acute γ-rays vs X-rays in 100 Frs	8×10^{-4}	5×10^{-1}	Acute γ-rays > X-rays in 100 Frs
Acute γ-rays vs Chronic γ-rays	4×10^{-11}	1×10^{0}	Acute γ-rays >> Chronic γ-rays
Acute X-rays vs γ-rays in 25 Frs	3×10^{-3}	6×10^{-1}	Acute X-rays > γ-rays in 25 Frs
X-rays in 25 Frs vs Acute X-rays	8×10^{-3}	1×10^{0}	X-rays in 25 Frs > Acute X-rays
Acute X-rays vs X-rays in 100 Frs	1×10^{-7}	9×10^{-1}	Acute X-rays >> X-rays in 100 Frs
Acute X-rays vs Chronic γ-rays	7×10^{-20}	1×10^{0}	Acute X-rays >> Chronic γ-rays
X-rays in 25 Frs vs γ-rays in 25 Frs	3×10^{-5}	8×10^{-1}	X-rays in 25 Frs > γ-rays in 25 Frs
γ-rays in 25 Frs vs X-rays in 100 Frs	8×10^{-7}	8×10^{-1}	γ-rays in 25 Frs >> X-rays in 100 Frs
γ-rays in 25 Frs vs Chronic γ-rays	4×10^{-18}	1×10^{0}	γ-rays in 25 Frs >> Chronic γ-rays
X-rays in 25 Frs vs X-rays in 100 Frs	2×10^{-10}	1×10^{0}	X-rays in 25 Frs >> X-rays in 100 Frs
X-rays in 25 Frs vs Chronic γ-rays	2×10^{-20}	1×10^{0}	X-rays in 25 Frs >> Chronic γ-rays
X-rays in 100 Frs vs Chronic γ-rays	1×10^{-9}	1×10^{0}	X-rays in 100 Frs >> Chronic γ-rays
Overall difference[d]: X-rays in 25 Frs > Acute X-rays > γ-rays in 25 Frs > Acute γ-rays > X-rays in 100 Frs >> Chronic γ-rays			

Frs, fractions. See Figures S7 and S8 for the corresponding graphs, and Tables 1 and S3 for summary of a series of the Kolmogorov–Smirnov tests.

[a] Comparisons were made for 12 carotid and 13 aortic endpoints: 2 of 14 carotid endpoints (stained intensity per unit

wall area and IMT-HC) and 1 of 14 aortic endpoints (CD3) were omitted due to lack of significant differences in any of the 15 pairs among six irradiation regimens.

[b] vs null hypothesis that the absolute values of the radiation effects between the given comparison groups are equal for all endpoints (|A| = |B|). *P* value of the one-sided Kolmogorov–Smirnov goodness-of-fit test for the null hypothesis that the distribution of the *P* values from the individual tests is the standard uniform, which is true with no difference in radiation effects for all endpoints between the given comparison groups.

[c] Criteria for *P* values for |A| > |B| : >> ($P < 1 \times 10^{-5}$), > ($1 \times 10^{-5} \le P < 1 \times 10^{-1}$), ≥ ($1 \times 10^{-1} \le P < 3 \times 10^{-1}$), = ($P \ge 3 \times 10^{-1}$).

[d] Judged from differences in each of 15 comparisons shown immediately above.

Table S22. Comparisons among Four Irradiation Regimens Based on 14 Carotid and 14 Aortic Endpoints at 6 Months After Starting Irradiation

Irradiation regimens A vs B[a]	Alternative hypotheses[b]		Difference in each comparison[c]
	|A| > |B|	|A| < |B|	
X-rays in 25 Frs vs Acute X-rays	2×10^{-3}	1×10^{0}	X-rays in 25 Frs > Acute X-rays
Acute X-rays vs X-rays in 100 Frs	2×10^{-7}	1×10^{0}	Acute X-rays >> X-rays in 100 Frs
Acute X-rays vs Chronic γ-rays	5×10^{-19}	1×10^{0}	Acute X-rays >> Chronic γ-rays
X-rays in 25 Frs vs X-rays in 100 Frs	5×10^{-9}	1×10^{0}	X-rays in 25 Frs >> X-rays in 100 Frs
X-rays in 25 Frs vs Chronic γ-rays	3×10^{-21}	1×10^{0}	X-rays in 25 Frs >> Chronic γ-rays
X-rays in 100 Frs vs Chronic γ-rays	3×10^{-9}	1×10^{0}	X-rays in 100 Frs >> Chronic γ-rays
Overall difference[d]: X-rays in 25 Frs > Acute X-rays >> X-rays in 100 Frs >> Chronic γ-rays			

Frs, fractions. See Figures S9 and S10 for the corresponding graphs, and Tables 1 and S3 for summary of a series of the Kolmogorov–Smirnov tests.

[a] Comparisons were made for all 14 carotid and 14 aortic endpoints tested.

[b] vs null hypothesis that the absolute values of the radiation effects between the given comparison groups are equal for all endpoints (|A| = |B|). *P* value of the one-sided Kolmogorov–Smirnov goodness-of-fit test for the null hypothesis that the distribution of the *P* values from the individual tests is the standard uniform, which is true with no difference in radiation effects for all endpoints between the given comparison groups.

[c] Criteria for *P* values for |A| > |B| : >> ($P < 1 \times 10^{-5}$), > ($1 \times 10^{-5} \le P < 1 \times 10^{-1}$), ≥ ($1 \times 10^{-1} \le P < 3 \times 10^{-1}$), = ($P \ge 3 \times 10^{-1}$).

[d] Judged from differences in each of 6 comparisons shown immediately above.

Table S23. Comparisons among Four Irradiation Regimens Based on 13 Carotid and 12 Aortic Endpoints at 6 Months After Starting Irradiation

Irradiation regimens A vs B[a]	Alternative hypotheses[b]		Difference in each comparison[c]
	|A| > |B|	|A| < |B|	
X-rays in 25 Frs vs Acute X-rays	6×10^{-4}	1×10^{0}	X-rays in 25 Frs > Acute X-rays
Acute X-rays vs X-rays in 100 Frs	1×10^{-7}	1×10^{0}	Acute X-rays >> X-rays in 100 Frs
Acute X-rays vs Chronic γ-rays	7×10^{-20}	1×10^{0}	Acute X-rays >> Chronic γ-rays
X-rays in 25 Frs vs X-rays in 100 Frs	2×10^{-10}	1×10^{0}	X-rays in 25 Frs >> X-rays in 100 Frs
X-rays in 25 Frs vs Chronic γ-rays	1×10^{-21}	1×10^{0}	X-rays in 25 Frs >> Chronic γ-rays
X-rays in 100 Frs vs Chronic γ-rays	2×10^{-9}	1×10^{0}	X-rays in 100 Frs >> Chronic γ-rays

Overall difference[d]: X-rays in 25 Frs > Acute X-rays >> X-rays in 100 Frs >> Chronic γ-rays

Frs, fractions. See Figures S9 and S10 for the corresponding graphs, and Tables 1 and S3 for summary of a series of the Kolmogorov–Smirnov tests.

[a] Comparisons were made for 13 carotid and 12 aortic endpoints: 1 of 14 carotid endpoints (stained intensity per unit wall area) and 2 of 14 aortic endpoints (CD3 and IMT-IF) were omitted due to lack of significant differences in any of the 6 pairs among four irradiation regimens.

[b] vs null hypothesis that the absolute values of the radiation effects between the given comparison groups are equal for all endpoints (|A| = |B|). P value of the one-sided Kolmogorov–Smirnov goodness-of-fit test for the null hypothesis that the distribution of the P values from the individual tests is the standard uniform, which is true with no difference in radiation effects for all endpoints between the given comparison groups.

[c] Criteria for P values for |A| > |B| : >> ($P < 1 \times 10^{-5}$), > ($1 \times 10^{-5} \leq P < 1 \times 10^{-1}$), ≥ ($1 \times 10^{-1} \leq P < 3 \times 10^{-1}$), = ($P \geq 3 \times 10^{-1}$).

[d] Judged from differences in each of 6 comparisons shown immediately above.

Table S24. Comparisons among Four Irradiation Regimens Based on 14 Carotid and 14 Aortic Endpoints at 12 Months After Starting Irradiation

Irradiation regimens A vs B[a]	Alternative hypotheses[b]		Difference in each comparison[c]
	|A| > |B|	|A| < |B|	
X-rays in 25 Frs vs Acute X-rays	6×10^{-1}	7×10^{-1}	X-rays in 25 Frs = Acute X-rays
Acute X-rays vs X-rays in 100 Frs	5×10^{-10}	1×10^{0}	Acute X-rays >> X-rays in 100 Frs
Acute X-rays vs Chronic γ-rays	8×10^{-11}	1×10^{0}	Acute X-rays >> Chronic γ-rays
X-rays in 25 Frs vs X-rays in 100 Frs	2×10^{-11}	1×10^{0}	X-rays in 25 Frs >> X-rays in 100 Frs
X-rays in 25 Frs vs Chronic γ-rays	1×10^{-13}	1×10^{0}	X-rays in 25 Frs >> Chronic γ-rays
X-rays in 100 Frs vs Chronic γ-rays	1×10^{-2}	1×10^{0}	X-rays in 100 Frs > Chronic γ-rays
Overall difference[d]: X-rays in 25 Frs = Acute X-rays >> X-rays in 100 Frs > Chronic γ-rays			

Frs, fractions. See Figures S9 and S10 for the corresponding graphs, and Tables 1 and S3 for summary of a series of the Kolmogorov–Smirnov tests.

[a] Comparisons were made for all 14 carotid and 14 aortic endpoints tested.

[b] vs null hypothesis that the absolute values of the radiation effects between the given comparison groups are equal for all endpoints (|A| = |B|). P value of the one-sided Kolmogorov–Smirnov goodness-of-fit test for the null hypothesis that the distribution of the P values from the individual tests is the standard uniform, which is true with no difference in radiation effects for all endpoints between the given comparison groups.

[c] Criteria for P values for |A| > |B| : >> ($P < 1 \times 10^{-5}$), > ($1 \times 10^{-5} \leq P < 1 \times 10^{-1}$), ≥ ($1 \times 10^{-1} \leq P < 3 \times 10^{-1}$), = ($P \geq 3 \times 10^{-1}$).

[d] Judged from differences in each of 6 comparisons shown immediately above.

Table S25. Comparisons among Four Irradiation Regimens Based on 9 Carotid and 12 Aortic Endpoints at 12 Months After Starting Irradiation

Irradiation regimens A vs B[a]	Alternative hypotheses[b]		Difference in each comparison[c]
	|A| > |B|	|A| < |B|	
X-rays in 25 Frs vs Acute X-rays	7×10^{-1}	8×10^{-1}	X-rays in 25 Frs = Acute X-rays
Acute X-rays vs X-rays in 100 Frs	3×10^{-12}	1×10^{0}	Acute X-rays >> X-rays in 100 Frs

Acute X-rays vs Chronic γ-rays	5×10^{-12}	1×10^{0}	Acute X-rays >> Chronic γ-rays
X-rays in 25 Frs vs X-rays in 100 Frs	6×10^{-14}	1×10^{0}	X-rays in 25 Frs >> X-rays in 100 Frs
X-rays in 25 Frs vs Chronic γ-rays	2×10^{-15}	1×10^{0}	X-rays in 25 Frs >> Chronic γ-rays
X-rays in 100 Frs vs Chronic γ-rays	9×10^{-3}	1×10^{0}	X-rays in 100 Frs > Chronic γ-rays
Overall difference[d]: X-rays in 25 Frs = Acute X-rays >> X-rays in 100 Frs > Chronic γ-rays			

Frs, fractions. See Figures S9 and S10 for the corresponding graphs, and Tables 1 and S3 for summary of a series of the Kolmogorov–Smirnov tests.

[a] Comparisons were made for 9 carotid and 12 aortic endpoints: 5 of 14 carotid endpoints (CD68, F4/80, CD3, IMT-IF, and stained intensity per unit wall area) and 2 of 14 aortic endpoints (TNF-α and F4/80) were omitted due to lack of significant differences in any of the 6 pairs among four irradiation regimens.

[b] vs null hypothesis that the absolute values of the radiation effects between the given comparison groups are equal for all endpoints (|A| = |B|). *P* value of the one-sided Kolmogorov–Smirnov goodness-of-fit test for the null hypothesis that the distribution of the *P* values from the individual tests is the standard uniform, which is true with no difference in radiation effects for all endpoints between the given comparison groups.

[c] Criteria for *P* values for |A| > |B| : >> ($P < 1 \times 10^{-5}$), > ($1 \times 10^{-5} \leq P < 1 \times 10^{-1}$), ≥ ($1 \times 10^{-1} \leq P < 3 \times 10^{-1}$), = ($P \geq 3 \times 10^{-1}$).

[d] Judged from differences in each of 6 comparisons shown immediately above.

Table S26. Comparisons among Four Irradiation Regimens Based on 14 Carotid and 14 Aortic Endpoints at 6 Months After Starting Irradiation, and on 14 Carotid and 14 Aortic Endpoints at 12 Months After Starting Irradiation

Irradiation regimens A vs B[a]	Alternative hypotheses[b]		Difference in each comparison[c]
	|A| > |B|	|A| < |B|	
X-rays in 25 Frs vs Acute X-rays	8×10^{-2}	4×10^{-1}	X-rays in 25 Frs > Acute X-rays
Acute X-rays vs X-rays in 100 Frs	2×10^{-15}	1×10^{0}	Acute X-rays >> X-rays in 100 Frs
Acute X-rays vs Chronic γ-rays	4×10^{-28}	1×10^{0}	Acute X-rays >> Chronic γ-rays
X-rays in 25 Frs vs X-rays in 100 Frs	1×10^{-18}	1×10^{0}	X-rays in 25 Frs >> X-rays in 100 Frs
X-rays in 25 Frs vs Chronic γ-rays	2×10^{-33}	1×10^{0}	X-rays in 25 Frs >> Chronic γ-rays
X-rays in 100 Frs vs Chronic γ-rays	1×10^{-7}	1×10^{0}	X-rays in 100 Frs >> Chronic γ-rays
Overall difference[d]: X-rays in 25 Frs > Acute X-rays >> X-rays in 100 Frs >> Chronic γ-rays			

Frs, fractions. See Figures S9 and S10 for the corresponding graphs, and Tables 1 and S3 for summary of a series of the Kolmogorov–Smirnov tests.

[a] Comparisons were made for all 14 carotid and 14 aortic endpoints at 6 months after starting irradiation, and for all 14 carotid and 14 aortic endpoints at 12 months after starting irradiation.

[b] vs null hypothesis that the absolute values of the radiation effects between the given comparison groups are equal for all endpoints (|A| = |B|). *P* value of the one-sided Kolmogorov–Smirnov goodness-of-fit test for the null hypothesis that the distribution of the *P* values from the individual tests is the standard uniform, which is true with no difference in radiation effects for all endpoints between the given comparison groups.

[c] Criteria for *P* values for |A| > |B| : >> ($P < 1 \times 10^{-5}$), > ($1 \times 10^{-5} \leq P < 1 \times 10^{-1}$), ≥ ($1 \times 10^{-1} \leq P < 3 \times 10^{-1}$), = ($P \geq 3 \times 10^{-1}$).

[d] Judged from differences in each of 6 comparisons shown immediately above.

Table S27. Comparisons among Four Irradiation Regimens Based on 13 Carotid and

12 Aortic Endpoints at 6 Months After Starting Irradiation, and on 9 Carotid and 12 Aortic Endpoints at 12 Months After Starting Irradiation

Irradiation regimens A vs B[a]	Alternative hypotheses[b]		Difference in each comparison[c]
	$\lvert A\rvert > \lvert B\rvert$	$\lvert A\rvert < \lvert B\rvert$	
X-rays in 25 Frs vs Acute X-rays	2×10^{-2}	7×10^{-1}	X-rays in 25 Frs > Acute X-rays
Acute X-rays vs X-rays in 100 Frs	7×10^{-19}	1×10^{0}	Acute X-rays >> X-rays in 100 Frs
Acute X-rays vs Chronic γ-rays	1×10^{-30}	1×10^{0}	Acute X-rays >> Chronic γ-rays
X-rays in 25 Frs vs X-rays in 100 Frs	3×10^{-22}	1×10^{0}	X-rays in 25 Frs >> X-rays in 100 Frs
X-rays in 25 Frs vs Chronic γ-rays	8×10^{-35}	1×10^{0}	X-rays in 25 Frs >> Chronic γ-rays
X-rays in 100 Frs vs Chronic γ-rays	3×10^{-7}	1×10^{0}	X-rays in 100 Frs >> Chronic γ-rays
Overall difference[d]: X-rays in 25 Frs > Acute X-rays >> X-rays in 100 Frs >> Chronic γ-rays			

Frs, fractions. See Figures S9 and S10 for the corresponding graphs, and Tables 1 and S3 for summary of a series of the Kolmogorov–Smirnov tests.

[a] Comparisons were made for 13 carotid and 12 aortic endpoints at 6 months after starting irradiation as well as for 9 carotid and 12 aortic endpoints at 12 months after starting irradiation: 1 of 14 carotid endpoints (stained intensity per unit wall area) and 2 of 14 aortic endpoints (CD3 and IMT-IF) at 6 months after starting irradiation as well as 5 of 14 carotid endpoints (CD68, F4/80, CD3, IMT-IF, and stained intensity per unit wall area) and 2 of 14 aortic endpoints (TNF-α and F4/80) at 12 months after starting irradiation were omitted due to lack of significant differences in any of the 6 pairs among four irradiation regimens.

[b] vs null hypothesis that the absolute values of the radiation effects between the given comparison groups are equal for all endpoints ($\lvert A\rvert = \lvert B\rvert$). P value of the one-sided Kolmogorov–Smirnov goodness-of-fit test for the null hypothesis that the distribution of the P values from the individual tests is the standard uniform, which is true with no difference in radiation effects for all endpoints between the given comparison groups.

[c] Criteria for P values for $\lvert A\rvert > \lvert B\rvert$: >> ($P < 1 \times 10^{-5}$), > ($1 \times 10^{-5} \le P < 1 \times 10^{-1}$), ≥ ($1 \times 10^{-1} \le P < 3 \times 10^{-1}$), = ($P \ge 3 \times 10^{-1}$).

[d] Judged from differences in each of 6 comparisons shown immediately above.

Table S28. Comparisons among Carotid and Aortic Changes in Each or All of Irradiation Regimens

Irradiation regimens[a]	Alternative hypotheses[b]		Difference in each comparison[c]
	$\lvert A\rvert > \lvert B\rvert$	$\lvert A\rvert < \lvert B\rvert$	
At 6 months after starting irradiation			
Acute γ-rays	2×10^{-1}	1×10^{0}	Aorta ≥ Carotid
Acute X-rays	1×10^{-3}	6×10^{-1}	Aorta > Carotid
γ-rays in 25 Frs	2×10^{-1}	7×10^{-1}	Aorta ≥ Carotid
X-rays in 25 Frs	9×10^{-4}	6×10^{-1}	Aorta > Carotid
X-rays in 100 Frs	2×10^{-3}	4×10^{-1}	Aorta > Carotid
Chronic γ-rays	2×10^{-1}	4×10^{-2}	Carotid ≥ Aorta
Six irradiation regimens	1×10^{-4}	4×10^{-1}	Aorta > Carotid
At 12 months after starting irradiation			
Acute X-rays	5×10^{-1}	1×10^{-1}	Carotid > Aorta
X-rays in 25 Frs	5×10^{-3}	8×10^{-1}	Aorta > Carotid

X-rays in 100 Frs	1×10^{-2}	9×10^{-1}	Aorta > Carotid
Chronic γ-rays	2×10^{-1}	1×10^{0}	Aorta ≥ Carotid
Four irradiation regimens	1×10^{-1}	5×10^{-1}	Aorta ≥ Carotid

Frs, fractions. See Figures S11 and S12 for the corresponding graphs.

[a] Comparisons "A" vs "B" were made for 14 carotid and 14 aortic endpoints each at 6 or 12 months after starting irradiation, where "A" is the aorta and "B" is the carotid artery.

[b] vs null hypothesis that the absolute values of the radiation effects between the given comparison groups are equal for all endpoints (|A| = |B|). *P* value of the one-sided Kolmogorov–Smirnov goodness-of-fit test for the null hypothesis that the distribution of the *P* values from the individual tests is the standard uniform, which is true with no difference in radiation effects for all endpoints between the given comparison groups.

[c] Criteria for *P* values for |A| > |B| : >> ($P < 1 \times 10^{-5}$), > ($1 \times 10^{-5} \le P < 1 \times 10^{-1}$), ≥ ($1 \times 10^{-1} \le P < 3 \times 10^{-1}$), = ($P \ge 3 \times 10^{-1}$).

Table S29. Comparisons of Carotid and Aortic Endpoints among All Irradiation Regimens

Differences[a]	Endpoints	
	At 6 months after starting irradiation	At 12 months after starting irradiation
Aorta >> Carotid	IMT-HC	TGF-β1
Aorta > Carotid	VECs with subcellular fragments, VSMCs with subcellular fragments, TNF-α, TGF-β1, IMT-IF, Stained intensity/wall area	CD31 negativity, DAPI negativity
Aorta ≥ Carotid	–	Stained intensity/wall area
Aorta = Carotid	CD31 negativity, DAPI negativity, eNOS, VE-cadherin, F4/80	eNOS, VE-cadherin, VECs with subcellular fragments, VSMCs with subcellular fragments, TNF-α, CD68, F4/80, CD3, IMT-IF, IMT-HC
Carotid ≥ Aorta	–	–
Carotid > Aorta	CD3	–
Carotid >> Aorta	CD68	–

See Figure S11 for the corresponding graphs.

[a] Comparisons were made for six irradiation regimens at 6 months after starting irradiation and four irradiation regimens at 12 months after starting irradiation. *P* values were determined by t-test. Criteria for *P* values were: >> ($P < 1 \times 10^{-5}$), > ($1 \times 10^{-5} \le P < 1 \times 10^{-1}$), ≥ ($1 \times 10^{-1} \le P < 3 \times 10^{-1}$), = ($P \ge 3 \times 10^{-1}$).

Table S30. Approximation of the Data on Carotid or Aortic Changes as a Function of Age

Endpoints	Carotid	Aorta
CD31 negativity (%)	$y = 0.0414\exp(0.0715x)$. (r = 0.340)[a]	$y = 0.416\exp(0.0503x)$. (r = 0.377)[a]
DAPI negativity (%)	$y = 0.0414\exp(0.0715x)$. (r = 0.340)[a]	$y = 0.442\exp(0.0494x)$. (r = 0.376)[a]
eNOS (AU/μm²)	$y = 0.173x^2 - 38.4x + 3679$. $y = 3483\exp(-0.00882x)$. (r = –0.559)[a,b]	$y = -0.473x^2 + 19.4x + 5352$. (r = –0.497)[a]
VE-cadherin (dots)	$y = -0.00258x^2 - 0.0972x + 67.9$. (r = –0.175)	$y = -0.00783x^2 + 0.900x + 24.9$. (r = 0.167)[a]
VECs with subscellular fragments (%)	$y = -0.00390x^2 - 0.263x + 8.05$. (r = 0.309)	$y = 0.229\exp(-6.08x)$. (r = 0.302)

VSMCs with subscellular fragments (%)	$y = -0.00420x^2 -0.295x +7.07$. (r = 0.316)	$y = 0.133\exp(0.0543x)$. (r = 0.601)
TNF-α (AU/μm²)	$y = 92.9 + x^{0.951}$. (r = 0.312)	$y = 3.55x +325$. (r = 0.560)
CD68 (dots)	$y = 5.86\exp(0.0281x)$. (r = 0.302)	$y = 7.41 + x^{0.651}$. (r = 0.312)
F4/80 (dots)	$y = 0.152\exp(0.0346x)$. (r = 0.352)	$y = 0.000607x^2 -0.0547x +1.71$. (r = 0.0248)
CD3 (dots)	$y = 0.0483\exp(0.0472x)$. (r = 0.389)	$y = 15.5/(1 + \exp(-0.0217x +4.15))$. (r = 0.277)
TGF-β1 (AU/μm²)	$y = 67.6\exp(0.00672x)$. (r = 0.466)	$y = 0.0266x^2 -2.50x +326$. (r = 0.0675)
IMT-IF (μm)	$y = 18.3\exp(0.00635x)$. (r = 0.574)	$y = -0.000485x^2 +0.180x +17.1$. (r = 0.652)
Stained intensity/wall area (AU/μm²)	$y = 0.0142x^2 -0.890x +587$. (r = 0.209)	$y = -0.0136x^2 +2.85x +435$. (r = 0.550)
IMT-HC (μm)	$y = 0.129x +18.1$. (r = 0.463)	$y = 17.7\exp(0.00837x)$. (r = 0.463)

AIC, Akaike information criterion. AU, arbitrary unit. CD, cluster of differentiation. DAPI, 4',6-diamidino-2-phenylindole. eNOS, endothelial nitric oxide synthase. IMT-HC, intima-media thickness determined from histochemistry images. IMT-IF, intima-media thickness determined from immunofluorescence images. TGF-β1, transforming growth factor β1. TNF-α, tumor necrosis factor α. VEC, vascular endothelial cell. VE-cadherin, vascular endothelial cadherin. VSMC, vascular smooth muscle cell. The data in sham- or nonirradiated mice presented in Figures S13 and S14 were fitted to six models (linear, quadratic, exponential, power, logistic, and log). The best models were selected based on AIC and the analysis of deviance with a semiparametric bootstrap method (10,000 realizations) for the null hypothesis that the model indicated is correct, unless otherwise stated. *x*, age in weeks. *y*, each endpoint in above-indicated unit. r, Kendall rank correlation coefficient.

[a] The best models were selected based only on AIC, as the analysis of deviance suggested no models.

[b] Two models were selected as equally best.

Supplementary Figures

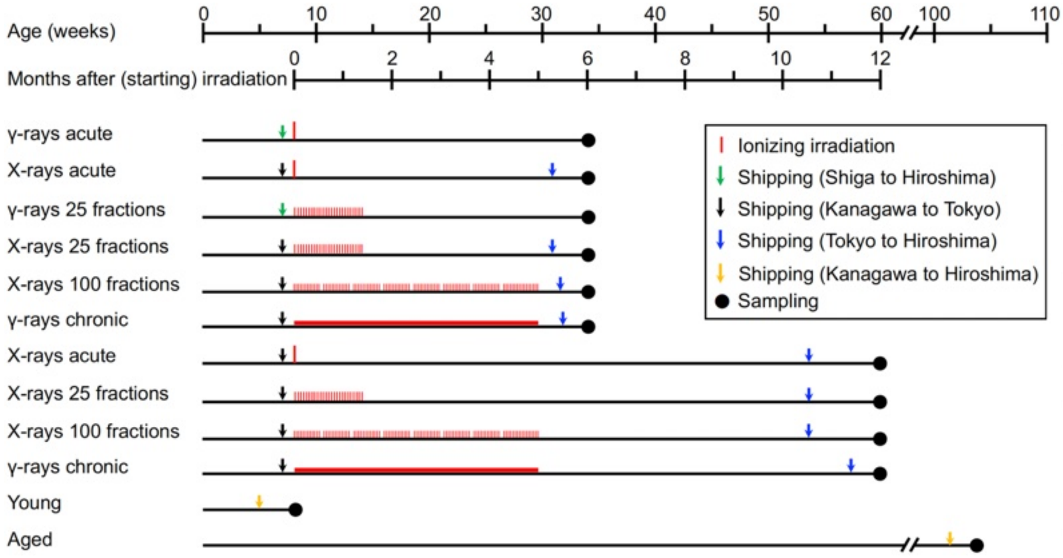

Figure S1. Detailed experimental timelines.

This study consists of 22 groups of male C57BL/6J mice. Before irradiation, mice shipped by car were acclimated. Mice aged 8 weeks were irradiated with 0 or 5 Gy of X-rays or γ-rays, either acutely (in 10 min), intermittently (in 25 fractions over 42 days, in 100 fractions over 153 days) or chronically (over 153 days). Prior to tissue sampling at 6 or 12 months after starting irradiation, mice shipped by car and air were acclimated. Tissues were also sampled from two nonirradiated control groups ("young" aged 8 weeks and "aged" aged 104 weeks). For more details, see Supplementary Methods.

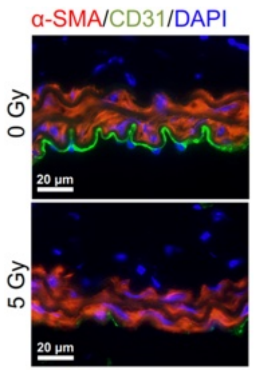

Figure S2. Representative immunofluorescence images for α-SMA in the carotid artery.

The carotid artery underwent dual immunofluorescence of CD31 (green) and α-SMA

(red), with cell nuclei counterstained with DAPI (blue). Images were taken at 6 months after starting irradiation with 0 Gy (an upper panel) or 5 Gy (a lower panel) of γ-rays in 25 fractions. See Figure 2A for the lower magnification images.

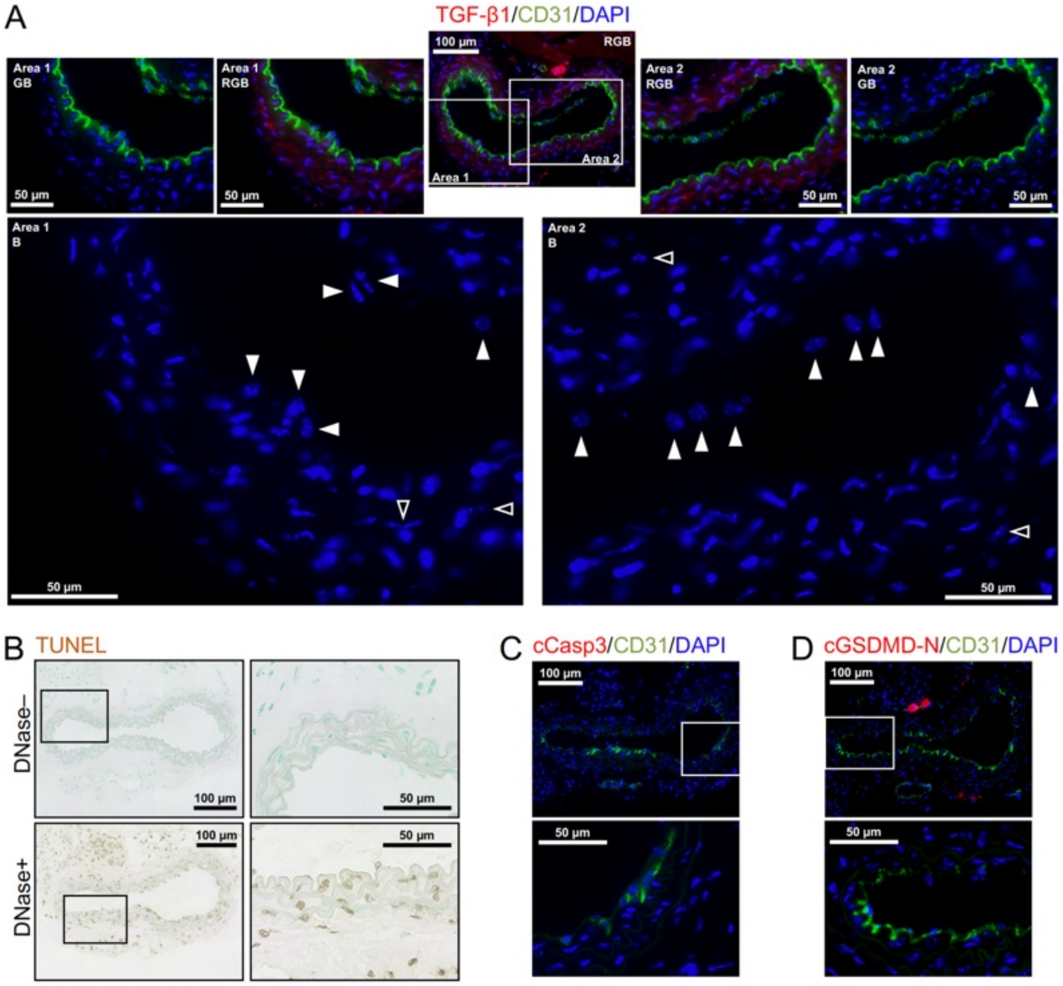

Figure S3. Representative images for carotid arterial cells with subcellular fragments.

The carotid artery taken at 6 months after starting irradiation with 5 Gy of γ-rays in 25 fractions was subjected to immunofluorescence or terminal deoxynucleotidyl transferase-mediated dUTP nick end labeling (TUNEL) staining. (**A**) Images for dual immunofluorescence of TGF-β1 (red, R) and CD31 (green, G), with cell nuclei counterstained with DAPI (blue, B). Each of the two boxed areas in the upper middle panel (the tiled image) is shown at higher magnification in three other panels, with the upper second right and left panels and the upper left- and rightmost panels further enlarged in the lower panels: the upper second left and right panels are for TGF-β1, CD31

and DAPI (RGB), the upper left- and rightmost panels for CD31 and DAPI (GB), and the lower panels for DAPI (B). Closed and open arrowheads in the lower panels point to vascular endothelial cells with subcellular fragments in the tunica intima and vascular smooth muscle cells with subcellular fragments in the tunica media, respectively. See Figure 3E and 3F for the quantitative analysis. (**B**) Images for TUNEL staining. As a positive control, sections shown in lower panels were treated with DNase I prior to the TUNEL staining. Boxed areas in the left panels (tiled images) are shown at higher magnification in the right panels. (**C**) Images for dual immunofluorescence of cleaved caspase-3 (cCasp3, as a marker for apoptosis) and CD31, with cell nuclei counterstained with DAPI. Boxed areas in the upper panel (the tiled image) is shown at higher magnification in the lower panel. (**D**) Images for dual immunofluorescence of cleaved N-terminal gasdermin D (cGSDMD-N, as a marker for pyroptosis) and CD31, with cell nuclei counterstained with DAPI. Boxed areas in the upper panel (the tiled image) is shown at higher magnification in the lower panel. Scale bars are as indicated.

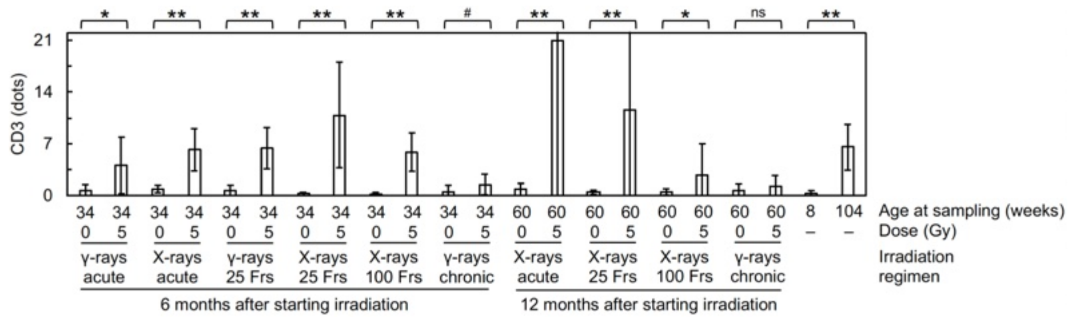

Figure S4. Quantitative analysis of immunofluorescence images for CD3 in the carotid artery.

Comparisons between the two groups (sham-irradiated vs irradiated, young vs aged) were made for CD3. *n* = 8–10 mice/group totaling 215 mice in 22 groups. Frs, fractions. *P* by the Welch's t-test or Wilcoxon rank sum test. **, *P* <0.001. *, 0.001≤ *P* <0.05. #, 0.05≤ *P* <0.1 (marginally significant), ns, *P* ≥0.1 (nonsignificant). See Figure 2G for representative images and Figure 3J for the wider y-axis range.

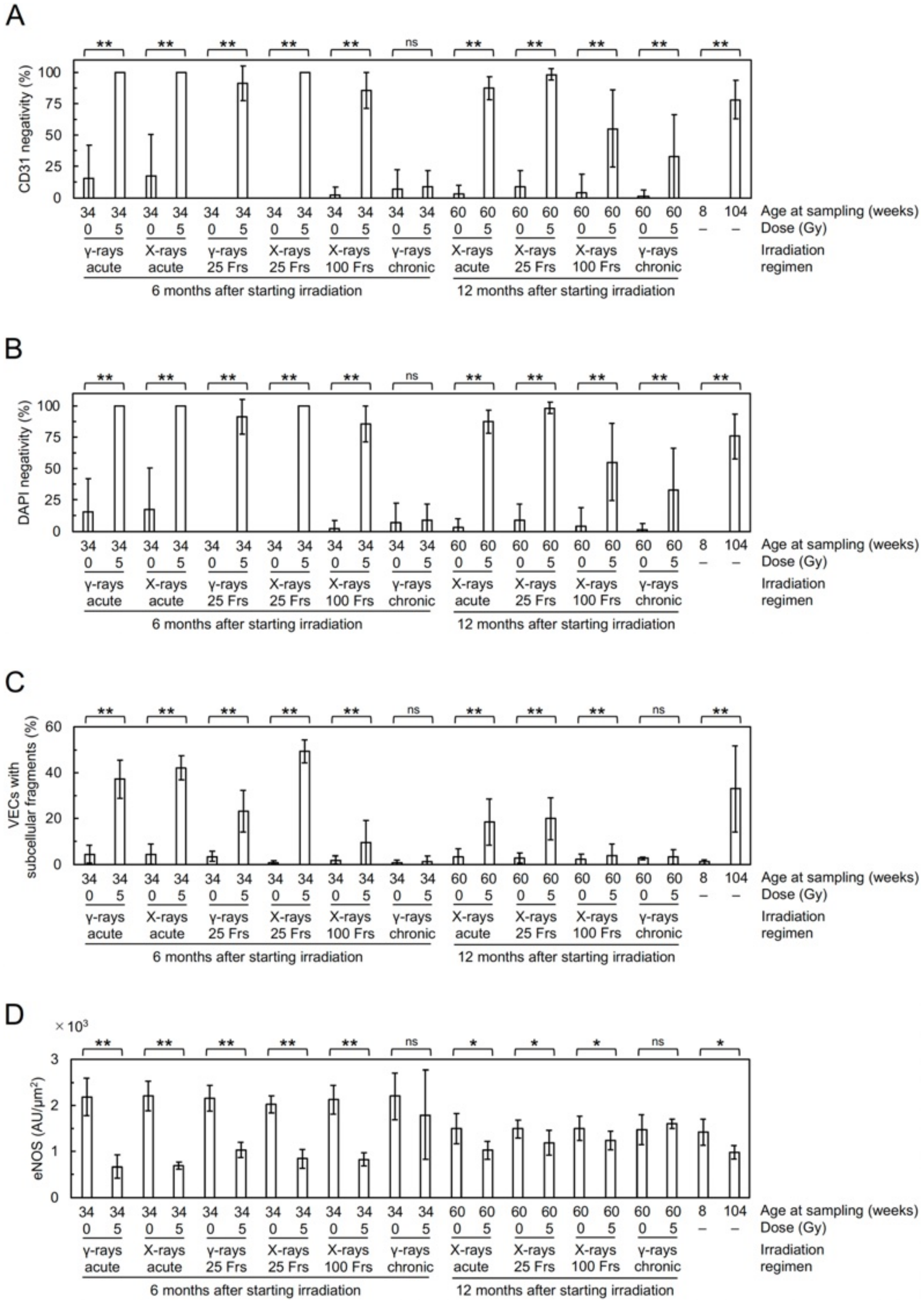
A
CD31 negativity (%)
B
DAPI negativity (%)
C
VECs with subcellular fragments (%)
D
× 10³
eNOS (AU/μm²)
** ns *
Age at sampling (weeks)
Dose (Gy)
Irradiation regimen
γ-rays acute
X-rays acute
γ-rays 25 Frs
X-rays 25 Frs
X-rays 100 Frs
γ-rays chronic
6 months after starting irradiation
12 months after starting irradiation

E

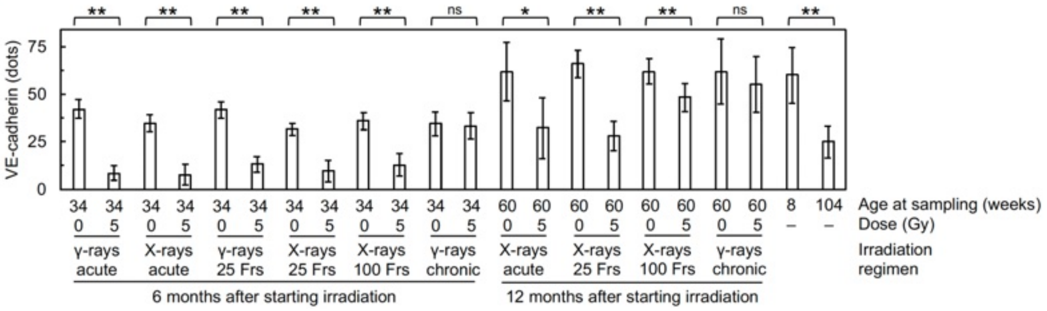
VE-cadherin (dots)
Age at sampling (weeks)
Dose (Gy)
Irradiation regimen
γ-rays acute
X-rays acute
γ-rays 25 Frs
X-rays 25 Frs
X-rays 100 Frs
γ-rays chronic
X-rays acute
X-rays 25 Frs
X-rays 100 Frs
γ-rays chronic
6 months after starting irradiation
12 months after starting irradiation

F

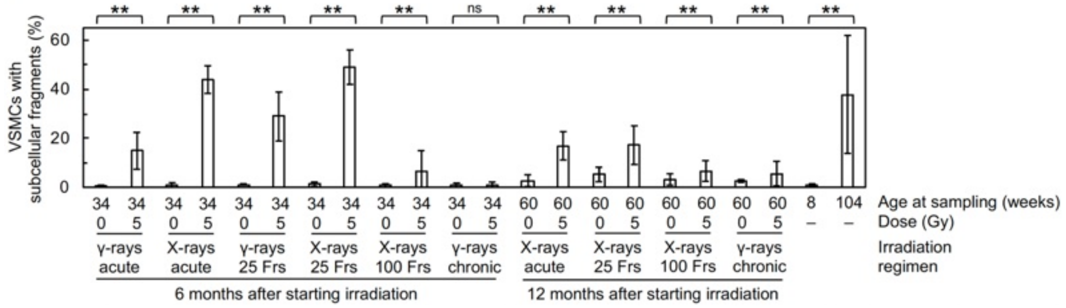
VSMCs with subcellular fragments (%)
Age at sampling (weeks)
Dose (Gy)
Irradiation regimen
γ-rays acute
X-rays acute
γ-rays 25 Frs
X-rays 25 Frs
X-rays 100 Frs
γ-rays chronic
X-rays acute
X-rays 25 Frs
X-rays 100 Frs
γ-rays chronic
6 months after starting irradiation
12 months after starting irradiation

G

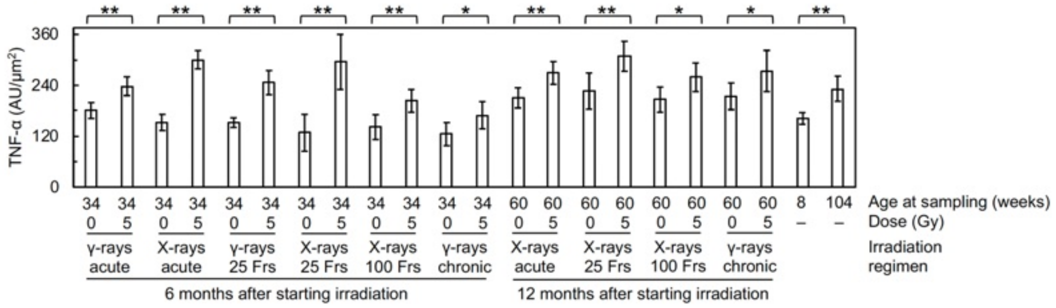
TNF-α (AU/μm²)
Age at sampling (weeks)
Dose (Gy)
Irradiation regimen
γ-rays acute
X-rays acute
γ-rays 25 Frs
X-rays 25 Frs
X-rays 100 Frs
γ-rays chronic
X-rays acute
X-rays 25 Frs
X-rays 100 Frs
γ-rays chronic
6 months after starting irradiation
12 months after starting irradiation

H

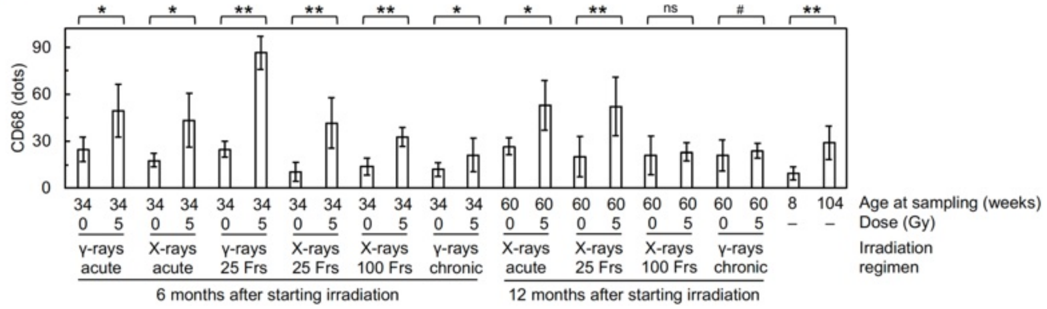
CD68 (dots)
Age at sampling (weeks)
Dose (Gy)
Irradiation regimen
γ-rays acute
X-rays acute
γ-rays 25 Frs
X-rays 25 Frs
X-rays 100 Frs
γ-rays chronic
X-rays acute
X-rays 25 Frs
X-rays 100 Frs
γ-rays chronic
6 months after starting irradiation
12 months after starting irradiation

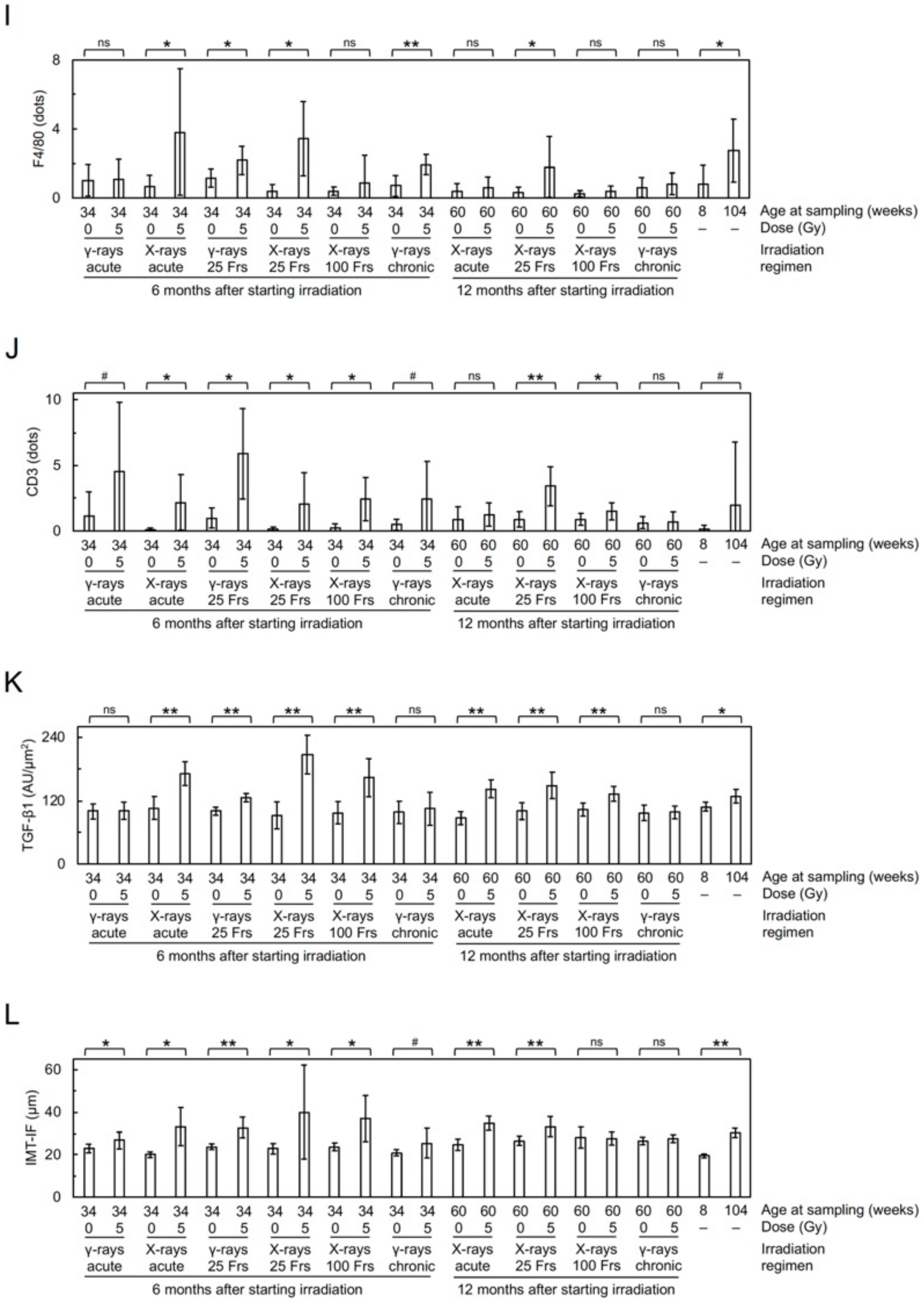
I
F4/80 (dots)
J
CD3 (dots)
K
TGF-β1 (AU/μm²)
L
IMT-IF (μm)
ns
*
**
#
Age at sampling (weeks)
Dose (Gy)
Irradiation regimen
γ-rays acute
X-rays acute
γ-rays 25 Frs
X-rays 25 Frs
X-rays 100 Frs
γ-rays chronic
6 months after starting irradiation
12 months after starting irradiation

M

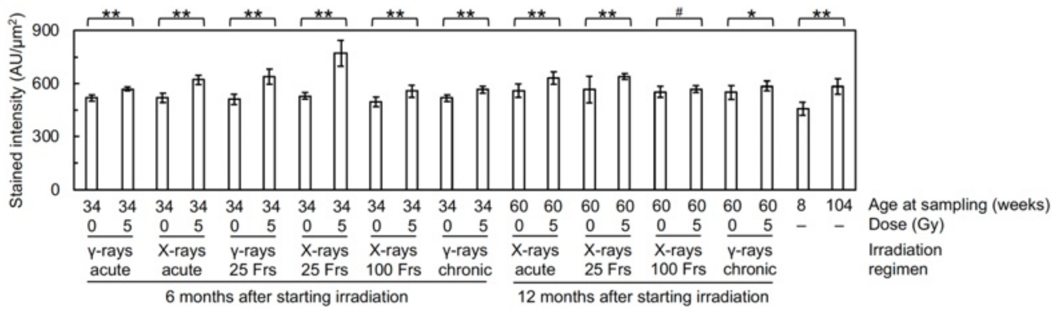

N

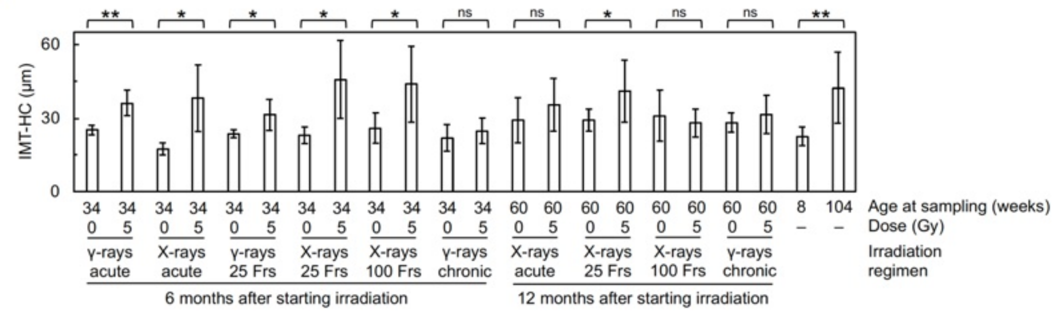

Figure S5. Quantitative analysis of immunofluorescence and histochemistry images in the aorta.

Comparisons between the two groups (sham-irradiated vs irradiated in each irradiation regimen, young vs aged) were made for (**A**) CD31 negativity, (**B**) DAPI negativity, (**C**) VECs with subcellular fragments, (**D**) eNOS, (**E**) VE-cadherin, (**F**) VSMCs with subcellular fragments, (**G**) TNF-α, (**H**) CD68, (**I**) F4/80, (**J**) CD3, (**K**) TGF-β1, (**L**) IMT-IF, (**M**) intensity of aniline blue per unit aortic wall area, and (**N**) IMT-HC. n = 7–10 mice/group totaling 210–214 mice in 22 groups. Frs, fractions. P by the Welch's t-test, Wilcoxon rank sum test, or Wald test. **, p <0.001. *, 0.001≤ P <0.05. #, 0.05≤ P <0.1 (marginally significant), ns, P ≥0.1 (nonsignificant). See Figures 3 and 4 for corresponding data in the carotid artery.

A

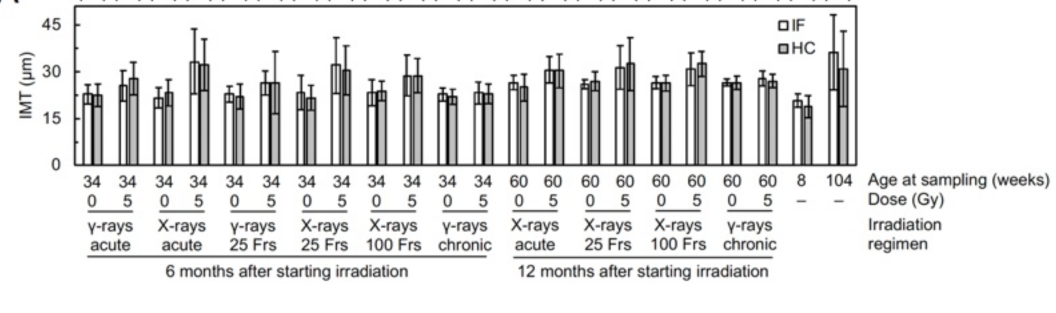

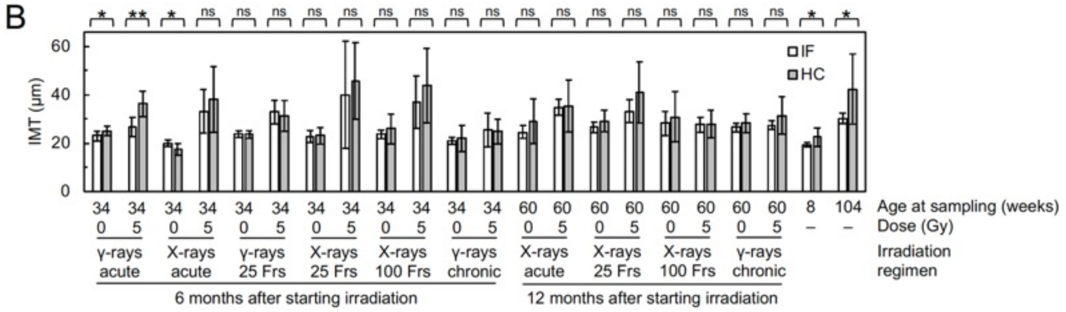

Figure S6. Comparisons between IMT-IF and IMT-HC.

Comparisons between IMT-IF and IMT-HC were made for the carotid artery and the aorta. (**A**) The carotid artery. r for comparisons among individual mice = 0.925. r for comparisons among groups of mice = 0.820. (**B**) The aorta. r for comparisons among individual mice = 0.554. r for comparisons among groups of mice = 0.905. *n* = 7–10 mice/group totaling 210–215 mice in 22 groups. Frs, fractions. *P* by the Welch's t-test, or Wilcoxon rank sum test. **, *P* <0.001. *, 0.001≤ *P* <0.05. #, 0.05≤ *P* <0.1 (marginally significant), ns, *P* ≥0.1 (nonsignificant). r, Pearson correlation coefficient. Data presented in Figures 3L, 4D, S5L and S5N were replotted for further comparisons.

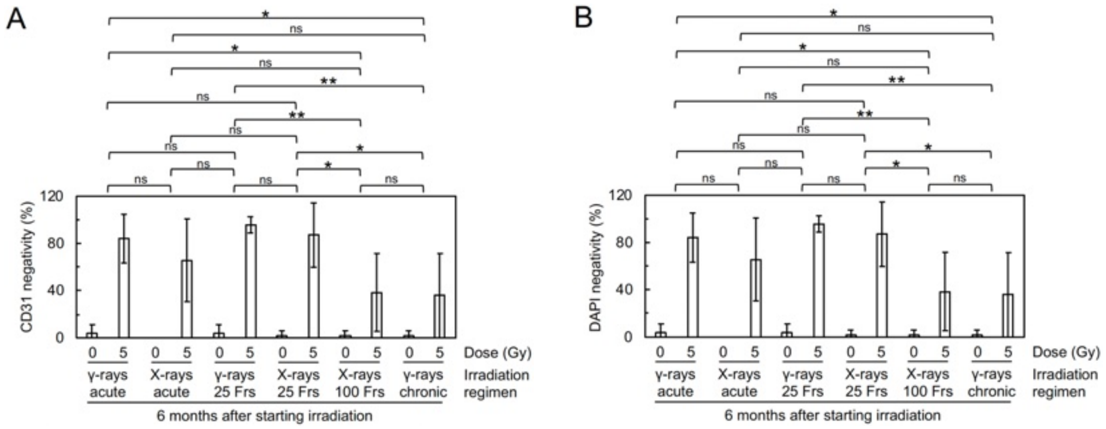

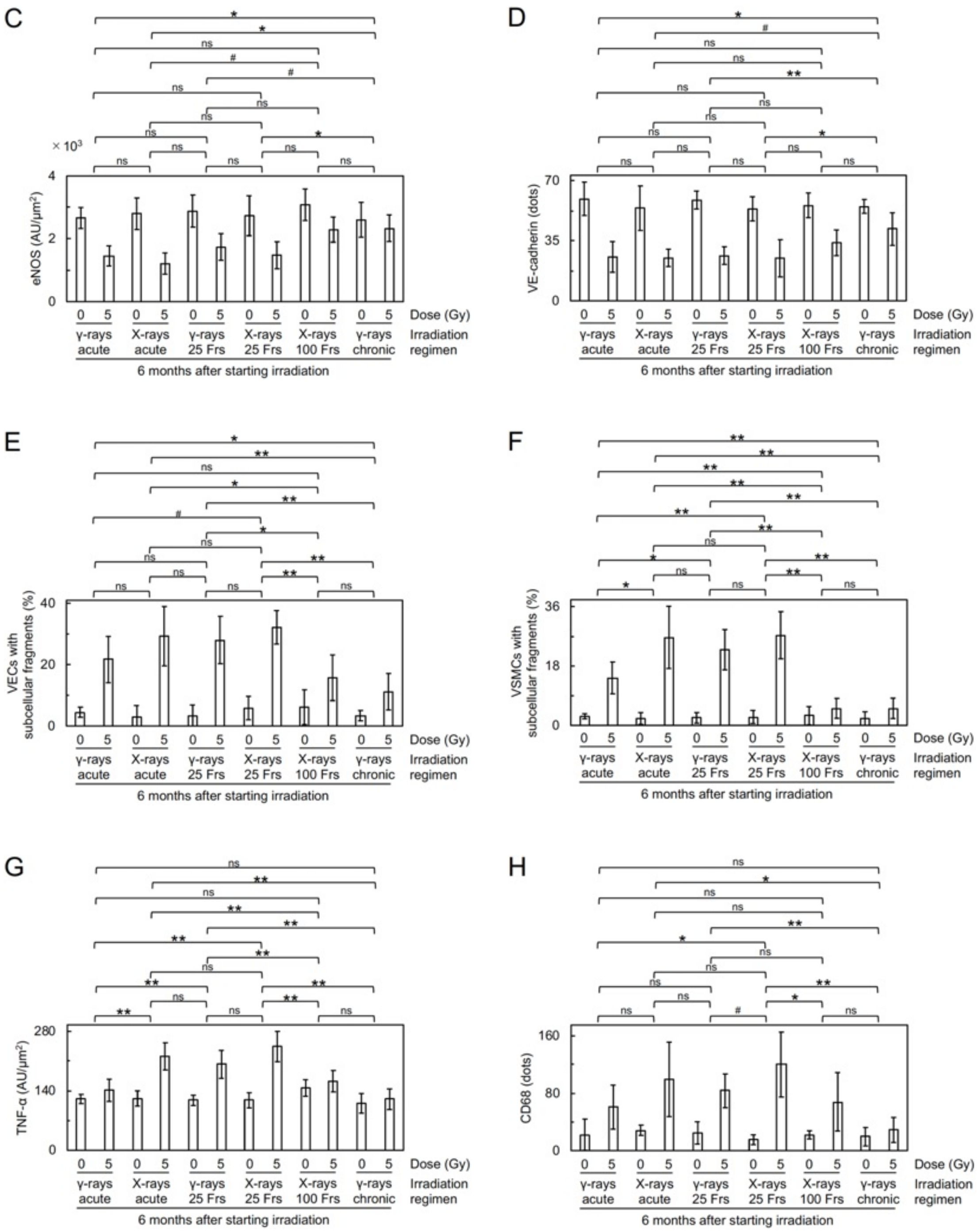
C
D
E
F
G
H
eNOS (AU/μm²)
× 10³
VE-cadherin (dots)
VECs with subcellular fragments (%)
VSMCs with subcellular fragments (%)
TNF-α (AU/μm²)
CD68 (dots)
Dose (Gy)
Irradiation regimen
γ-rays acute
X-rays acute
γ-rays 25 Frs
X-rays 25 Frs
X-rays 100 Frs
γ-rays chronic
6 months after starting irradiation

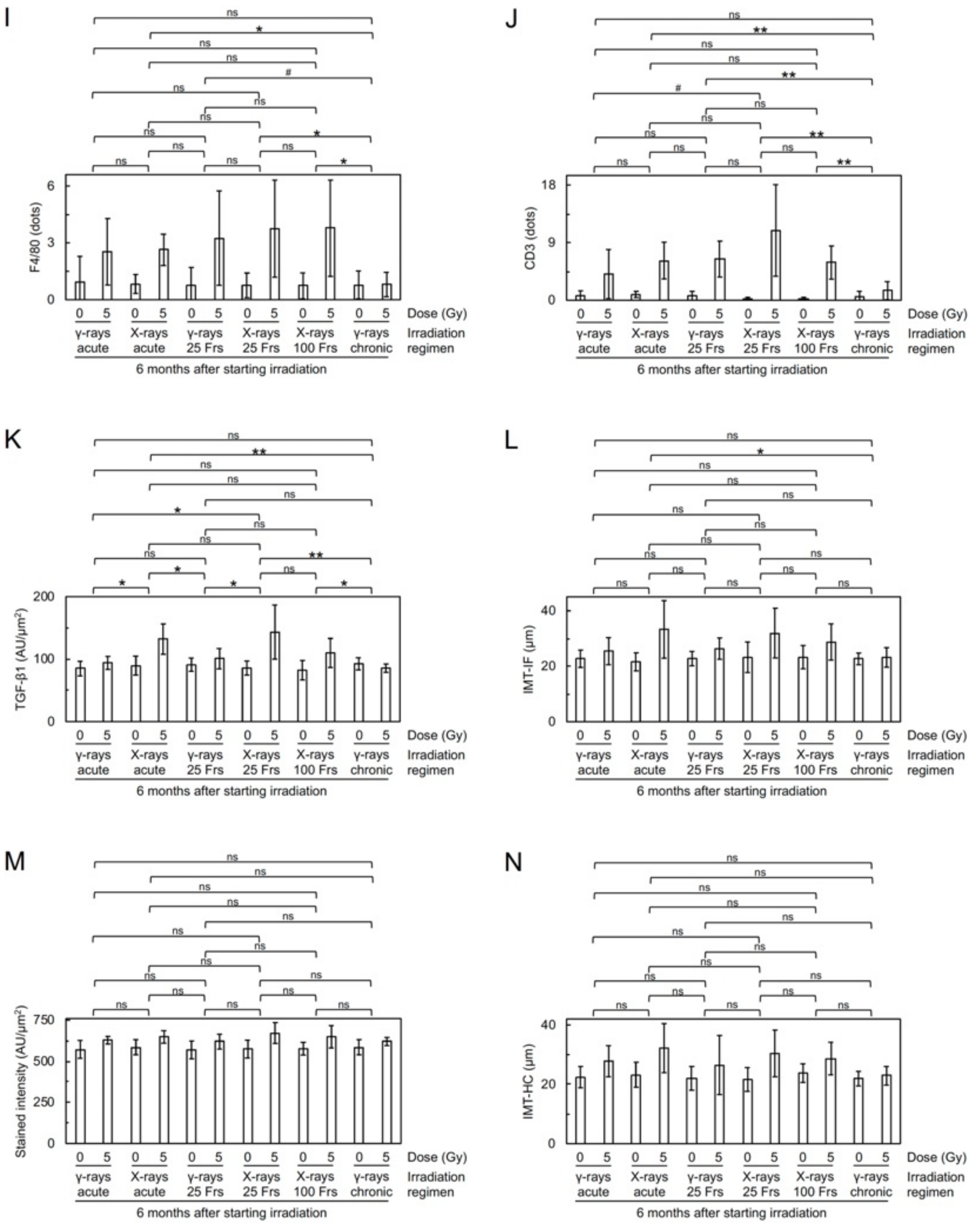

Figure S7. Comparisons of carotid endpoints among six irradiation regimens at 6 months after starting irradiation.

Comparisons between the two irradiation regimens (15 pairs for six irradiation regimens) were made for (**A**) CD31 negativity, (**B**) DAPI negativity, (**C**) eNOS, (**D**) VE-cadherin, (**E**) VECs with subcellular fragments, (**F**) VSMCs with subcellular fragments, (**G**) TNF-α, (**H**) CD68, (**I**) F4/80, (**J**) CD3, (**K**) TGF-β1, (**L**) IMT-IF, (**M**) intensity of aniline blue

per unit carotid wall area, and (**N**) IMT-HC. n = 9–10 mice/group totaling 118 mice in 12 groups. Frs, fractions. P by the analysis of deviance. **, P <0.001. *, 0.001≤ P <0.05. #, 0.05≤ P <0.1 (marginally significant), ns, P ≥0.1 (nonsignificant), after Bonferroni corrections. Data presented in Figures 3 and 4 were replotted for further comparisons. See Tables S3–S5, S20 and S21 for a series of the Kolmogorov–Smirnov tests to compare effects of various irradiation regimens.

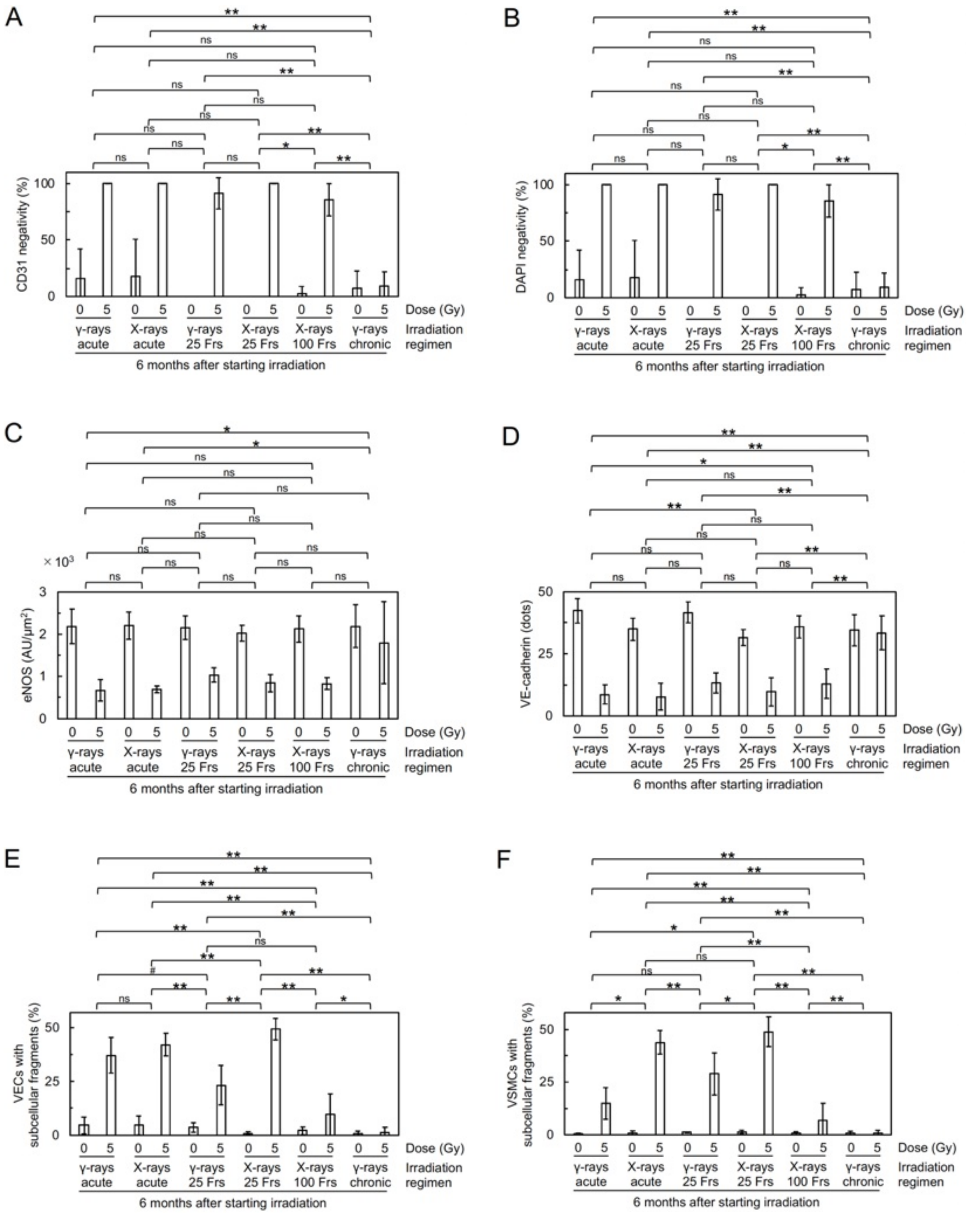

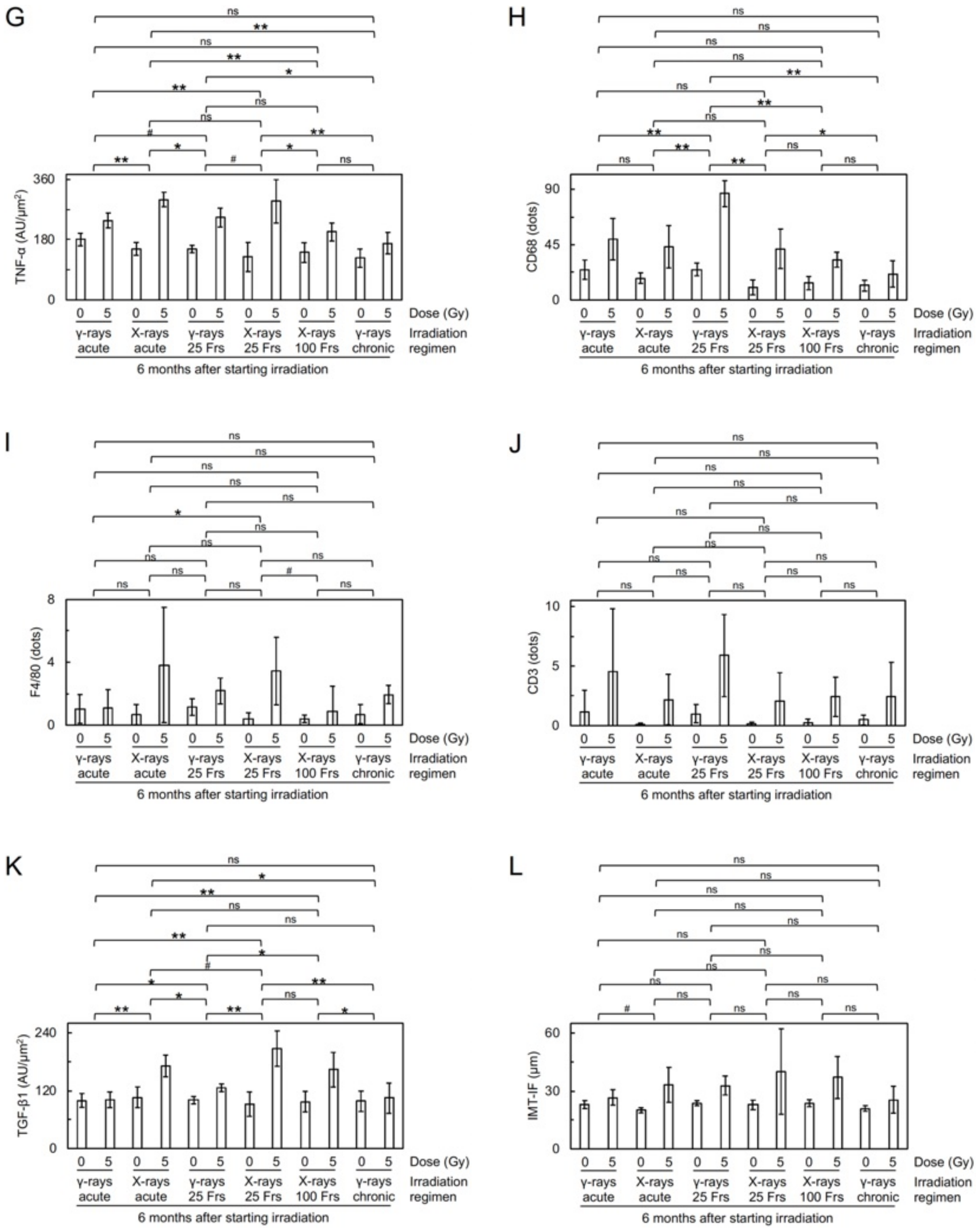
G
TNF-α (AU/μm²)
H
CD68 (dots)
I
F4/80 (dots)
J
CD3 (dots)
K
TGF-β1 (AU/μm²)
L
IMT-IF (μm)
Dose (Gy)
γ-rays acute
X-rays acute
γ-rays 25 Frs
X-rays 25 Frs
X-rays 100 Frs
γ-rays chronic
Irradiation regimen
6 months after starting irradiation

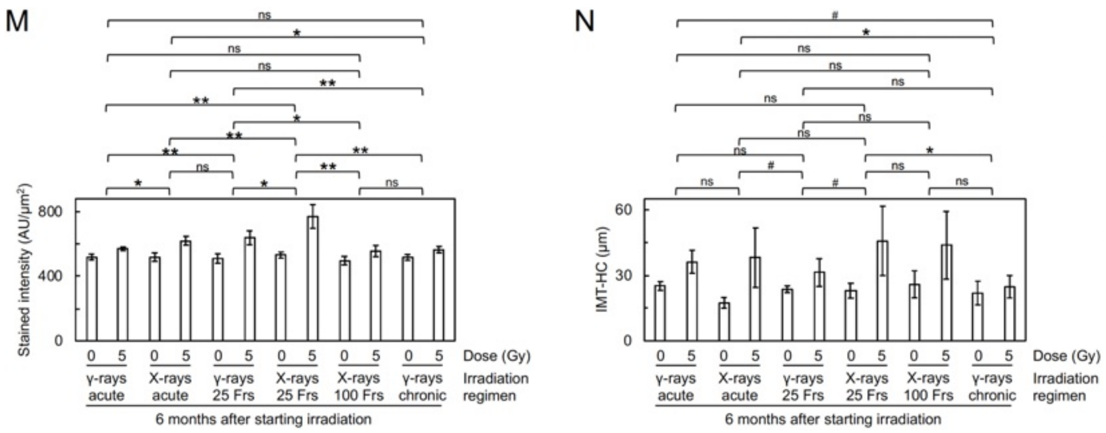

Figure S8. Comparisons of aortic endpoints among six irradiation regimens at 6 months after starting irradiation.

Comparisons between the two irradiation regimens (15 pairs for six irradiation regimens) were made for (**A**) CD31 negativity, (**B**) DAPI negativity, (**C**) eNOS, (**D**) VE-cadherin, (**E**) VECs with subcellular fragments, (**F**) VSMCs with subcellular fragments, (**G**) TNF-α, (**H**) CD68, (**I**) F4/80, (**J**) CD3, (**K**) TGF-β1, (**L**) IMT-IF, (**M**) intensity of aniline blue per unit aortic wall area, and (**N**) IMT-HC. *n* = 9–10 mice/group totaling 114–117 mice in 12 groups. Frs, fractions. *P* by the analysis of deviance. **, *P* <0.001. *, 0.001≤ *P* <0.05. #, 0.05≤ *P* <0.1 (marginally significant), ns, *P* ≥0.1 (nonsignificant), after Bonferroni corrections. Data presented in Figure S5 were replotted for further comparisons. See Tables S3, S12, S13, S20 and S21 for a series of the Kolmogorov–Smirnov tests to compare effects of various irradiation regimens.

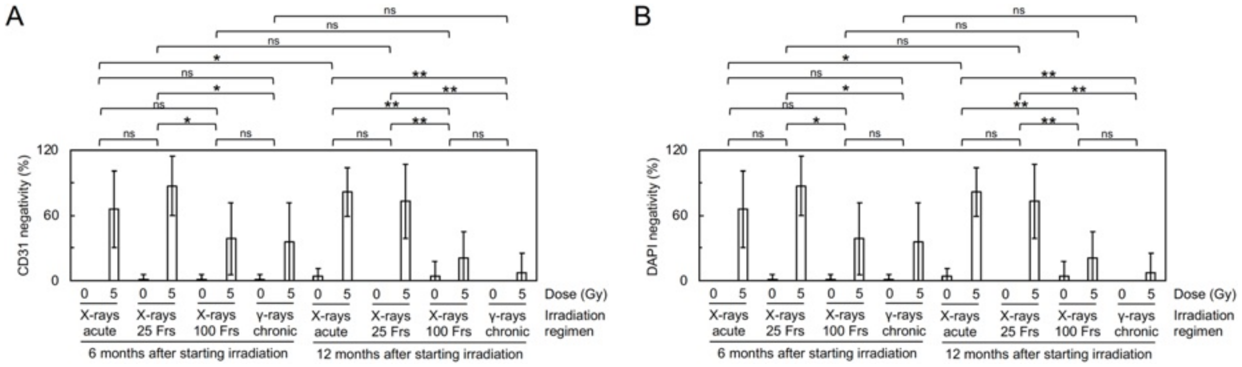

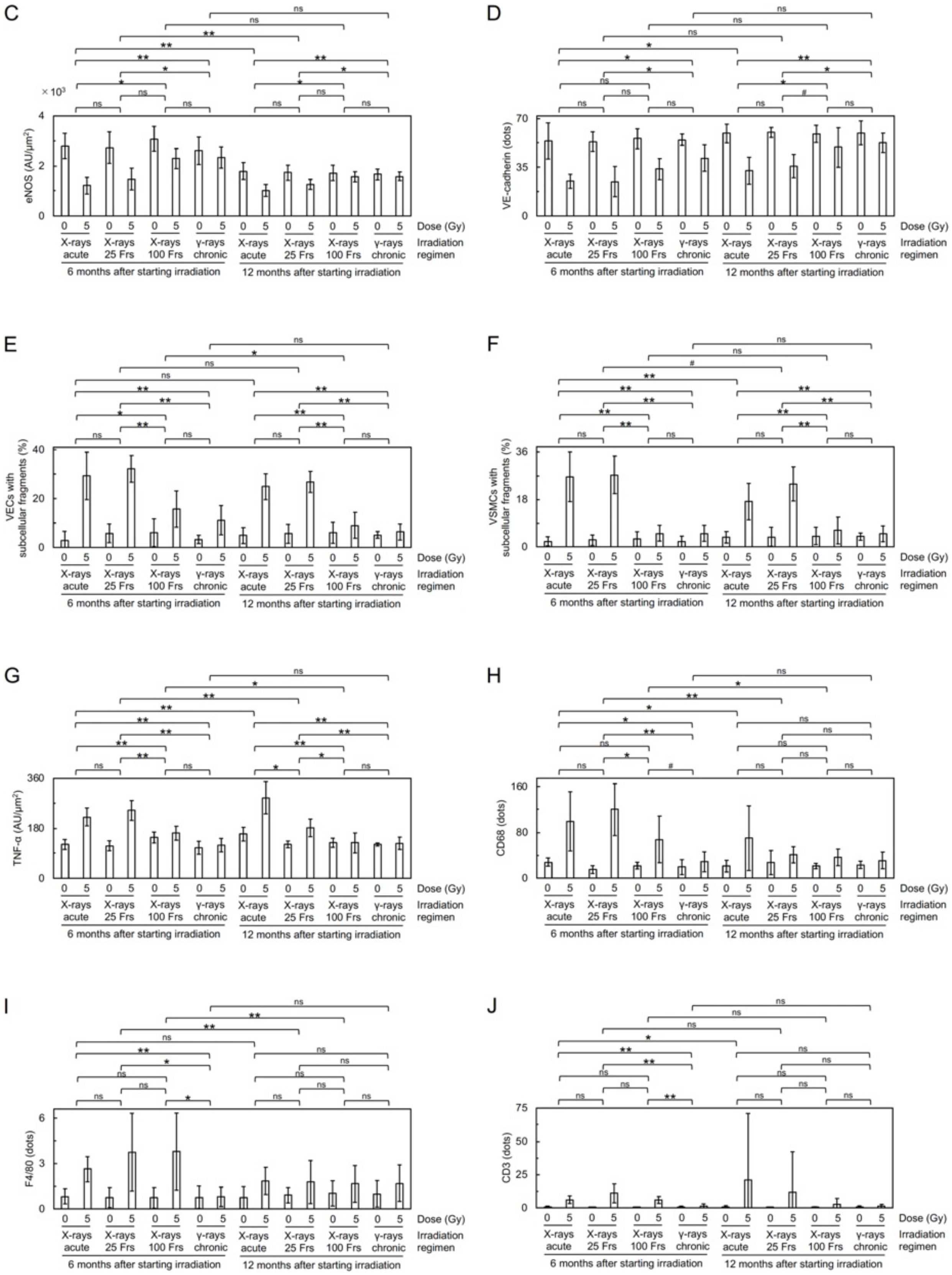
C
eNOS (AU/µm²)
× 10³
D
VE-cadherin (dots)
E
VECs with subcellular fragments (%)
F
VSMCs with subcellular fragments (%)
G
TNF-α (AU/µm²)
H
CD68 (dots)
I
F4/80 (dots)
J
CD3 (dots)
Dose (Gy)
Irradiation regimen
X-rays acute
X-rays 25 Frs
X-rays 100 Frs
γ-rays chronic
6 months after starting irradiation
12 months after starting irradiation

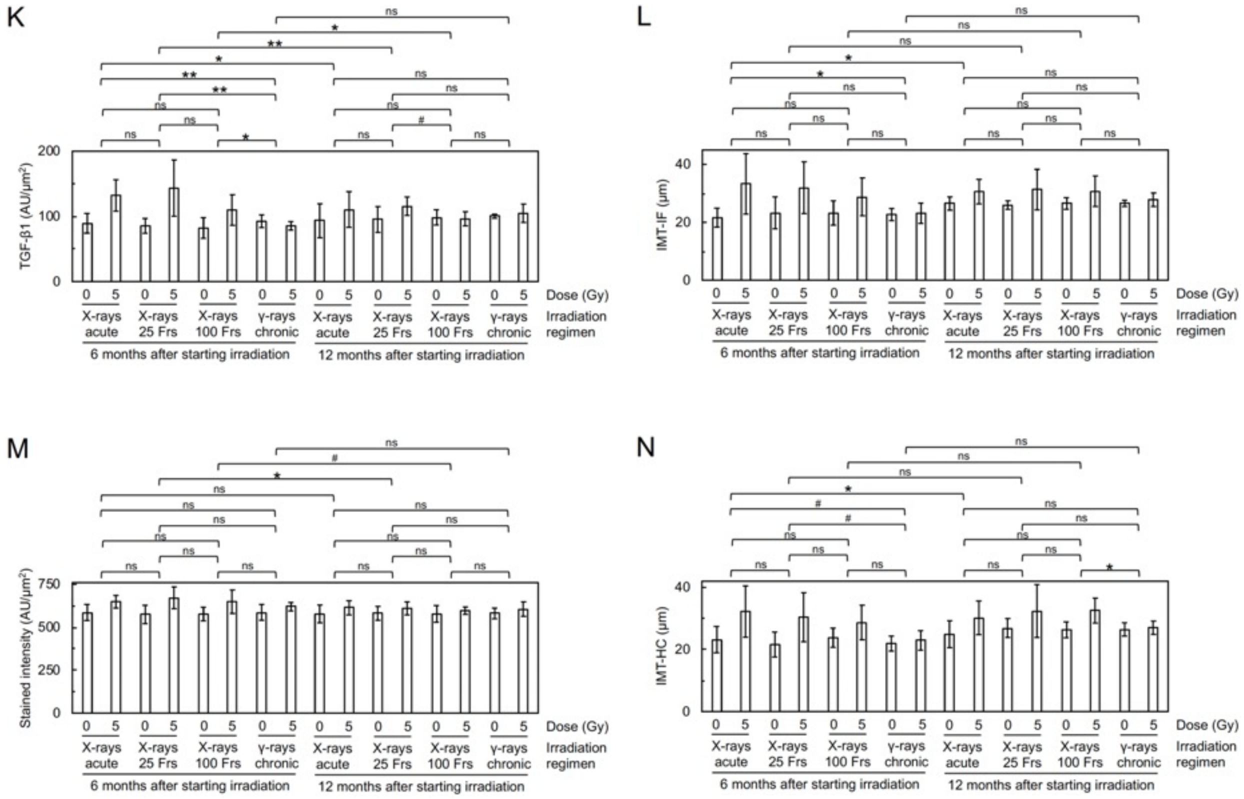

Figure S9. Comparisons of carotid endpoints among four irradiation regimens at 6 and 12 months after starting irradiation.

Comparisons between the two irradiation regimens (6 pairs for four irradiation regimens, each at 6 and 12 months after starting irradiation) or between the two timepoints in each irradiation regimen (6 vs 12 months after starting irradiation) were made for (**A**) CD31 negativity, (**B**) DAPI negativity, (**C**) eNOS, (**D**) VE-cadherin, (**E**) VECs with subcellular fragments, (**F**) VSMCs with subcellular fragments, (**G**) TNF-α, (**H**) CD68, (**I**) F4/80, (**J**) CD3, (**K**) TGF-β1, (**L**) IMT-IF, (**M**) intensity of aniline blue per unit carotid wall area, and (**N**) IMT-HC. n = 9–10 mice/group totaling 158 mice in 16 groups. Frs, fractions. P by the analysis of deviance, or the linear regression analysis. **, P <0.001. *, 0.001≤ P <0.05. #, 0.05≤ P <0.1 (marginally significant), ns, P ≥0.1 (nonsignificant), after Bonferroni corrections. Data presented in Figures 3 and 4 were replotted for further comparisons. See Tables S3, S6–S11 and S22–S27 for a series of the Kolmogorov–Smirnov tests to compare effects of various irradiation regimens.

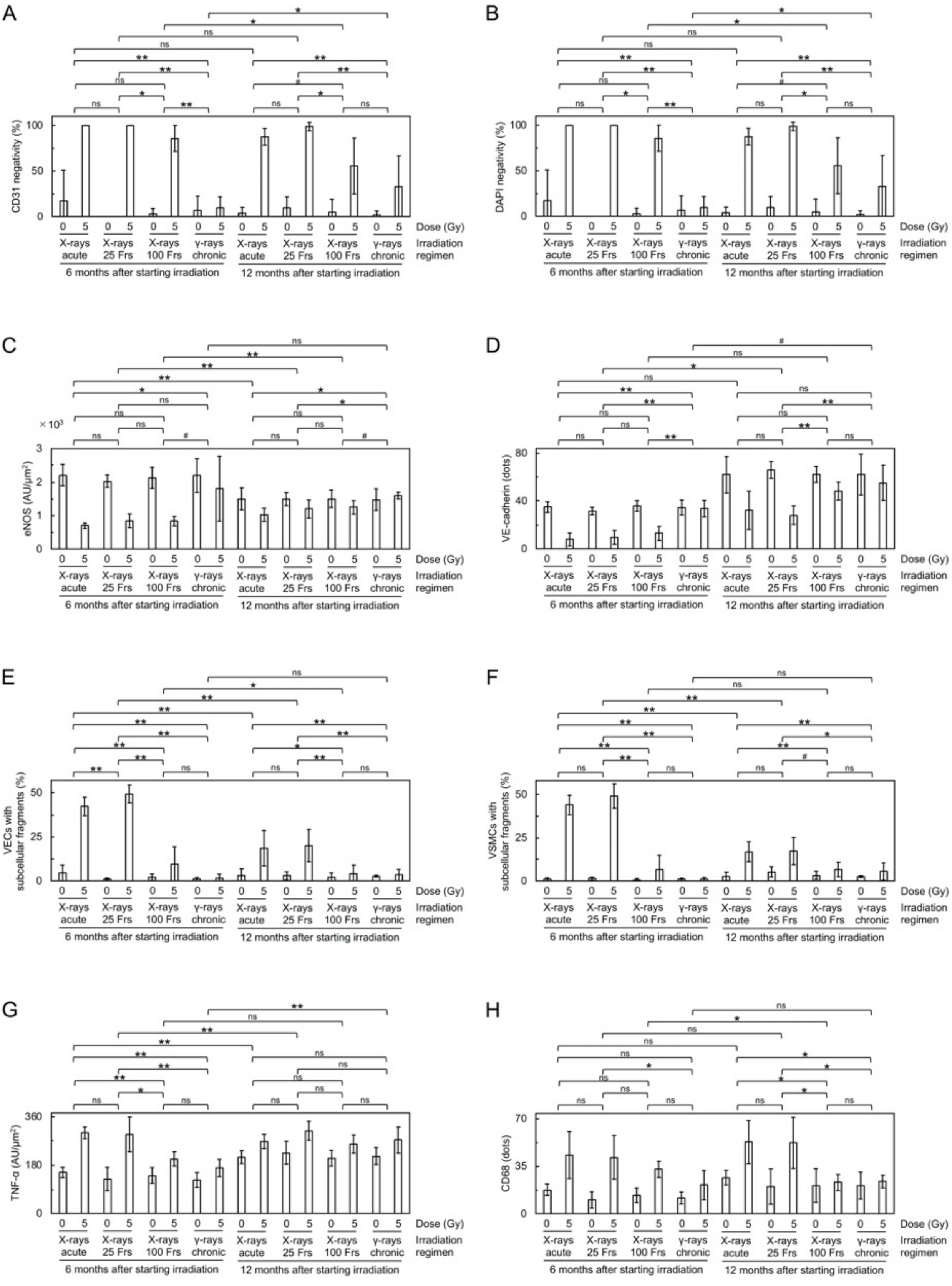
A
CD31 negativity (%)
B
DAPI negativity (%)
C
× 10^3
eNOS (AU/μm²)
D
VE-cadherin (dots)
E
VECs with subcellular fragments (%)
F
VSMCs with subcellular fragments (%)
G
TNF-α (AU/μm²)
H
CD68 (dots)
Dose (Gy)
Irradiation regimen
X-rays acute
X-rays 25 Frs
X-rays 100 Frs
γ-rays chronic
6 months after starting irradiation
12 months after starting irradiation
ns
*
**
#

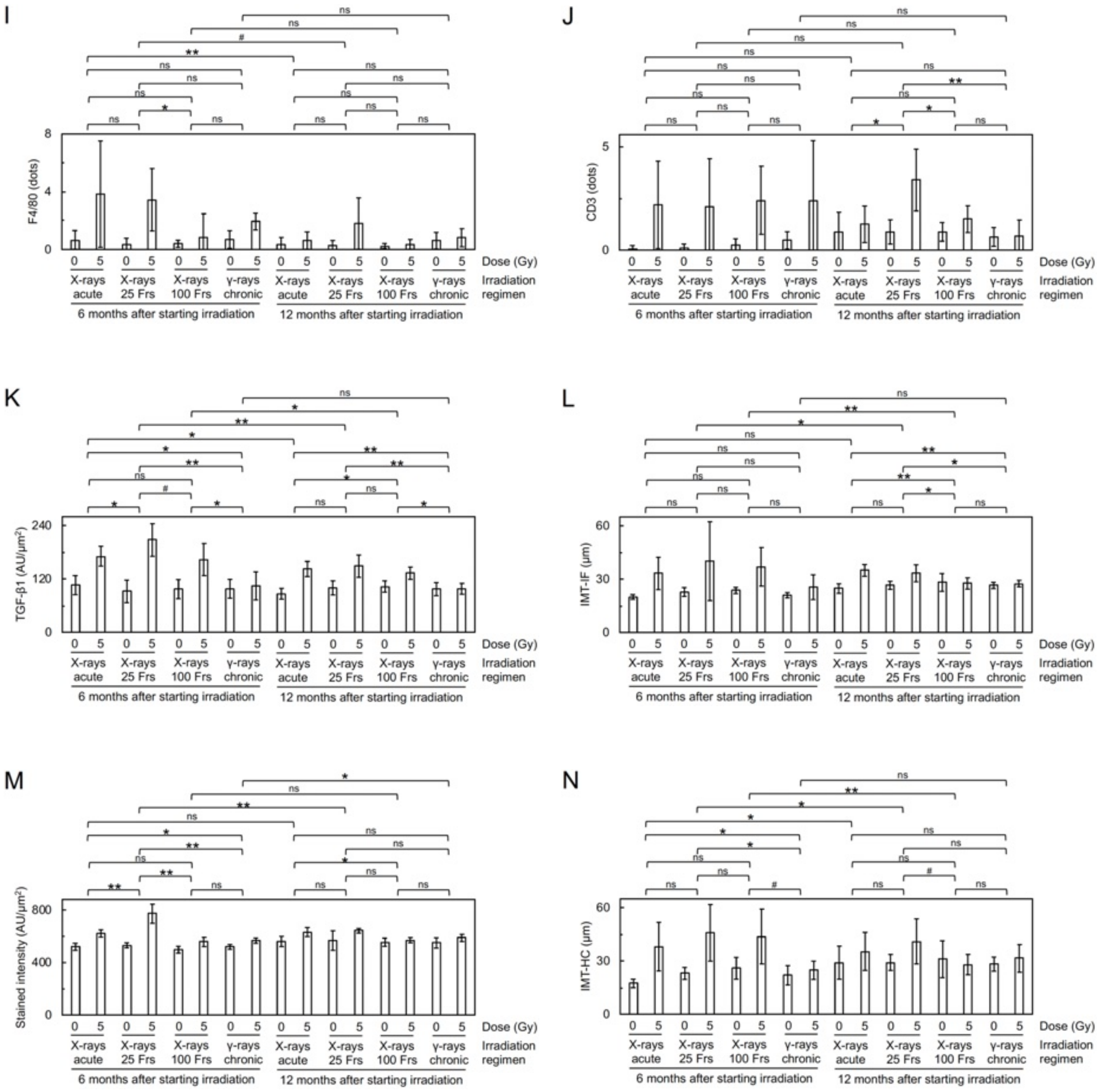

Figure S10. Comparisons of aortic endpoints among four irradiation regimens at 6 and 12 months after starting irradiation.

Comparisons between the two irradiation regimens (6 pairs for four irradiation regimens, each at 6 and 12 months after starting irradiation) or between the two timepoints in each irradiation regimen (6 vs 12 months after starting irradiation) were made for (**A**) CD31 negativity, (**B**) DAPI negativity, (**C**) eNOS, (**D**) VE-cadherin, (**E**) VECs with subcellular fragments, (**F**) VSMCs with subcellular fragments, (**G**) TNF-α, (**H**) CD68, (**I**) F4/80, (**J**) CD3, (**K**) TGF-β1, (**L**) IMT-IF, (**M**) intensity of aniline blue per unit aortic wall area, and (**N**) IMT-HC. n = 9–10 mice/group totaling 153−157 mice in 16 groups. Frs, fractions. P

by the analysis of deviance, or the linear regression analysis. **, P <0.001. *, 0.001≤ P <0.05. #, 0.05≤ P <0.1 (marginally significant), ns, P ≥0.1 (nonsignificant), after Bonferroni corrections. Data presented in Figure S5 were replotted for further comparisons. See Tables S3, S14–S19 and S22–S27 for a series of the Kolmogorov–Smirnov tests to compare effects of various irradiation regimens.

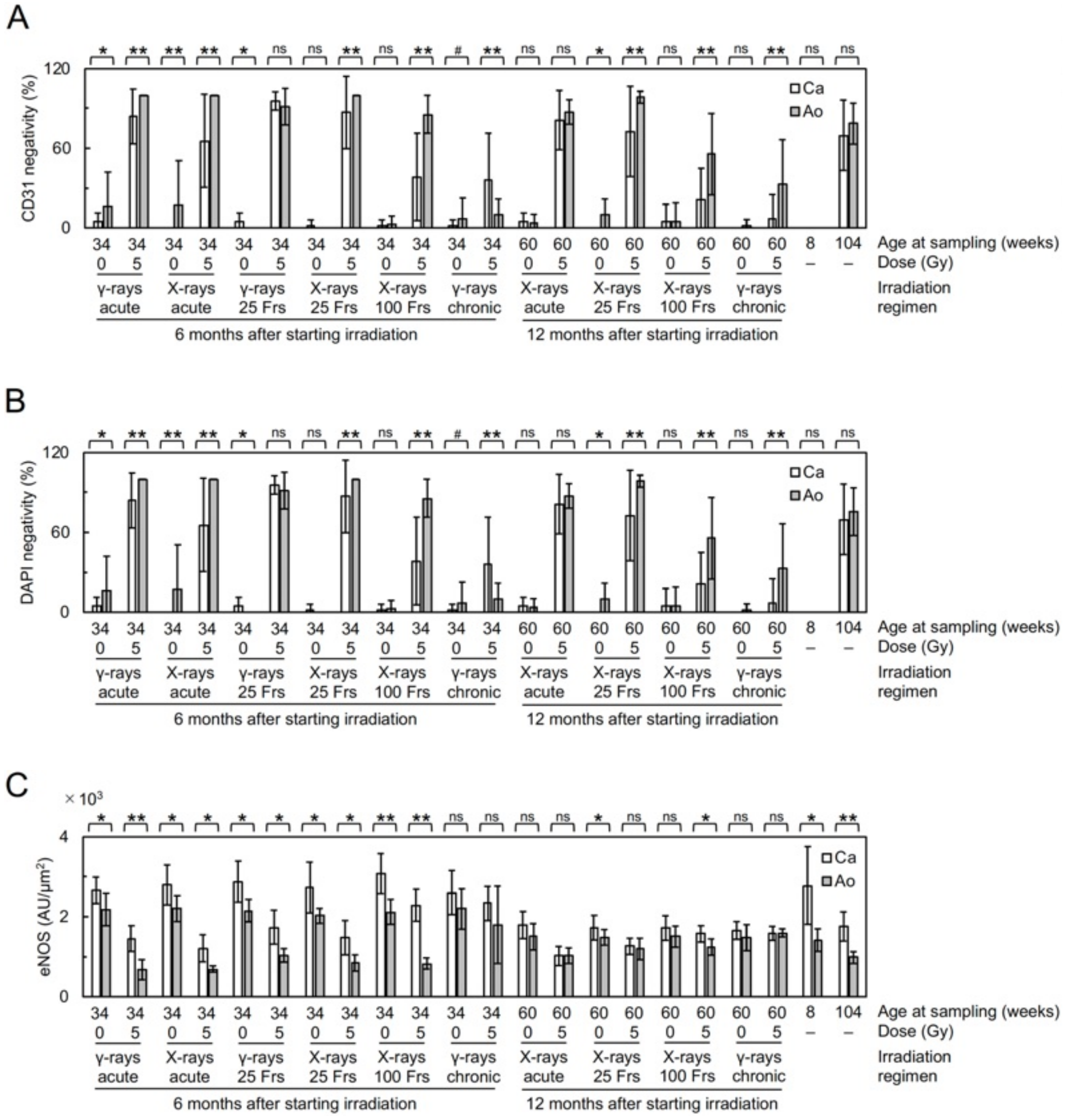

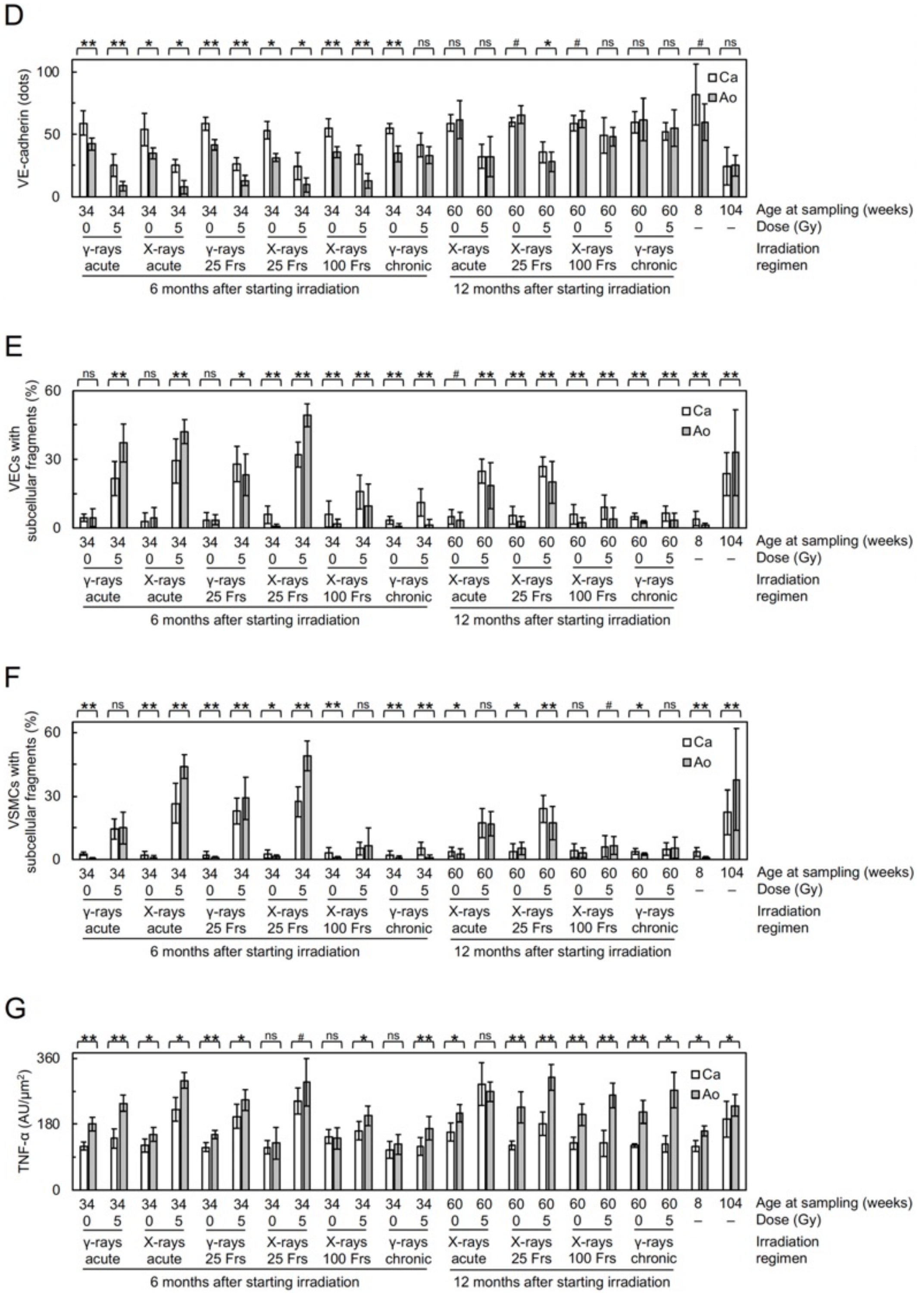
D
VE-cadherin (dots)
E
VECs with subcellular fragments (%)
F
VSMCs with subcellular fragments (%)
G
TNF-α (AU/μm²)
Ca
Ao
Age at sampling (weeks)
Dose (Gy)
Irradiation regimen
γ-rays acute
X-rays acute
γ-rays 25 Frs
X-rays 25 Frs
X-rays 100 Frs
γ-rays chronic
6 months after starting irradiation
12 months after starting irradiation

H

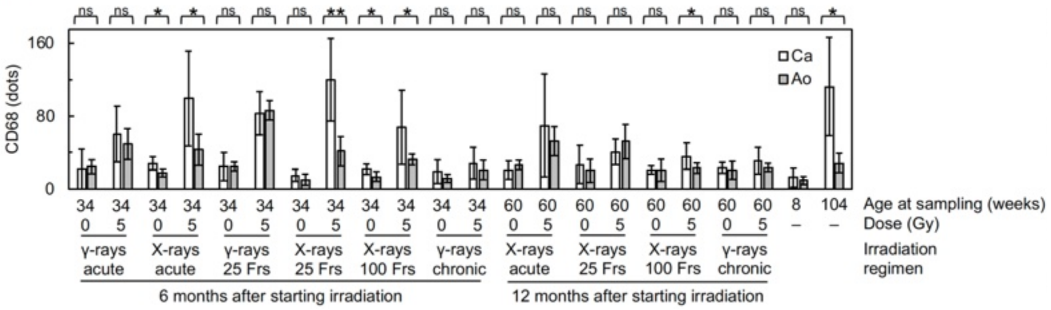
CD68 (dots)
160
80
0
Ca
Ao
Age at sampling (weeks)
Dose (Gy)
Irradiation regimen
γ-rays acute
X-rays acute
γ-rays 25 Frs
X-rays 25 Frs
X-rays 100 Frs
γ-rays chronic
6 months after starting irradiation
12 months after starting irradiation

I

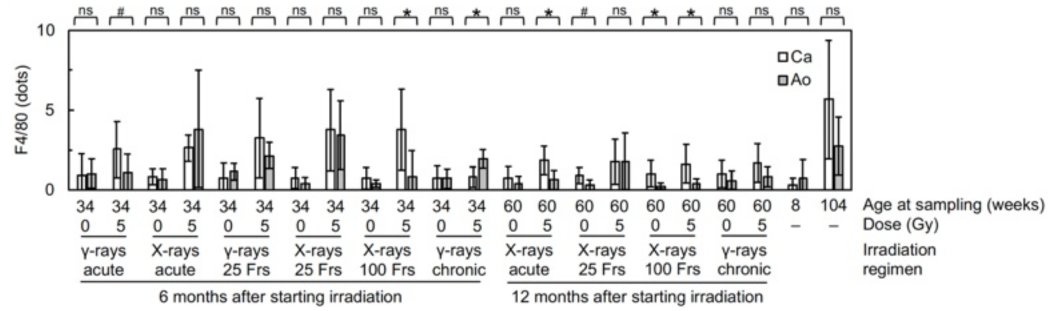
F4/80 (dots)
10
5
0
Ca
Ao
Age at sampling (weeks)
Dose (Gy)
Irradiation regimen
γ-rays acute
X-rays acute
γ-rays 25 Frs
X-rays 25 Frs
X-rays 100 Frs
γ-rays chronic
6 months after starting irradiation
12 months after starting irradiation

J

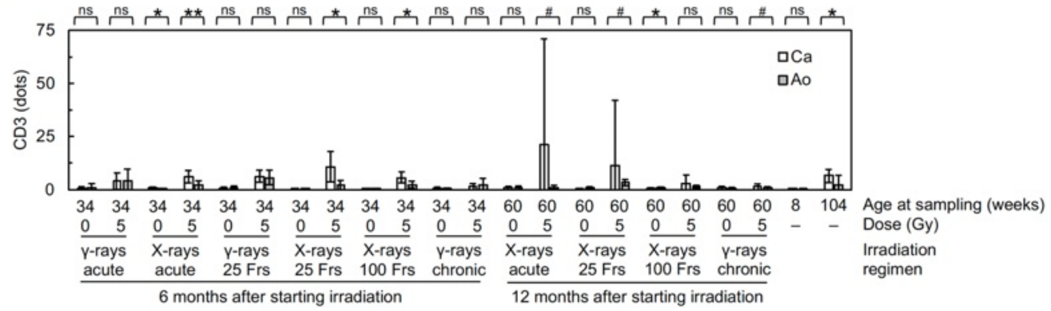
CD3 (dots)
75
50
25
0
Ca
Ao
Age at sampling (weeks)
Dose (Gy)
Irradiation regimen
γ-rays acute
X-rays acute
γ-rays 25 Frs
X-rays 25 Frs
X-rays 100 Frs
γ-rays chronic
6 months after starting irradiation
12 months after starting irradiation

K

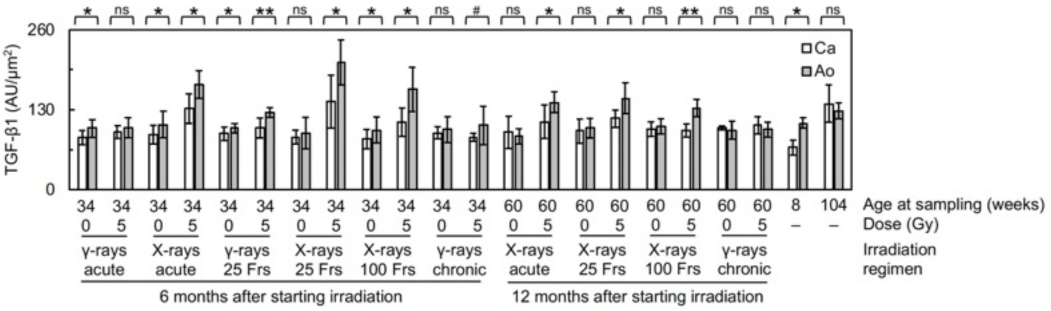
TGF-β1 (AU/μm²)
260
130
0
Ca
Ao
Age at sampling (weeks)
Dose (Gy)
Irradiation regimen
γ-rays acute
X-rays acute
γ-rays 25 Frs
X-rays 25 Frs
X-rays 100 Frs
γ-rays chronic
6 months after starting irradiation
12 months after starting irradiation

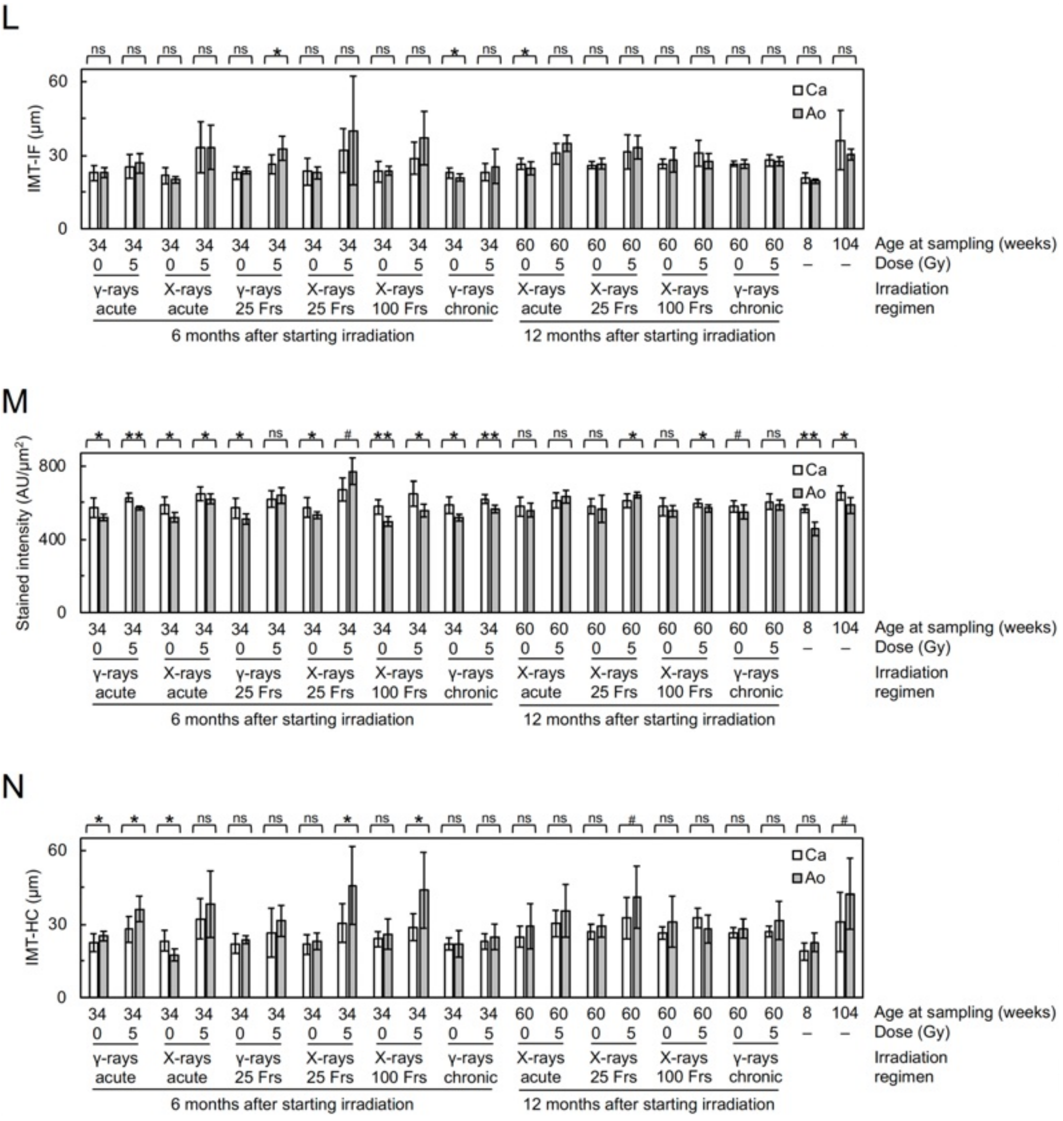

Figure S11. Comparisons between carotid and aortic changes in each group. Comparisons between the carotid and aortic changes in each of 22 groups were made for (**A**) CD31 negativity, (**B**) DAPI negativity, (**C**) eNOS, (**D**) VE-cadherin, (**E**) VECs with subcellular fragments, (**F**) VSMCs with subcellular fragments, (**G**) TNF-α, (**H**) CD68, (**I**) F4/80, (**J**) CD3, (**K**) TGF-β1, (**L**) IMT-IF, (**M**) intensity of aniline blue per unit wall area, and (**N**) IMT-HC. n = 7–10 mice/group totaling 210–215 mice in 22 groups. Ao, aorta. Ca, carotid. Frs, fractions. P by a paired-samples t-test, Wilcoxon signed rank test, or Wald test. **, P <0.001. *, 0.001≤ P <0.05. #, 0.05≤ P <0.1 (marginally significant), ns, P ≥0.1 (nonsignificant). Data presented in Figures 3, 4 and S5 were replotted for further comparisons.

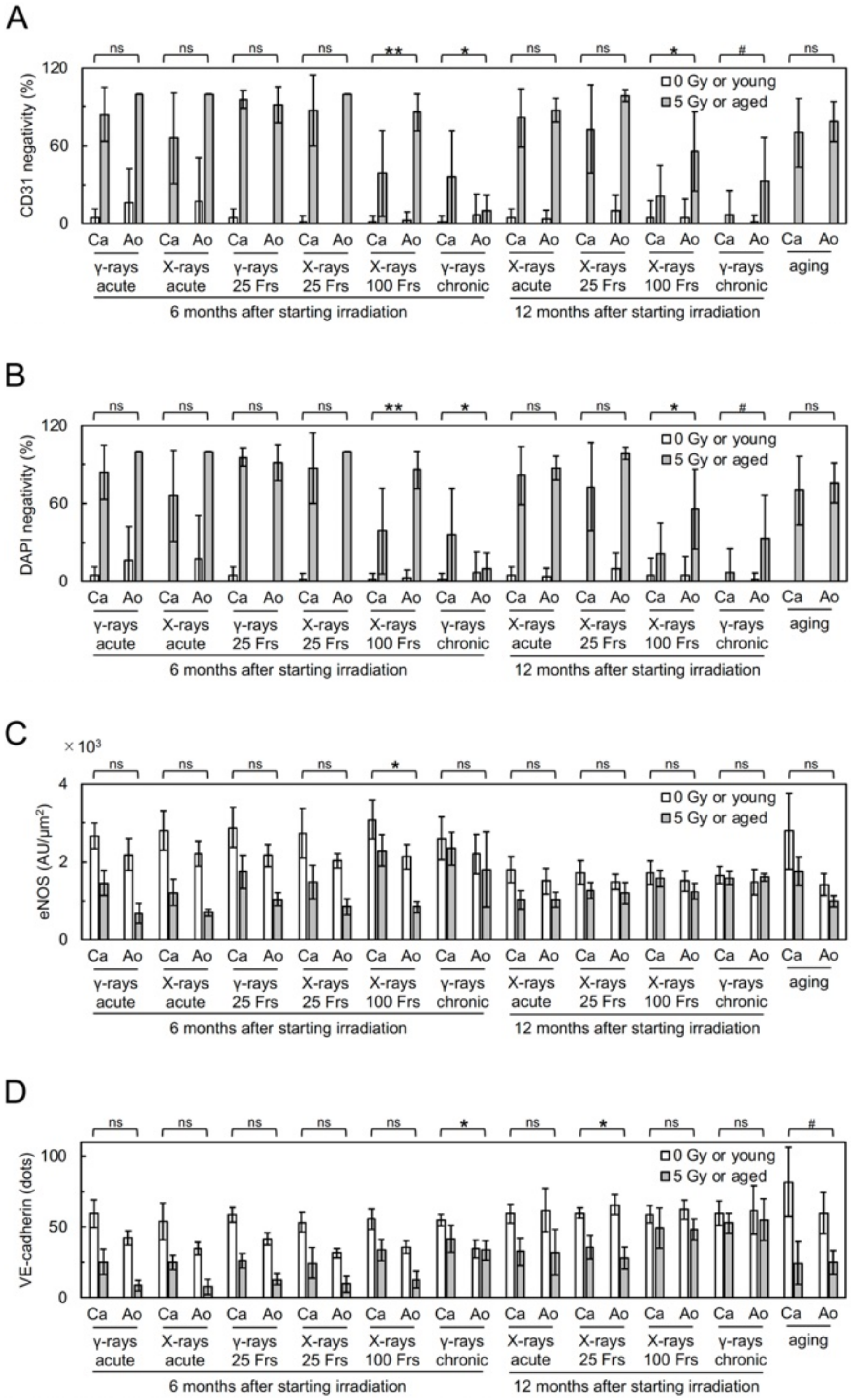
A
CD31 negativity (%)
0 Gy or young
5 Gy or aged
B
DAPI negativity (%)
C
× 10³
eNOS (AU/µm²)
D
VE-cadherin (dots)
ns
**
*
#
Ca Ao
γ-rays acute
X-rays acute
γ-rays 25 Frs
X-rays 25 Frs
X-rays 100 Frs
γ-rays chronic
aging
6 months after starting irradiation
12 months after starting irradiation

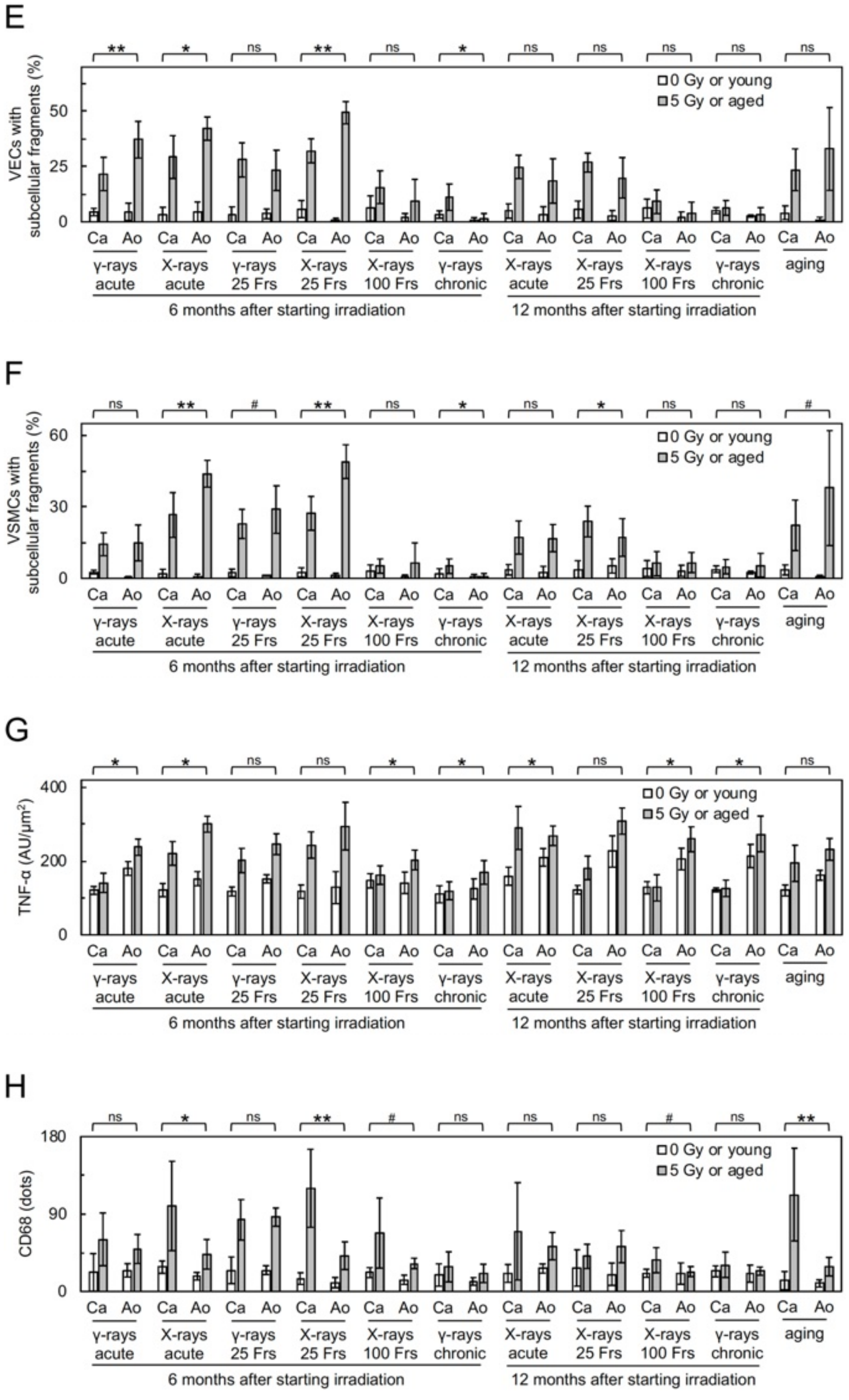
E
VECs with subcellular fragments (%)
F
VSMCs with subcellular fragments (%)
G
TNF-α (AU/μm²)
H
CD68 (dots)
0 Gy or young
5 Gy or aged
** * ns ** ns * ns ns ns ns ns
ns ** # ** ns * ns * ns ns #
* * ns ns * * * ns * * ns
ns * ns ** # ns ns ns # ns **
Ca Ao
γ-rays acute
X-rays acute
γ-rays 25 Frs
X-rays 25 Frs
X-rays 100 Frs
γ-rays chronic
X-rays acute
X-rays 25 Frs
X-rays 100 Frs
γ-rays chronic
aging
6 months after starting irradiation
12 months after starting irradiation

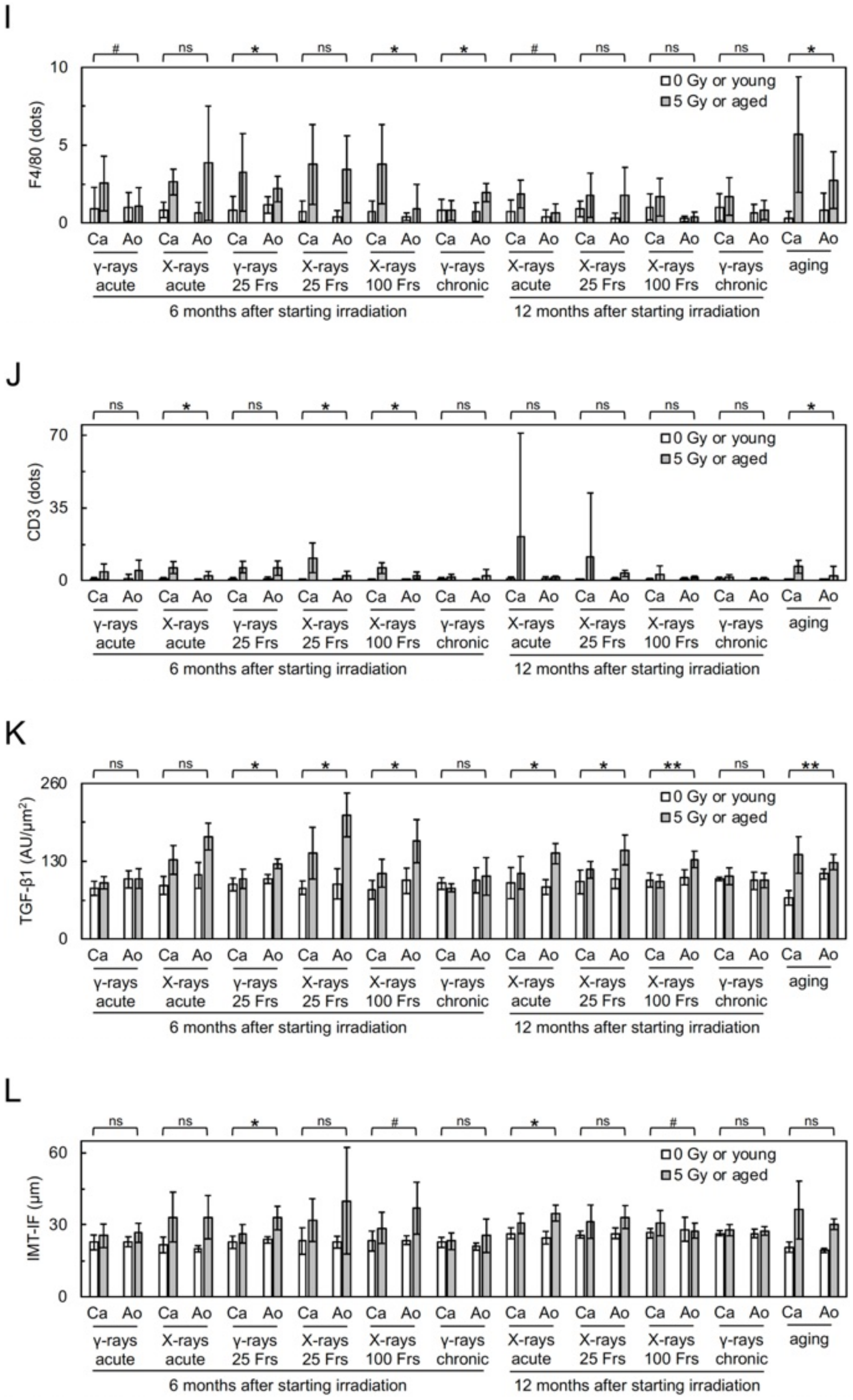
I
F4/80 (dots)
0 Gy or young
5 Gy or aged
J
CD3 (dots)
K
TGF-β1 (AU/μm²)
L
IMT-IF (μm)
Ca
Ao
γ-rays acute
X-rays acute
γ-rays 25 Frs
X-rays 25 Frs
X-rays 100 Frs
γ-rays chronic
aging
6 months after starting irradiation
12 months after starting irradiation
ns
#
*
**

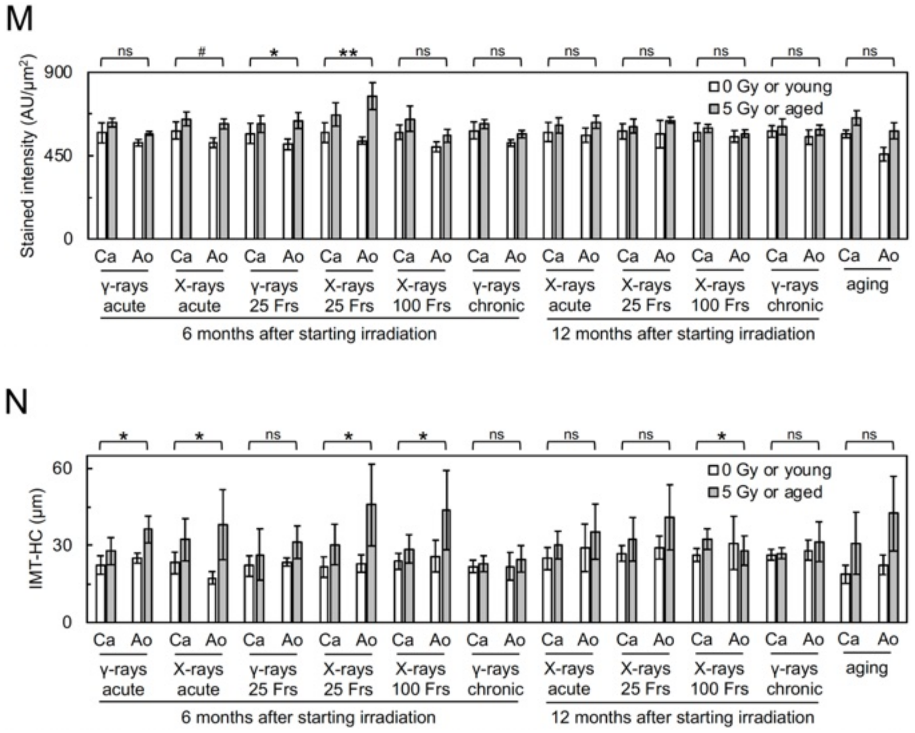

Figure S12. Comparisons between carotid and aortic changes in each irradiation regimen and aging.

Comparisons between the carotid and aortic changes in each irradiation regimen and aging were made for (**A**) CD31 negativity, (**B**) DAPI negativity, (**C**) eNOS, (**D**) VE-cadherin, (**E**) VECs with subcellular fragments, (**F**) VSMCs with subcellular fragments, (**G**) TNF-α, (**H**) CD68, (**I**) F4/80, (**J**) CD3, (**K**) TGF-β1, (**L**) IMT-IF, (**M**) intensity of aniline blue per unit wall area, and (**N**) IMT-HC. *n* = 7–10 mice/group totaling 210–215 mice in 22 groups. Ao, aorta. Ca, carotid. Frs, fractions. *P* by the linear regression analysis. **, $P < 0.001$. *, $0.001 \leq P < 0.05$. #, $0.05 \leq P < 0.1$ (marginally significant), ns, $P \geq 0.1$ (nonsignificant). Data presented in Figures 3, 4 and S5 were replotted for further comparisons. See Tables 2 and S28 for the results of the statistical analysis to compare carotid and aortic changes in each irradiation regimen or among six irradiation regimens.

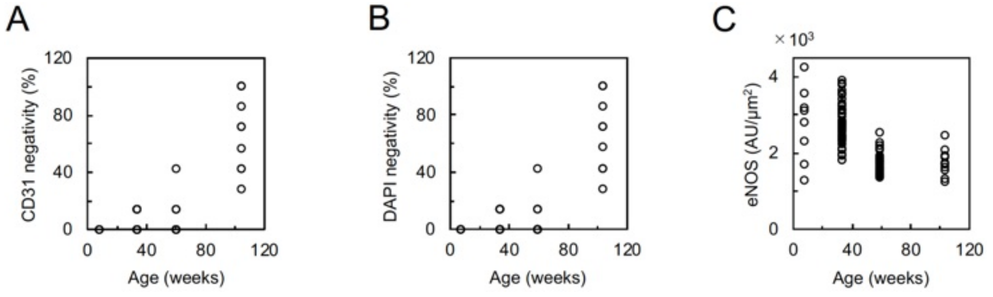

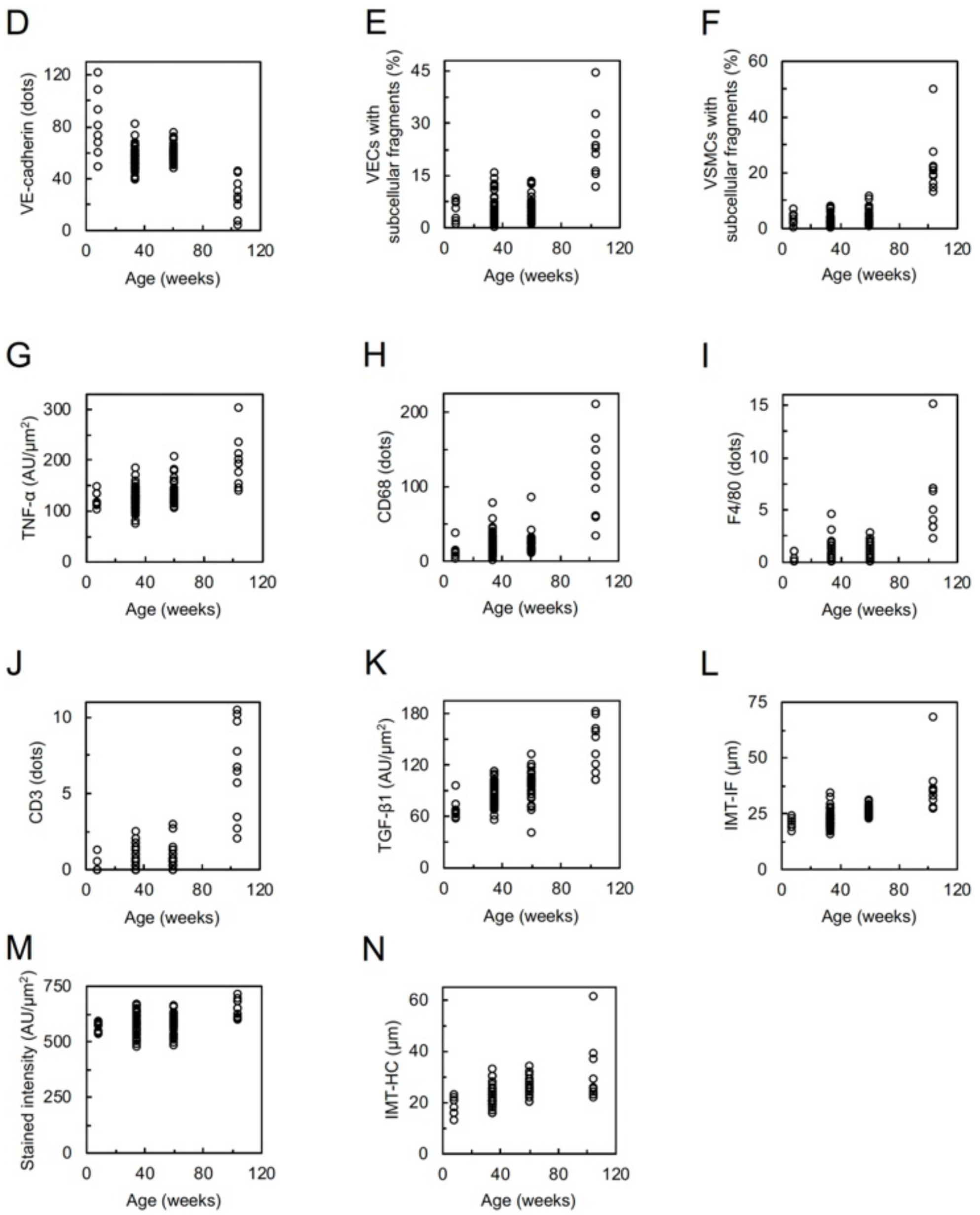

Figure S13. Changes in carotid endpoints as a function of age.

Carotid changes in sham- or nonirradiated mice are plotted as a function of age for (**A**) CD31 negativity, (**B**) DAPI negativity, (**C**) eNOS, (**D**) VE-cadherin, (**E**) VECs with subcellular fragments, (**F**) VSMCs with subcellular fragments, (**G**) TNF-α, (**H**) CD68, (**I**) F4/80, (**J**) CD3, (**K**) TGF-β1, (**L**) IMT-IF, (**M**) intensity of aniline blue per unit carotid wall area, and (**N**) IMT-HC. Each datapoint represents data from an individual mouse of which data in each group are shown in Figures 3 and 4. n = 8 mice from a single group of nonirradiated mice for 8 weeks of age. n = 59 mice from six groups of sham-irradiated mice for 34 weeks of age. n = 39 mice from four groups of sham-irradiated mice for 60

weeks of age. n = 10 mice from a single group of nonirradiated mice for 104 weeks of age. See Table S30 for data approximation.

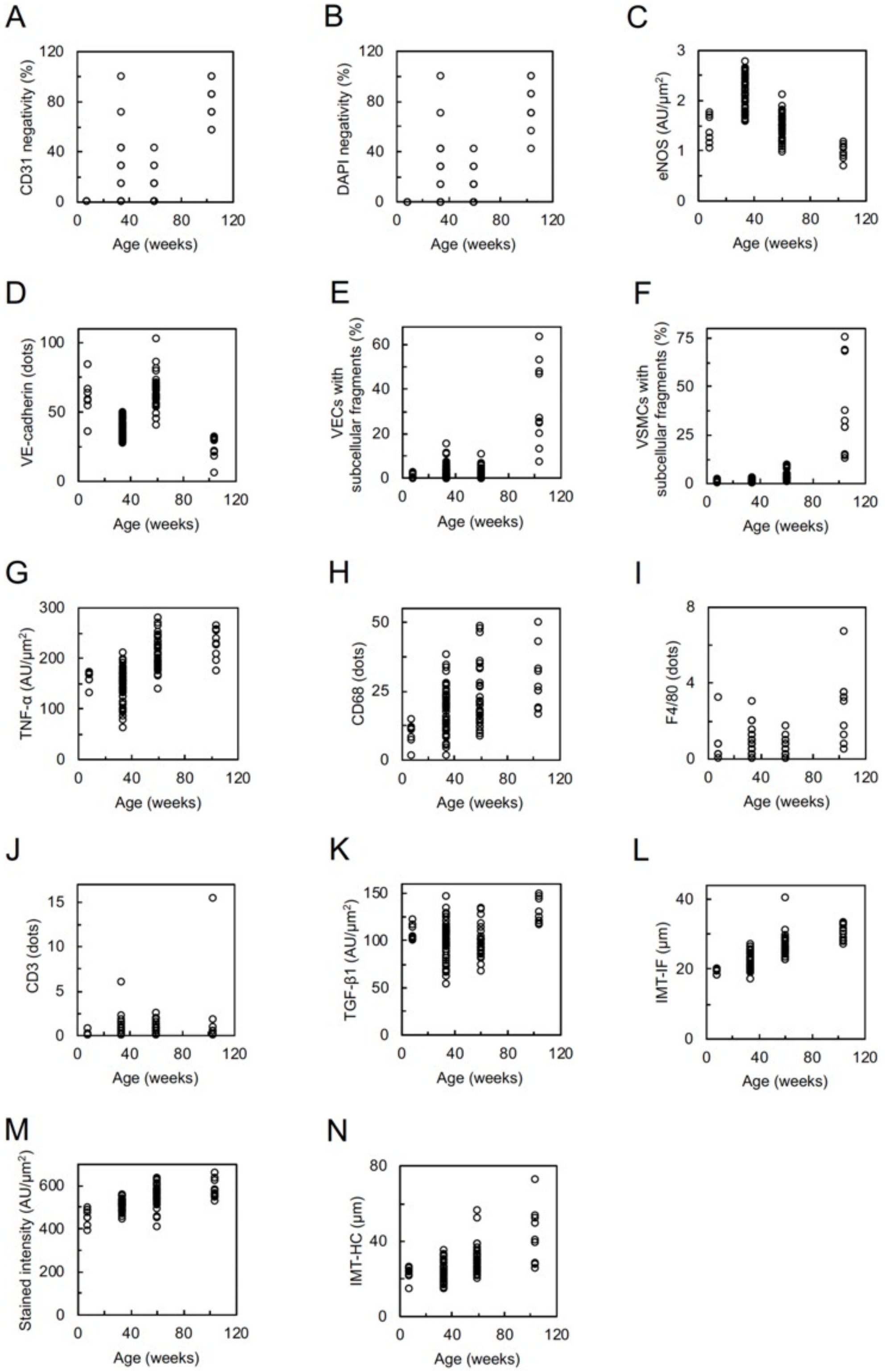

Figure S14. Changes in aortic endpoints as a function of age in sham- or nonirradiated mice.

Aortic changes in sham- or nonirradiated mice are plotted as a function of age for (**A**) CD31 negativity, (**B**) DAPI negativity, (**C**) eNOS, (**D**) VE-cadherin, (**E**) VECs with subcellular fragments, (**F**) VSMCs with subcellular fragments, (**G**) TNF-α, (**H**) CD68, (**I**) F4/80, (**J**) CD3, (**K**) TGF-β1, (**L**) IMT-IF, (**M**) intensity of aniline blue per unit aortic wall area, and (**N**) IMT-HC. Each datapoint represents data from an individual mouse of which data in each group are shown in Figure S5. n = 7–8 mice from a single group of nonirradiated mice for 8 weeks of age. n = 58–60 mice from six groups of sham-irradiated mice for 34 weeks of age. n = 35–39 mice from four groups of sham-irradiated mice for 60 weeks of age. n = 10 mice from a single group of nonirradiated mice for 104 weeks of age. See Table S30 for data approximation.
